# Online Training on Skin Cancer Diagnosis in Rheumatologists: Results from a Nationwide Randomized Web-Based Survey

**DOI:** 10.1371/journal.pone.0127564

**Published:** 2015-05-21

**Authors:** Manuelle Viguier, Stéphanie Rist, François Aubin, Marie-Thérèse Leccia, Marie-Aleth Richard, Marina Esposito-Farèse, Philippe Gaudin, Thao Pham, Pascal Richette, Daniel Wendling, Jean Sibilia, Florence Tubach, Club Rhumatismes et Inflammation

**Affiliations:** 1 Université Paris Diderot, Sorbonne Paris Cité, Paris, France; Assistance-Publique Hôpitaux de Paris (APHP), Hôpital Saint-Louis, Service de Dermatologie, Paris, France; 2 Service de Rhumatologie, Centre Hospitalier Régional (CHR), Orléans, France; 3 Université de Franche Comté, EA3181, SFR4234, Service de Dermatologie, Centre Hospitalier Universitaire (CHU), Besançon, France; 4 Clinique de Dermatologie, Allergologie et Photobiologie, CHU, Grenoble, France; 5 Service de Dermatologie, Université Aix-Marseille, Centre de recherche en oncologie biologique et oncopharmacologie, Hôpital de la Timone, Assistance Publique Hôpitaux de Marseille, Marseille, France; 6 Université Paris Diderot, Sorbonne Paris Cité, UMR 1123, Paris, France; APHP, Hôpital Bichat, Département d’Epidémiologie et Recherche Clinique, Centre de Pharmaco-épidémiologie (Céphépi), Paris, France; INSERM, CIC-EC 1425, UMR 1123, Paris, France; 7 Clinique Universitaire de Rhumatologie, CHU Hôpital Sud, Grenoble, France; 8 Service de Rhumatologie, CHU, APHM, Marseille, France; 9 Université Paris Diderot, APHP, Hôpital Lariboisière, Service de Rhumatologie, Paris, France; 10 Université de Franche Comté, Service de Rhumatologie, CHU, Besançon, France; 11 INSERM UMR_S1109, Centre de Recherche d’Immunologie et d’Hématologie, Fédération de médecine translationelle, Université de Strasbourg, Strasbourg, France; Queen Mary Hospital, HONG KONG

## Abstract

Patients with inflammatory rheumatisms, such as rheumatoid arthritis, are more prone to develop skin cancers than the general population, with an additional increased incidence when receiving TNF blockers. There is therefore a need that physicians treating patients affected with inflammatory rheumatisms with TNF blockers recognize malignant skin lesions, requiring an urgent referral to the dermatologist and a potential withdrawal or modification of the immunomodulatory treatment. We aimed to demonstrate that an online training dedicated to skin tumors increase the abilities of rheumatologists to discriminate skin cancers from benign skin tumors. A nationwide randomized web-based survey involving 141 French rheumatologists was conducted. The baseline evaluation included short cases with skin lesion pictures and multiple choice questions assessing basic knowledge on skin cancers. For each case, rheumatologists had to indicate the nature of skin lesion (benign; premalignant/malignant), their level of confidence in this diagnosis (10-points Likert scale), and the precise dermatological diagnosis among 5 propositions. Different scores were established. After randomization, only one group had access to the online formation consisting in 4 e-learning modules on skin tumors, of 15 minutes each (online training group). After reevaluation, the trained and the non-trained group (control group) were compared. The primary end-point was the number of adequate diagnoses of the nature of the skin lesions. The mean number of adequate diagnosis for the benign versus premalignant/malignant nature of the lesions was higher in the online training group (13.4 vs. 11.2 points; *p* value <0.0001). While the other knowledge scores were also significantly higher, no statistical difference was observed on the level of self-confidence between the 2 groups. In conclusion, the online formation was effective to improve the rheumatologists’ ability to diagnose skin cancer.

## Introduction

A baseline increased risk of skin cancers in Rheumatoid arthritis (RA) patients compared to the general population has been reported, with a 40% increase risk of squamous cell carcinoma (SCC) and a 30% relative increase in basal cell carcinoma (BCC), and a further increase in patients receiving TNF inhibitors [1–8]. Meta-analysis from 4 prospective observational studies in RA patients showed a pooled risk estimate for non-melanoma skin cancers in patients receiving TNF blockers of 1.33 (95%CI 1.06 to 1.60), with similar results observed in a meta-analysis of randomized controlled trials (RR 2.02, 95%CI 1.11 to 3.95) [5, 7]. Two registries showed about a 2-fold increased risk of developing melanoma when receiving TNF inhibitor [6, 8].

Accordingly, several national and international recommendations regarding skin cancers have been established for patients affected with inflammatory rheumatism [9, 10].

Because of a limited access to a dermatologist in several countries [11, 12], there is therefore a need that physicians treating patients affected with inflammatory rheumatisms with TNF blockers discriminate benign skin tumors from malignant skin lesions, requiring an appropriate referral to the dermatologist and a potential withdrawal or modification of the immunomodulatory treatment.

In the present study, we aimed to demonstrate whether an online course dedicated to the recognition of the most frequent benign and premalignant/malignant skin tumors increased the rheumatologists’ ability to identify these lesions.

## Materials and Methods

A nationwide randomized web-based survey was conducted online between October 1^st^, 2012 and October 1^st^, 2013. Starting from the French registry of rheumatologists (CEGEDIM registry), 420 rheumatologists all over France were solicited via e-mail. Written participant consent or institutional review board approval was not required because French law considers that it is not mandatory for non-interventional research and because this study did not involve patients but medical practitioners. No health or other identifying information was collected from the participants. All data collected (see S1 Text) were anonymized prior to author access and analysis. Physicians did not receive any financial or non-financial incitatives for participating in the survey.

Rheumatologists performed an initial online evaluation (Test 1, see S1 File), composed and scored as follows:

- 20 clinical cases (short text and pictures of skin lesions), for which participants had to indicate (i) if the skin lesion(s) were benign or premalignant/malignant (Score 1; range 0–20; 0: no adequate diagnosis; 20: adequate diagnosis for all cases), (ii) their level of confidence in this diagnosis (benign or premalignant/malignant) measured on a 10-points Likert scale (Score 2; range 0–10) and (iii) to identify the precise diagnosis of the skin lesion(s) among 5 diagnoses (Score 3; range 0–20, 0: no correct diagnosis; 20: correct diagnosis for all cases)
- 5 multiple choice questionnaires of 5 response modalities each, testing the basic knowledge regarding skin cancers such as risk factors, adequate modalities of sun protection, prognosis of the different types of skin cancer, management of TNF blockers in case of history or diagnosis of skin cancer (Score 4; range 0–25, 0: no correct answer; 25: 100% correct answers).

The clinical cases included the most frequent benign and premalignant/malignant skin tumors and were selected after unanimous validation by the board of dermatologists and rheumatologists. All pictures of skin lesions used were selected and presented in a way that did not allow the identification of the patient and the participants did not have any access to identify the patient. All patients provided a written consent before being photographed, allowing the use of the picture for educational purpose.

After Test 1, rheumatologists were randomized into 2 arms through the web site randomization module to ensure allocation concealment. Only participants randomized in the experimental arm received an online formation on skin tumors (Online training group), and attended consecutively 4 e-learning modules of 15 minutes each, consisting on a slide-show commented by a dermatologist of the board (http://www.cri-net.com/formation/reussite.asp), that they could split into several sessions (Module 1: Frequent benign skin tumors; Module 2: Risk factors and prevention of skin cancers; Module 3: Frequent skin cancers; Module 4: Prognosis of the different skin cancers). The formation was planned to be performed over a 3-weeks period after the baseline evaluation.

Participants were reevaluated (Test 2, similar to Test 1, but in a different order) 3 weeks after the end of the formation (online training group) and the initial evaluation (control group). The primary end-point was Score 1 (diagnosis of the benign *vs* premalignant/malignant nature of the lesions) at Test 2. The secondary end-points were Scores 2 to 4.

### Sample size calculation

A sample size of 70 rheumatologists per group was planned to detect a mean difference in the number of adequate diagnosis of 1 point (out of 20), assuming the common standard deviation of 1.8 with a 0.05 two-sided significance level with 90% power.

### Analysis

The analysis was conducted on the intention-to-treat population (each randomized rheumatologist contributed to the initial group he was assigned). Comparisons of the mean scores obtained on Test 2 between trial arms were performed using a Student t test. Missing answers on Tests 2 were imputed by the corresponding answers on Test 1. Sensitivity analyses were performed to assess the impact of the treatment of missing values. Mean scores on Test 1 were compared by Student t test.

All statistical analyses were two-sided. The Type I error was fixed at 0.05. Statistical analyses were performed using R software v3.0.

## Results

**Fig 1:**
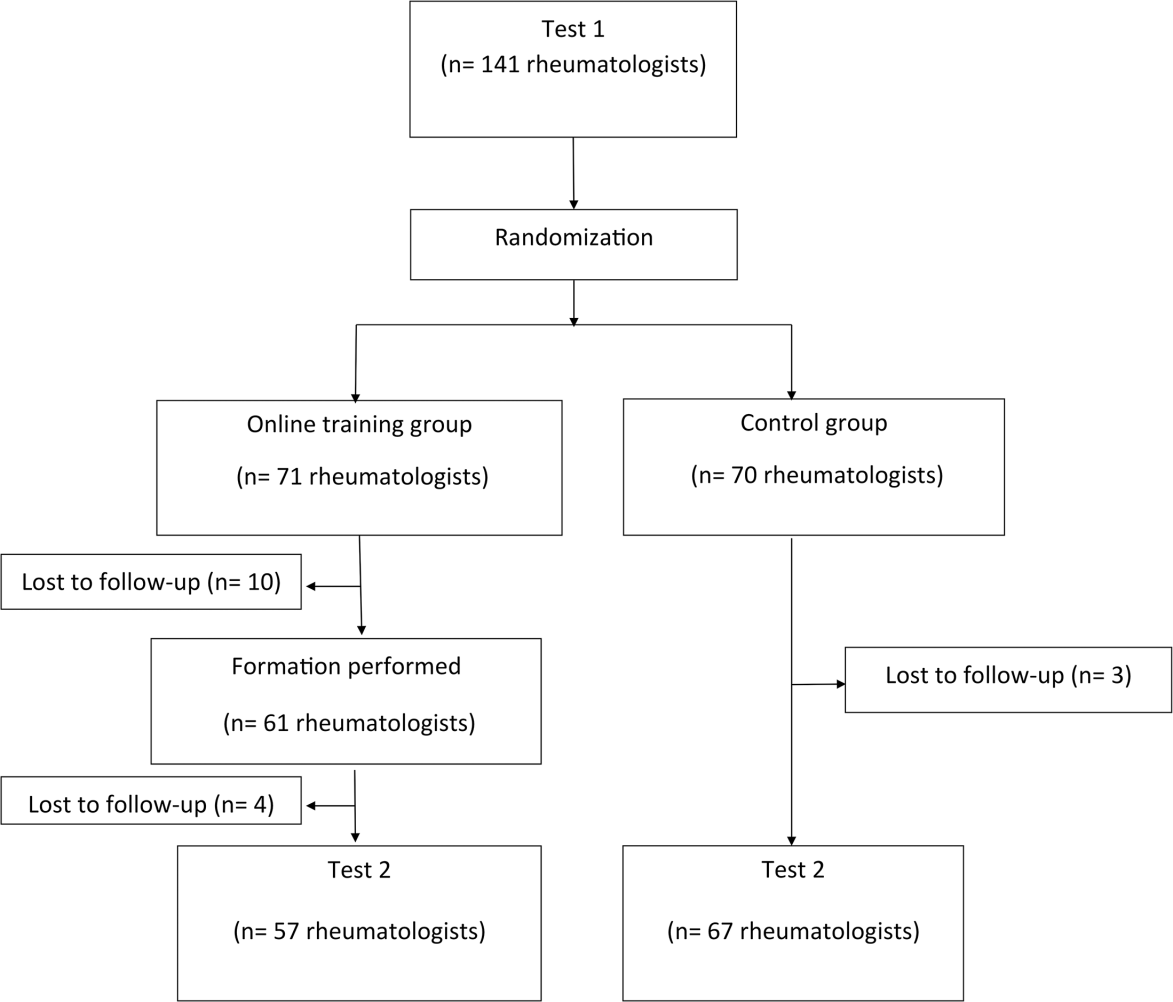
Study flow chart.

Altogether, 141 rheumatologists participated in the study (Fig 1), corresponding to a response rate of 34%. They were 74 females (52%), aged 44.9 years±11.1 (mean±SD), with 15.4±10.5 years of practice (mean±SD). The practice modalities was hospital practice in 74 (52%), private practice in 28 (20%) and 39 (28%) had a mixed hospital and private practice. Based on the declarations of participants, search for skin cancer risk factors, skin examination, or systematic yearly referral of patients with inflammatory rheumatisms to a dermatologist were rarely performed in patients receiving only conventional Disease Modifying Anti-Rheumatic Drugs (cDMARDs) (Table 1). Only 33% of rheumatologists give frequently photoprotection counseling, while, on the other hand, counseling patients regarding smoking cessation was performed by 85% of rheumatologists (not shown). In this subset of patients, the skin management was essentially left to the general practitioner, rather than to the dermatologist. In patients receiving biotherapy, search for skin cancer risk factors was performed by 63% of rheumatologists and half of them referred these patients to a dermatologist before starting biotherapy, and once a year thereafter. Only a third of rheumatologists performed by themselves skin examination before starting or during biotherapy (Table 1).

**Table 1:** Rheumatologists’ habits regarding skin management in their patients.

Do you	Very often/Always	Never/Not often/Sometimes/Only if reported by the patient
Search for skin cancer risk factors in all patients?	16%	84%
Search for skin cancer risk factors in patients with inflammatory rheumatism?	35%	65%
Search for skin cancer risk factors in patients with inflammatory rheumatism treated with BT?	63%	37%
Perform complete skin examination before starting BT?	33%	67%
Refer to dermatologist before starting BT?	53%	47%
Perform skin examination during BT?	36%	64%
Refer to dermatologist once a year during BT?	48%	49%
Refer to GP for skin examination during BT?	30%	70%
Perform skin examination during DMARDs?	20%	80%
Refer to dermatologist once a year during DMARDs?	4.3%	95.7%
Refer to GP for skin examination during DMARDs?	65%	35%

BT: biotherapy treatment; GP: general practitioner; DMARDs: Disease Modifying Anti-Rheumatic Drugs

### Baseline recognition of skin tumors by rheumatologists

No significant differences on the different scores were found between groups at baseline, on Test 1 (Table 2).

**Table 2:** Baseline evaluation of participating rheumatologists (Test 1).

Scores (range)	Online training group (n = 71)	Control group (n = 70)		
	Mean score	Mean score	[CI 95%]	*p*
1 : Benign *vs* premalignant/malignant (0–20)	11.3	10.9	[-0.5 : 1.3]	0.38
2 : Level of confidence (0–10)	5.2	5.5	[-0.8 : 0.2]	0.19
3 : Precise diagnosis (0–20)	9.3	9.5	[-1.3 : 0.8]	0.63
4 : MCQ (0–25)	19.9	20.5	[-1.3 : 0.1]	0.11

MCQ: multiple choice questionnaires

The lesions correctly identified as premalignant or malignant by more than half of the participants were Bowen’s disease (*i*.*e* intraepithelial squamous cell carcinoma), both cutaneous and mucosal SCC and typical presentation of melanoma (Table 3).

**Table 3:** Adequate identification of the premalignant/malignant nature of the skin lesions, before (Test 1) and after (Test 2) online training (or not).

Premalignant/malignant skin lesions	Online training group (n = 71)		Control group (n = 70)	
	Test 1	Test 2	Test 1	Test 2
AK 1*	17 (24%)	33 (58%)	24 (34%)	23 (34%)
AK 2	23 (32%)	19 (33%)	16 (23%)	19 (28%)
Bowen’s disease	41 (58%)	34 (60%)	38 (54%)	36 (54%)
Cutaneous SCC 1	40 (56%)	30 (53%)	38 (54%)	27 (40%)
Cutaneous SCC 2	65 (92%)	55 (96%)	63 (90%)	57 (85%)
Mucosal SCC*	46 (65%)	50 (88%)	50 (71%)	50 (75%)
BCC 1*	34 (48%)	38 (67%)	35 (50%)	32 (48%)
BCC 2*	57 (80%)	52 (91%)	61 (87%)	56 (84%)
Melanoma	66 (93%)	55 (96%)	63 (90%)	59 (88%)
Acral achromic melanoma	33 (46%)	31 (54%)	26 (37%)	20 (30%)
Cutaneous lymphoma*	2 (3%)	27 (47%)	2 (3%)	4 (6%)

AK: actinic keratosis; SCC: squamous cell carcinoma; BCC: basal cell carcinoma.

* lesions with difference before and after formation superior to 10 points in the online training group, while difference between Test 1 and Test 2 was inferior to 5 points in the control group

In contrast, the premalignant or malignant nature of actinic keratosis (AK), acral achromic melanoma and cutaneous lymphoma was not recognized by a vast majority of rheumatologists (Table 3).

The lesions adequately identified as benign by more than 50% of participants were molluscum pendulum, dermatophytosis and dermatofibroma (Table 4). Lesions wrongly identified as malignant were seborrheic keratosis (SK), comedone, ungueal hematoma, plantar wart, and epidermal cyst (Table 4).

**Table 4:** Adequate identification of the benign nature of the skin lesions, before (Test 1) and after (Test 2) online training (or not).

Benign skin lesions	Online training group (n = 71)		Control group (n = 70)	
	Test 1	Test 2	Test 1	Test 2
SK 1*	35 (49%)	36 (63%)	29 (41%)	30 (45%)
SK 2*	31 (44%)	40 (70%)	24 (34%)	29 (43%)
Dermatofibroma*	45 (63%)	48 (84%)	34 (49%)	44 (66%)
Comedone*	24 (34%)	34 (60%)	32 (46%)	26 (39%)
Ungueal Hematoma	29 (41%)	23 (40%)	18 (26%)	20 (30%)
Plantar wart*	35 (49%)	36 (63%)	21 (30%)	27 (40%)
MP	66 (93%)	56 (98%)	67 (96%)	65 (97%)
Epidermal cyst*	32 (45%)	38 (67%)	21 (30%)	35 (52%)
Dermatophytosis	68 (96%)	47 (82%)	67 (96%)	64 (96%)

SK: Seborrheic keratosis; MP: Molluscum pendulum.

* lesions with difference before and after formation superior to 10 points in the online training group, while difference between Test 1 and Test 2 was inferior to 5 points in the control group

### Impact of the online formation

Regarding the primary evaluation criterion (Score 1: diagnosis of the benign vs premalignant/malignant nature of the lesions), the means difference in the number of adequate responses at Test 2 between groups was 2.2 points, favoring the online training group (IC95%: 1.3; 3.1), with a *p* value <0.0001 (Table 5).

**Table 5:** Impact of online training (Test 2).

Scores (range)	Online training group (n = 71)	Control group (n = 70)		
	Mean score	Mean score	[CI 95%]	*p*
1 : Benign *vs* premalignant/malignant (0–20)	13.4	11.2	[1.3: 3.1]	7.6x10^-6^
2 : Level of confidence (0–10)	5.6	5.7	[-0.7 : 0.3]	0.46
3 : Precise diagnosis (0–20)	11.7	9.6	[1.1 : 3.1]	9.3x10^-5^
4 : MCQ (0–25)	21.7	20.8	[0.2: 1.7]	0.016

MCQ: multiple choice questionnaires

A significant difference at Test 2 was also found for Scores 3 and 4. Finally, the level of confidence of the rheumatologists (Score 2) was not statistically different between groups (Table 5).

We identified several lesions where the formation allowed an important better recognition of the benign or malignant nature of the tumor (difference before and after formation superior to 10 points in the trained group, while difference between Test 1 and Test 2 was inferior to 5 points in the control group): BCC, mucosal SCC, cutaneous lymphoma, AK for malignant/premalignant lesions (Table 3); SK, plantar wart, dermatofibroma, comedone, epidermal cyst for benign lesions (Table 4).

## Discussion

Our study suggests that French rheumatologists appear poorly concerned about the risk of skin cancers in patients with inflammatory rheumatism treated with cDMARDs and follow poorly the national recommendations since search for skin cancer risk, skin examination or dermatologist referral is performed by a minority of participating rheumatologists. In theory, the French Society of Rheumatology indeed recommends that any patient affected with chronic inflammatory rheumatism should be examined at least once by a dermatologist, with an annual check-up in case of past history of skin cancer, fair skin or hair, regular and repeated sun exposure, past photo-therapy, multiple nevi, immunosuppressive treatment (prednisone >20 mg/d) and past treatment with radiotherapy. In the same line, the COMORA study evaluating RA monitoring also found that an optimal screening for skin cancers, at least one examination of RA patients by a dermatologist and yearly referral if more than 40 nevi are present, was performed in only 23.9% of the patients [9]. Much more concern is given to patients receiving biotherapy but only half of the participant rheumatologists referred yearly to the dermatologist, despite the national annual check-up recommendation of the French Club Rhumatismes et Inflammation (CRI) [10].

On the contrary, a greater concern regarding smoking cessation in patients affected with chronic inflammatory rheumatism was observed in participating rheumatologists, probably not only due to the increased risk of developing lung cancer in these patients but also to the well-established impact of smoking on disease activity and response to treatment.

Because of a limited access to a dermatologist in several countries [11, 12], it appeared original and of interest to provide a dedicated formation to rheumatologists dealing with a population at increased risk of skin cancers. The baseline evaluation of rheumatologists identified a satisfactory global cognitive knowledge about skin cancers and a global good level of recognition of several benign or malignant skin lesions when presenting themselves in their typical type. In contrast, most “black tumors” were wrongly considered as malignant (SK, comedone, haematoma), the premalignant nature of AK was not recognized and cutaneous lymphoma, which represents a classical differential diagnosis for psoriasis, were completely unknown by a vast majority of participating rheumatologists.

However, rheumatologists who received the online formation obtained a better cognitive knowledge on skin tumors, were more able to identify the malignant or non-malignant nature of skin lesions and also to precisely identify the different skin lesions. Trained rheumatologists also significantly improved their baseline scores after the formation. More specifically, recognition of AK as premalignant lesions and of cutaneous lymphoma as malignant lesions was considerably improved, as well as the recognition of SK as benign lesions.

Altogether, the major strengths of our study are 1) that we have performed a rigorous assessment of this online training, using a randomized trial, which is rarely performed in the educational field; 2) this trial has included a large number and a wide pattern of participants (hospital practice, private practice or mixed practice) which guaranties an excellent external validity; 3) the primary end point is very relevant to clinical practice (malignant lesion requiring urgent referral to the dermatologist/ benign lesion not requiring referral to the dermatologist); 4) the formation to skin tumors was not dedicated to general practitioners but to rheumatologists and this training also aimed at improving knowledge regarding benign skin tumors. The limits of our study include a 34% response rate that could have selected rheumatologists basically more interested in the field of skin tumors (however, this response rate is usual in web-based surveys), and the absence of long term evaluation in order to test the maintenance of the positive impact of the formation. It would be now interesting to evaluate the impact of the formation on the rheumatologist practice regarding skin cancers in patients with inflammatory rheumatisms and to identify if the formation has modified the referral of patients to the dermatologist, allowing the diagnosis of more malignant tumors and limiting the referral for benign tumors.

## Supporting Information

S1 File Baseline test performed by the rheumatologists (Test 1). (PDF)

S1 Text Questionnaire assessing the rheumatologists habits regarding skin cancer detection. (PDF)
