# Supplementary material for: Online Training on Skin Cancer Diagnosis in Rheumatologists: Results from a Nationwide Randomized Web-Based Survey
Source: PLoS One. 2015 May 21;10(5):e0127564. doi: 10.1371/journal.pone.0127564 (PMC4440619; doi:10.1371/journal.pone.0127564)

Supporting information 2:
Baseline evaluation
Original language

QCM 1. Parmi les éléments suivants, lesquels sont des facteurs de risque de cancers cutanés pour un patient atteint de rhumatisme psoriasique?
A - Antécédent personnel de carcinome cutané.
B - Patient à la peau claire, très sensible aux coups de soleil.
C - Tabagisme chronique.
D - A fait plus de 200 séances de PUVAthérapie.
E – Antécédent familial de mélanome.

QCM 2. Quels sont les facteurs prédisposant au risque de cancers cutanés ?
A – Durée des traitements immunosuppresseurs conventionnels (ciclosporine, mycophenolate mofetil, azathioprine…).
B – Exposition à l'amiante.
C – Importance des expositions solaires passées.
D – Réalisation régulière d'ultraviolets artificiels à visée esthétique.
E – Nombre de séances de photothérapie à visée médicale.

QCM 3. Quels conseils de photoprotection donnez-vous à un patient traité par immunosuppresseurs et/ou biothérapies ?
A – Choisir préférentiellement l'ombre aux heures les plus chaudes de la journée.
B – Préférer une protection par un vêtement plutôt que par une crème solaire.
C – Demander au pharmacien un écran total pour pouvoir passer la journée à la plage.
D – Choisir le type de produit de protection solaire et l'indice de protection en fonction de la sensibilité du patient au soleil et de son lieu d'exposition.
E – Préparer sa peau au soleil par quelques ultraviolets artificiels.

QCM 4. Les situations cliniques suivantes représentent une contre-indication classique à une biothérapie par anti-TNF alpha:
A. Carcinome basocellulaire de type nodulaire du dos retiré chirurgicalement en totalité il y a 1 an.
B. Carcinome épidermoïde de la jambe avec atteinte ganglionnaire métastatique inguinale traitée chirurgicalement il y a 2 ans.
C. Antécédent de mélanome à type de mélanome de Dubreuilh chez la mère.
D. Syndrome des naevi atypiques (présence de nombreux naevi sur l'ensemble du tégument, de taille et de forme variables).
E. Mélanome de type nodulaire, niveau IV de Clark, 2.3 mm de Breslow de la jambe, retiré en totalité avec marges de sécurité de 2 cm il y a 6 ans.

QCM 5. Quelles sont les propositions vraies:
A. Le carcinome baso-cellulaire est une tumeur cutanée fréquente de bon pronostic.
B. Le carcinome épidermoïde (ou spino-cellulaire) ne métastase tout au plus qu'au niveau ganglionnaire loco-régional.
C. La grande majorité des mélanomes traitée chirurgicalement de façon précoce est définitivement guérie, sans évolution à distance.
D. La survie après diagnostic d'un mélanome métastatique au niveau viscéral est généralement comprise entre 6 à 9 mois.
E. Les carcinomes épidermoïdes (ou spinocellulaires) développés sur les muqueuses sont de plus mauvais pronostic que ceux développés sur la peau.

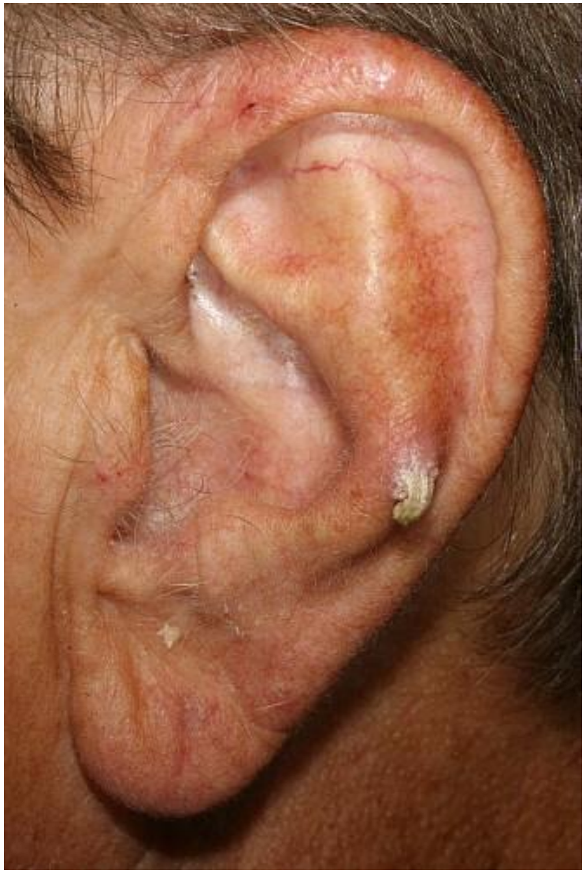
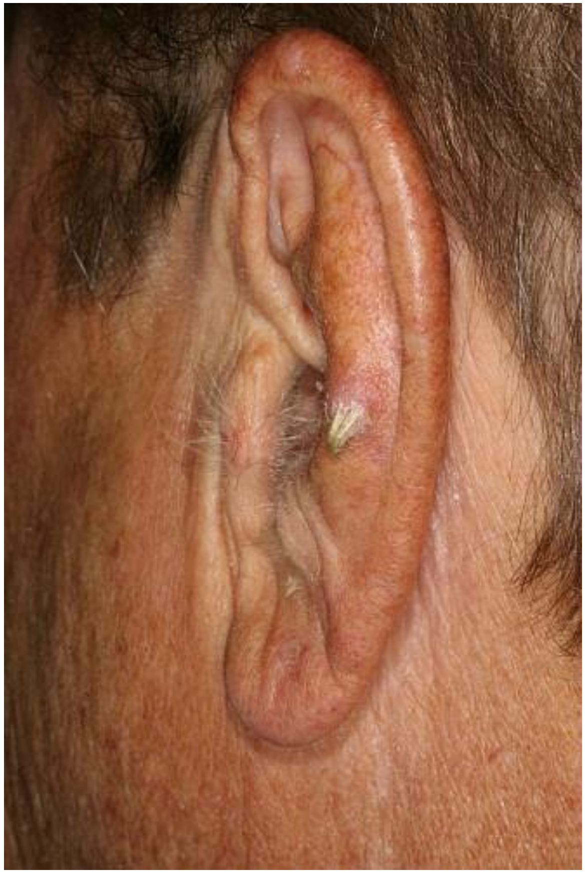

Lésion de l'oreille, apparue il y a plus d'un an chez un homme de 70 ans aux ATCD de carcinome basocellulaire du visage. Augmentation lente de taille. Ferme au toucher. Pas de notion de saignement. Indolore.

A. Bénin
B. Pré-cancéreux ou malin

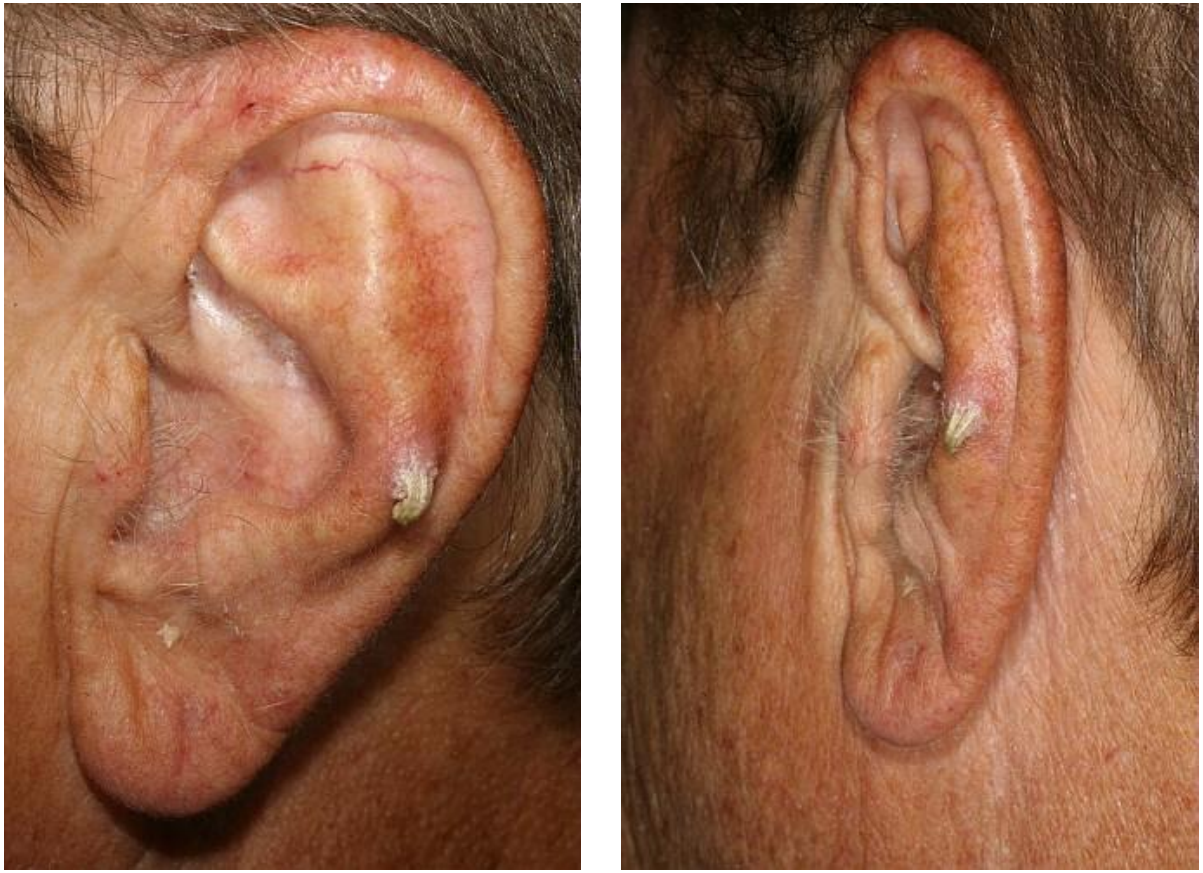

Lésion de l’oreille, apparue il y a plus d’un an chez un homme de 70 ans aux ATCD de carcinome basocellulaire du visage. Augmentation lente de taille. Ferme au toucher. Pas de notion de saignement. Indolore.

<u>S’agit-il de :</u>

A. Molluscum pendulum
B. Verrue ou papillome viral
C. Kératose actinique
D. Carcinome épidermoïde
E. Kyste épidermique ou kyste sébacé

Femme, 42 ans. Psoriasis traité avec succès par etanercept depuis 1 an, après échec photothérapie et méthotrexate.
Lésion de l'avant bras depuis 3 mois (retour de Madagascar).
Asymptomatique mais extension progressive.

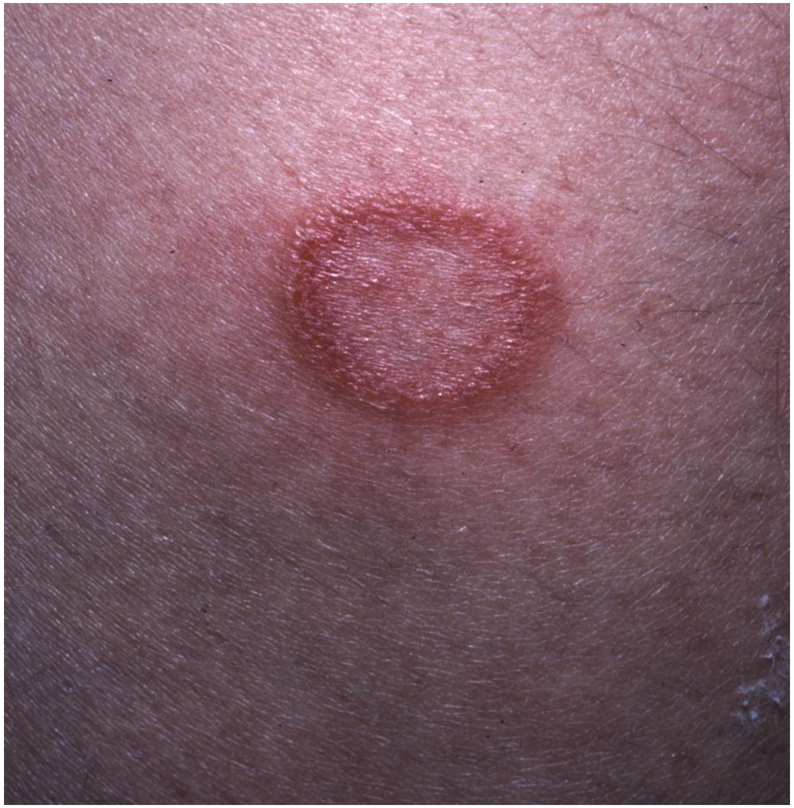

A. Bénin
B. Pré-cancéreux ou malin

Femme, 42 ans. Psoriasis traité avec succès par etanercept depuis 1 an, après échec photothérapie et méthotrexate.
Lésion de l'avant bras depuis 3 mois (retour de Madagascar).
Asymptomatique mais extension progressive.

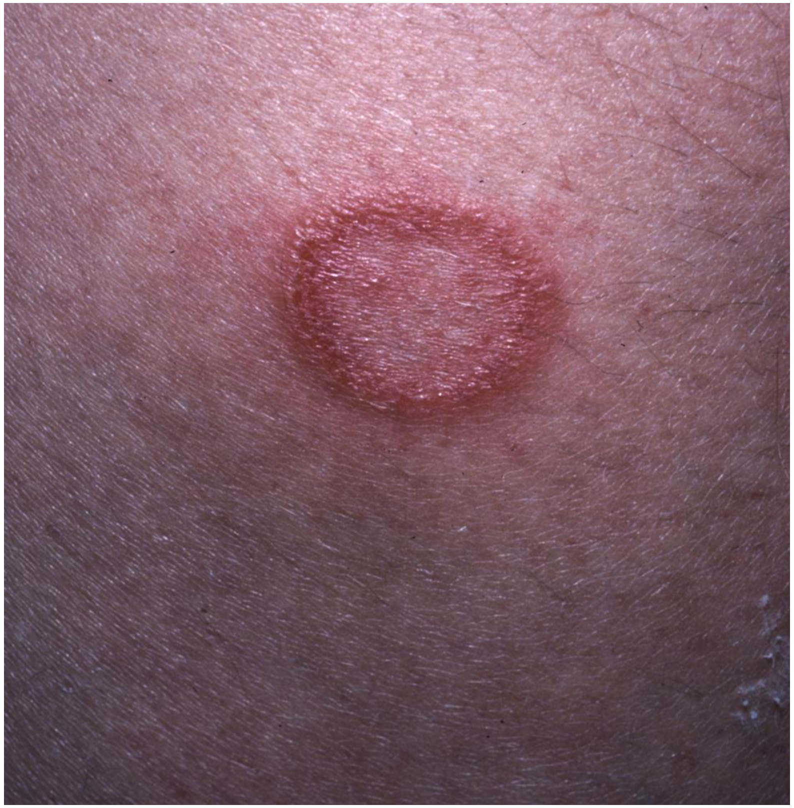

S'agit-il de:
A- Psoriasis
B- Maladie de Bowen (carcinome épidermoïde *in situ*)
C- Dermatophytie
D- Carcinome basocellulaire, dans sa forme superficielle
E- Lymphome cutané

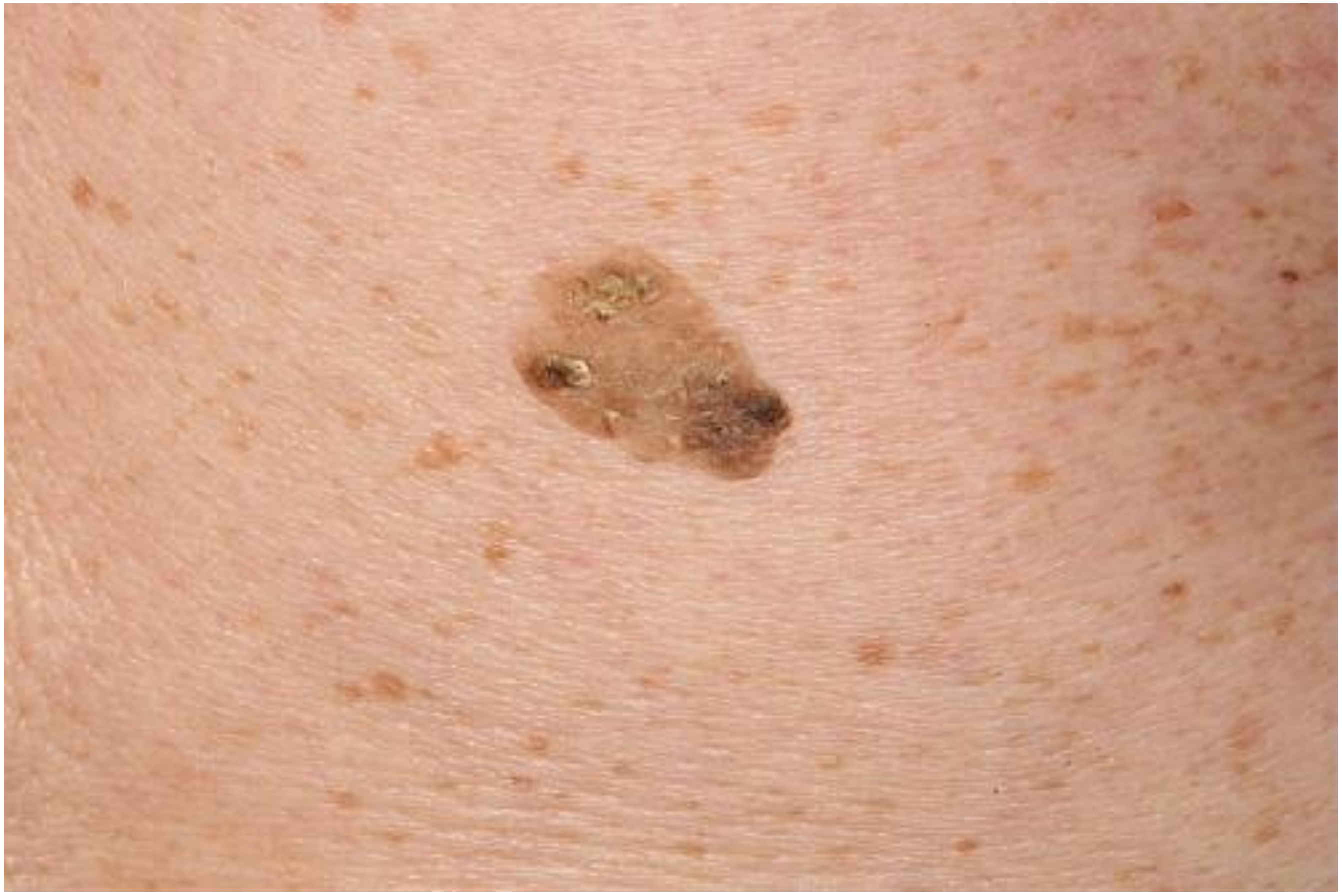

Lésion du dos chez une femme de 55 ans aux ATCD de mélanome.
Ancienneté et évolutivité non précisables par la patiente.

A. Bénin
B. Pré cancéreux ou malin

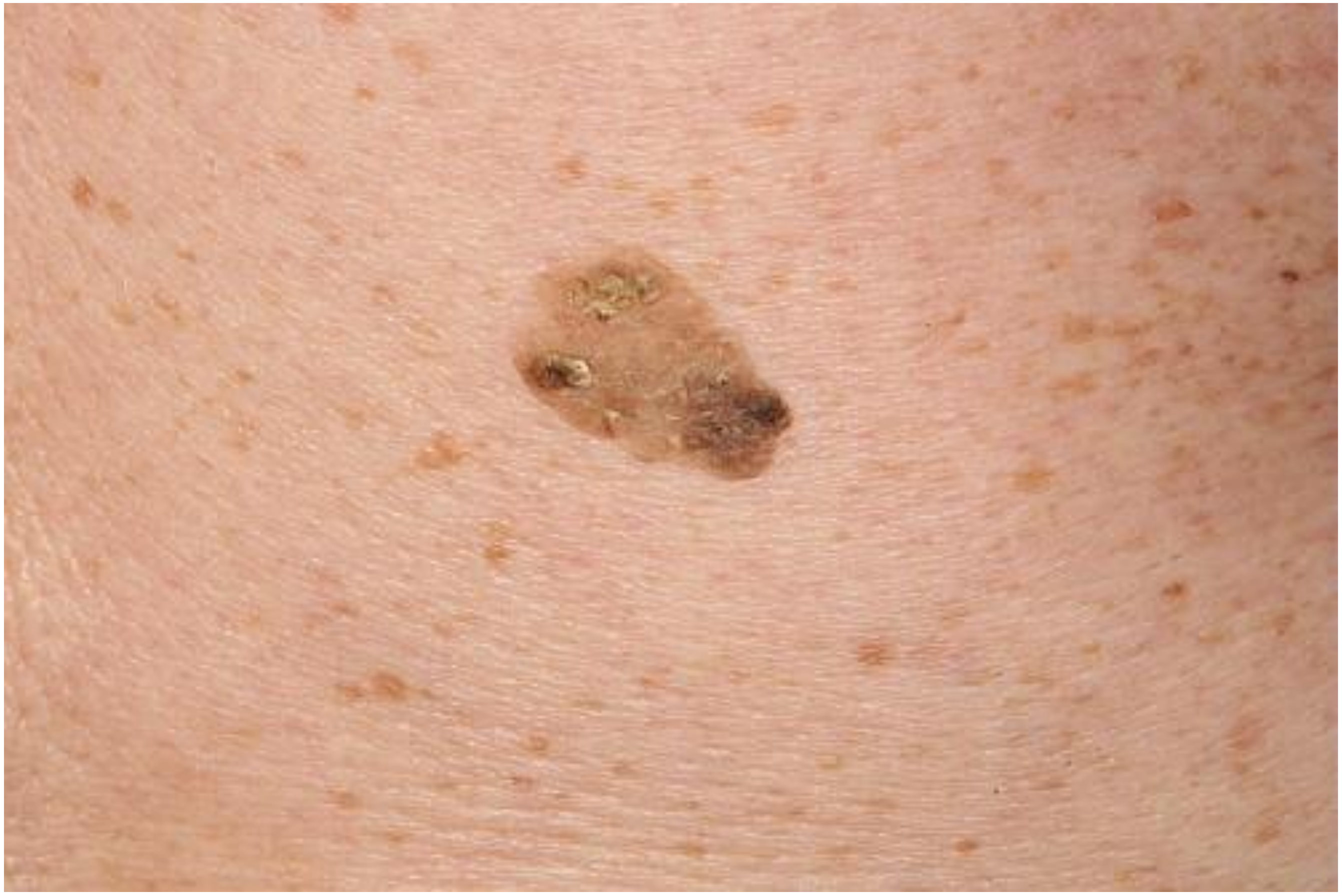

Lésion du dos chez une femme de 55 ans aux ATCD de mélanome.
Ancienneté et évolutivité non précisables par la patiente.

S'agit-il de:

A. Kératose ou verrue séborrhéique
B. Mélanome
C. Maladie de Bowen (carcinome épidermoïde in situ)
D. Naevus (« Grain de beauté »)
E. Carcinome basocellulaire

Lésion de la plante du pied (base 5ème orteil), douloureuse à l'appui, chez une femme de 55 ans aux ATCD de mélanome rétro auriculaire, présente depuis plusieurs mois, sans tendance à la cicatrisation.

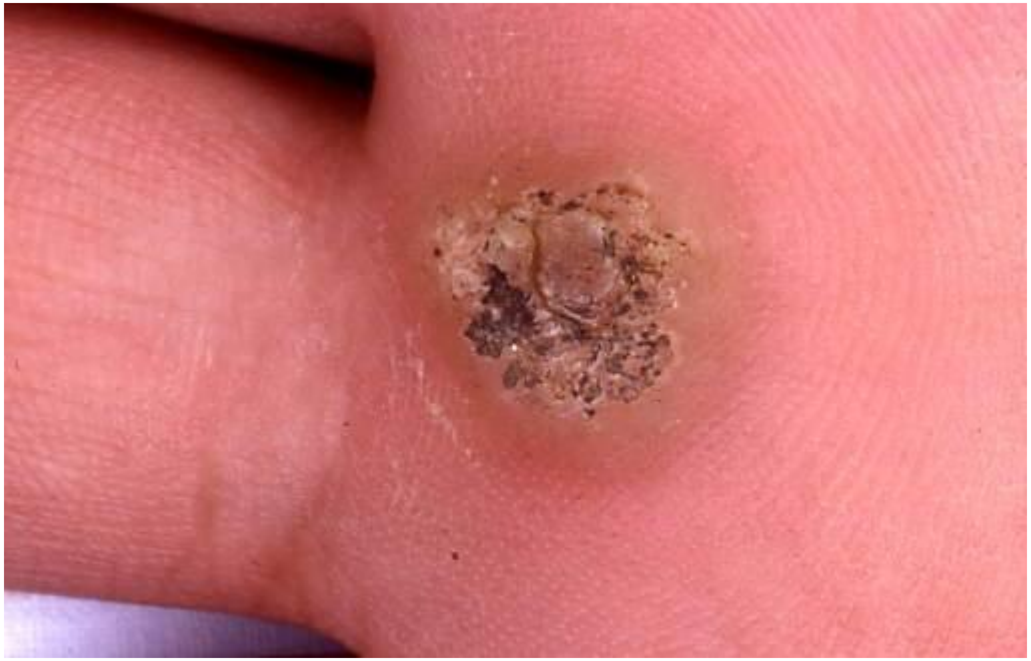

A. Bénin
B. Pré-cancéreux ou malin

Lésion de la plante du pied (base 5ème orteil), douloureuse à l'appui, chez une femme de 55 ans aux ATCD de mélanome rétro auriculaire, présente depuis plusieurs mois, sans tendance à la cicatrisation.

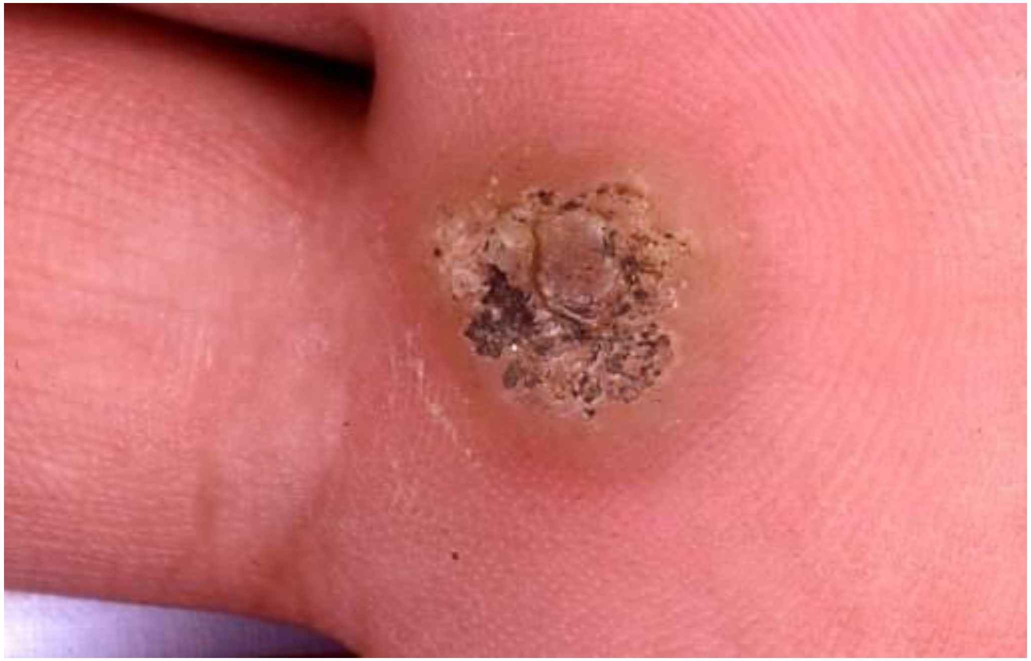

S'agit-il de :

A. Verrue plantaire
B. Mélanome
C. Carcinome épidermoïde
D. Cors/Callosité/Durillon
E. Maladie de Kaposi

Femme, 75 ans. Psoriasis traité par photothérapie pendant 20 ans.
Lésion malaire asymptomatique, augmentant progressivement de taille.

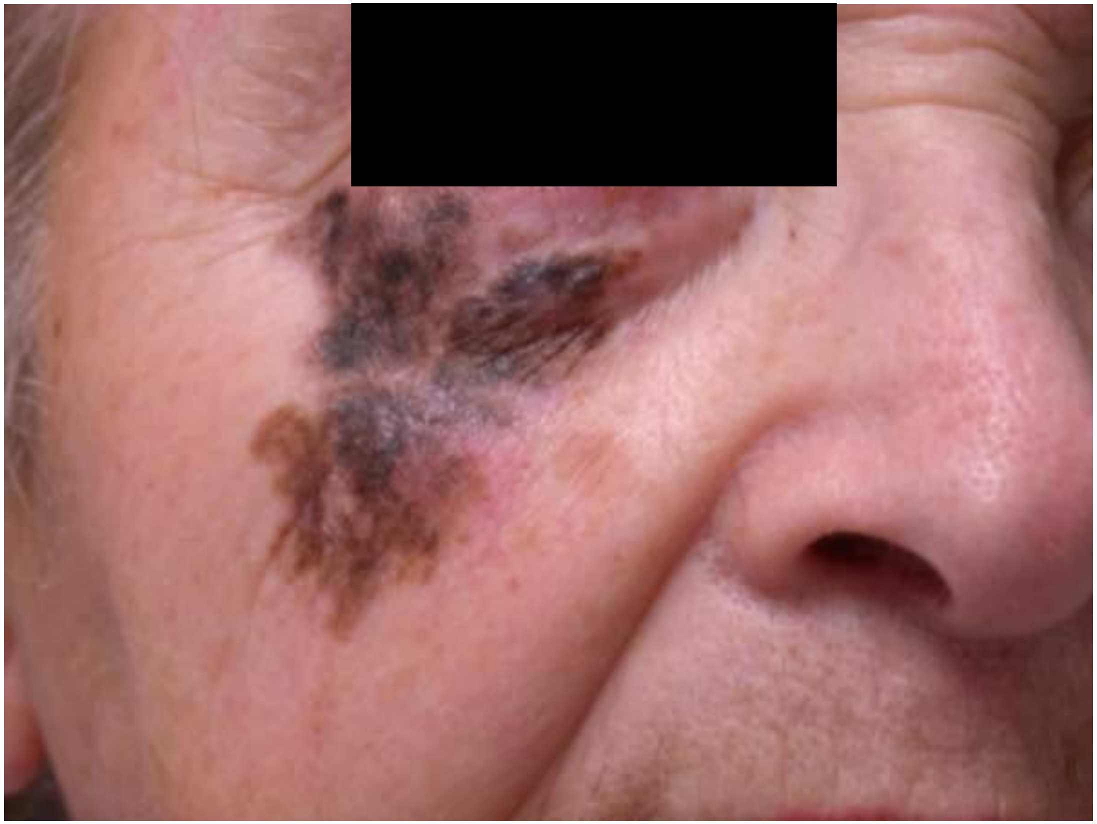

A. Bénin
B. Pré-cancéreux ou malin

Femme, 75 ans. Psoriasis traité par photothérapie pendant 20 ans.
Lésion malaire asymptomatique, augmentant progressivement de taille.

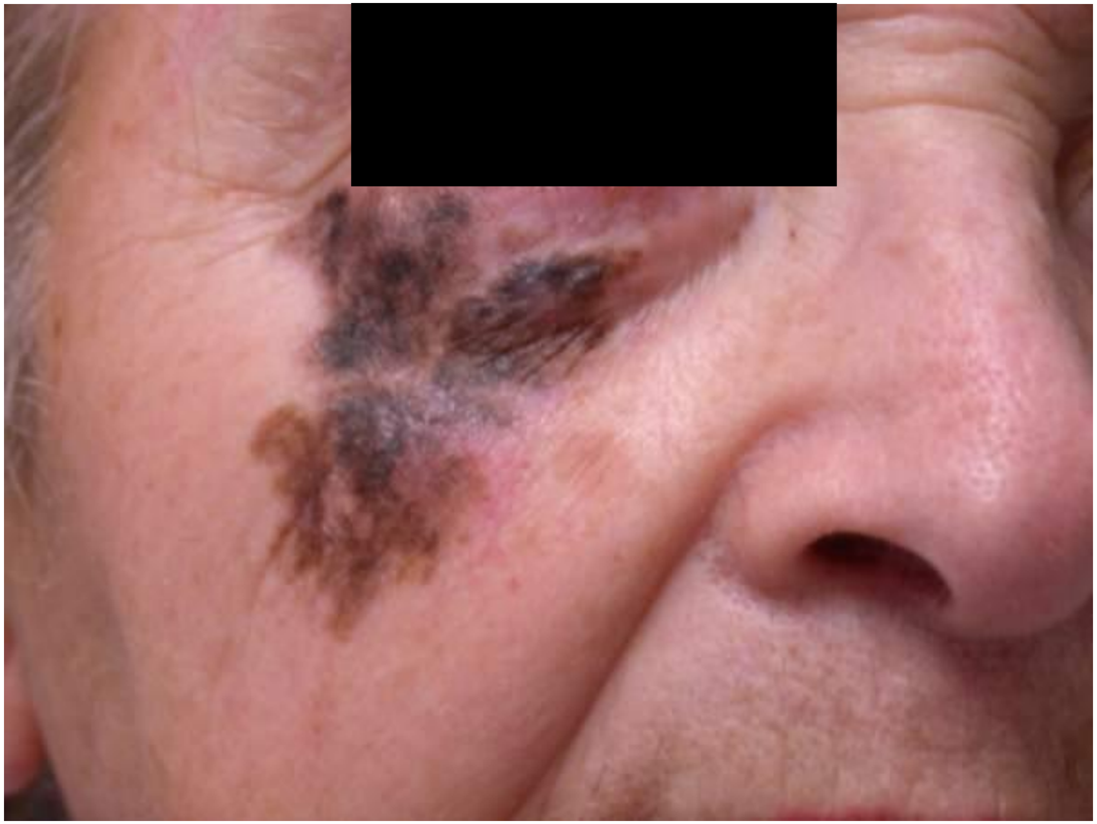

S’agit-il de:

A- Naevus naevo-cellulaire
(« Grain de beauté »)

B- Mélanome

C- Lentigo sénile (« Tâche de vieillesse »)

D- Carcinome basocellulaire pigmenté

E- Hématome post traumatique

Homme, 40 ans. Psoriasis traité par photothérapie puis méthotrexate.
Lésion angle interne œil depuis 6 mois, asymptomatique, unilatérale.
Porte des lunettes.

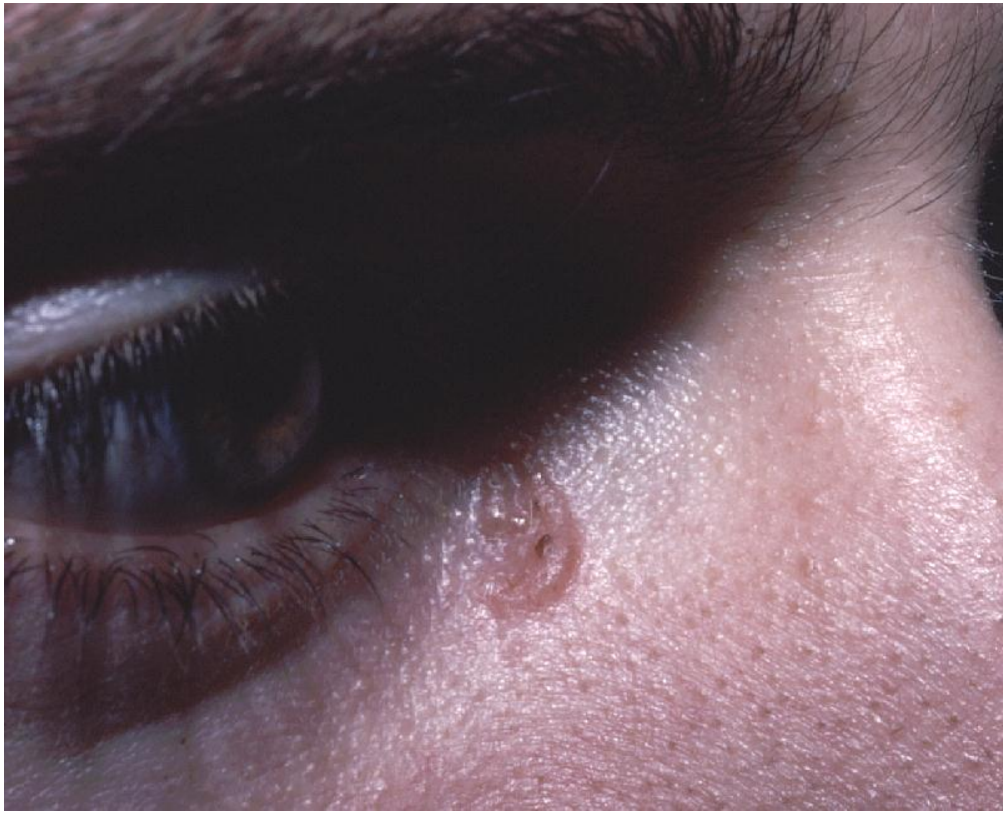

A. Bénin
B. Pré-cancéreux ou malin

Homme, 40 ans. Psoriasis traité par photothérapie puis méthotrexate.
Lésion angle interne œil depuis 6 mois, asymptomatique, unilatérale.
Porte des lunettes.

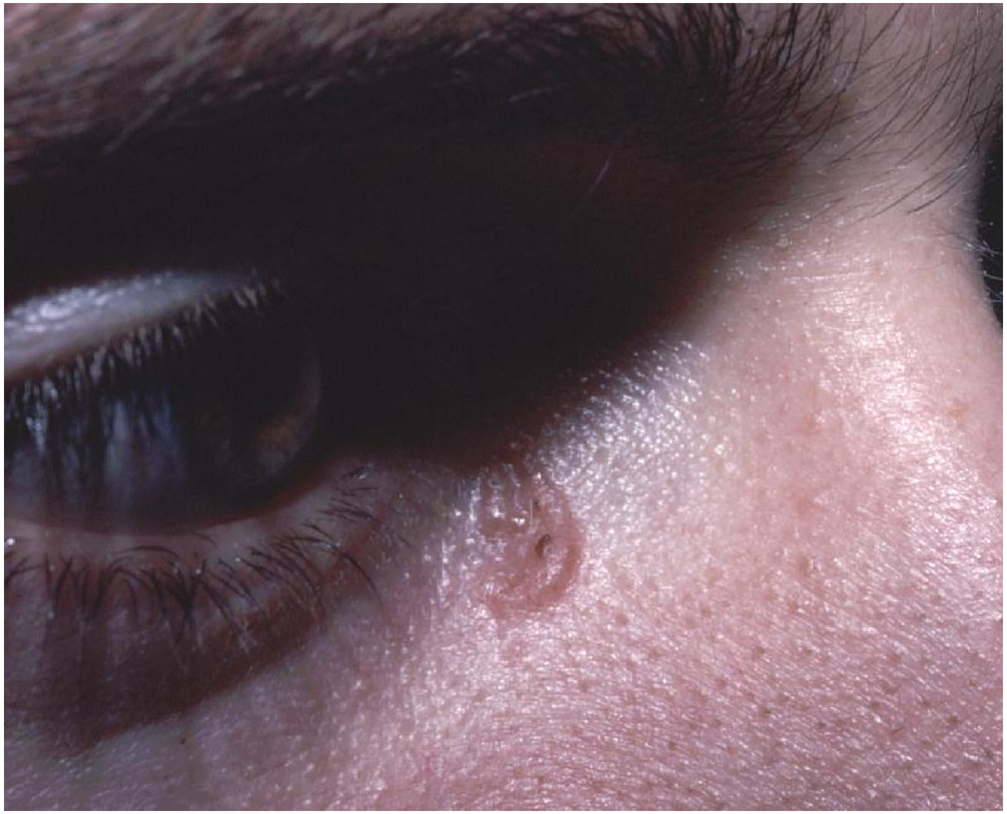

<u>S'agit-il de:</u>

A. Carcinome basocellulaire
B. Mélanome
C. Xanthélasma (dépôt cutané lipidique)
D. Kératose actinique
E. Lésion traumatique liée aux lunettes

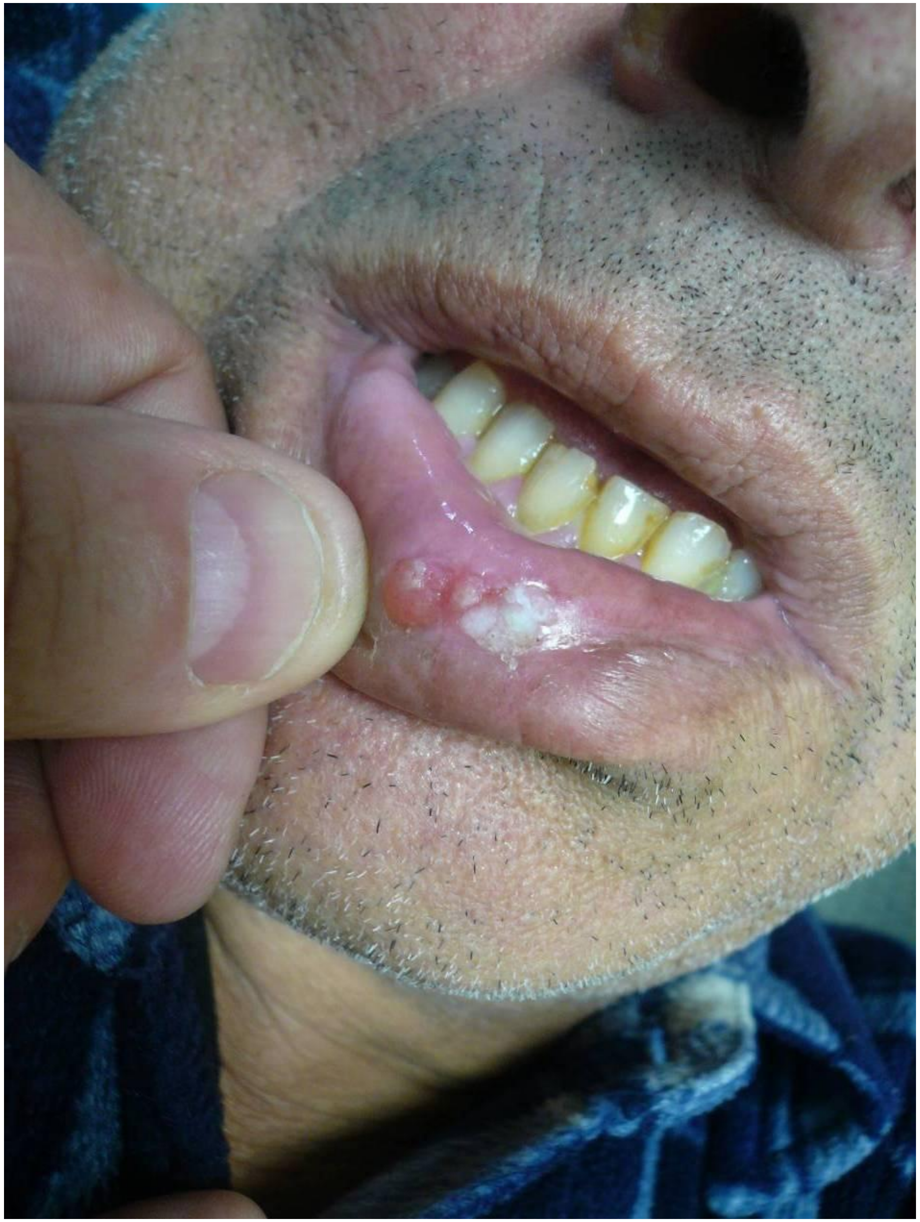

Homme, 48 ans. Tabagique.
PR sous corticoïdes et méthotrexate depuis 12 ans. Lésion de la lèvre inférieure depuis 3 mois, asymptômatique. Aires ganglionnaires libres.

A. Bénin
B. Pré-cancéreux ou malin

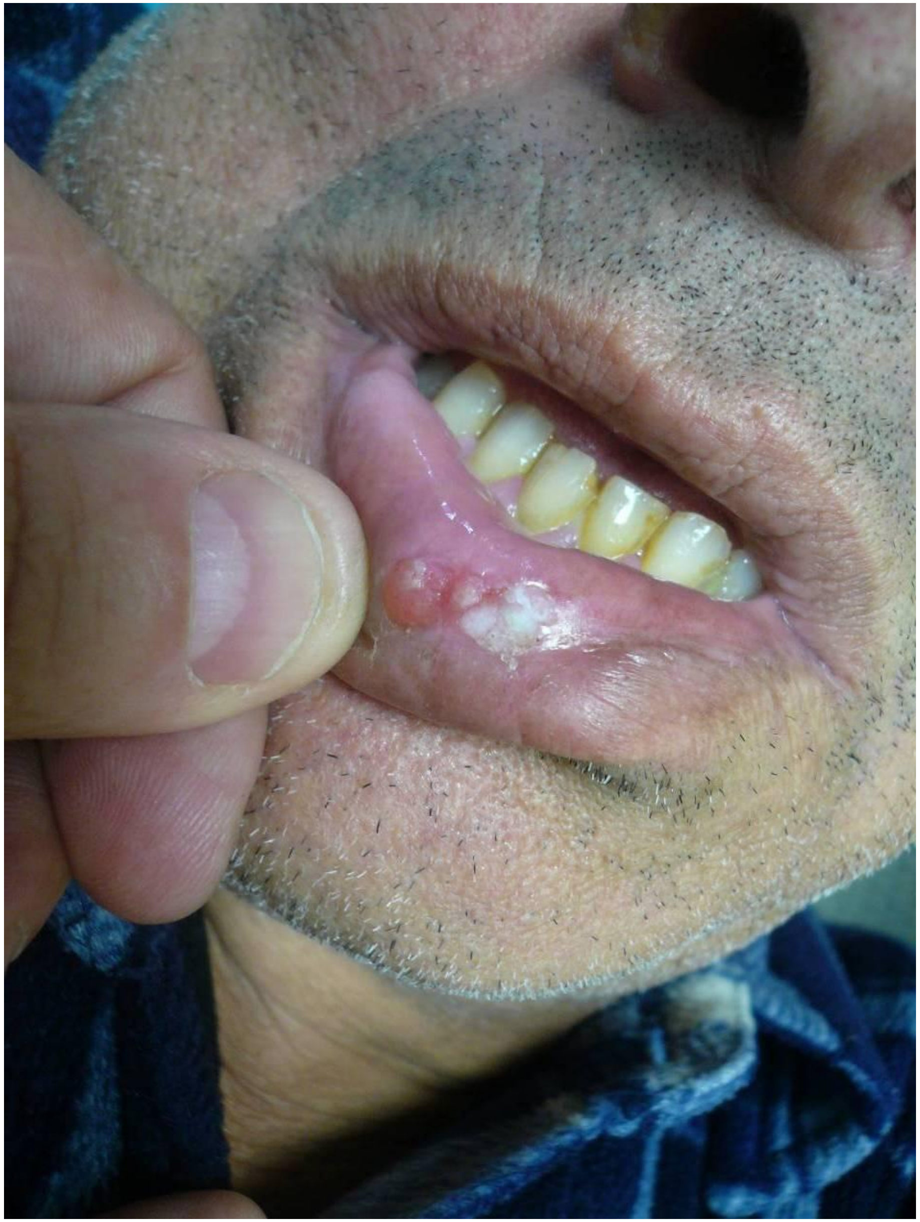

Homme, 48 ans. Tabagique.
PR sous corticoïdes et méthotrexate depuis 12 ans. Lésion de la lèvre inférieure depuis 3 mois, asymptômatique. Aires ganglionnaires libres.

S’agit-il de:

A- Aphte
B- Diapneusis (lésion post tic de mordillement)
C- Carcinome épidermoïde
D- Herpès chronique
E- Mélanome achromique

Femme 47 ans, suivie pour PR traitée par etanercept depuis 2 ans.
Sollicite un rendez vous en dermatologie en urgence pour une lésion pigmentée du sein qu'elle n'avait pas remarquée auparavant.

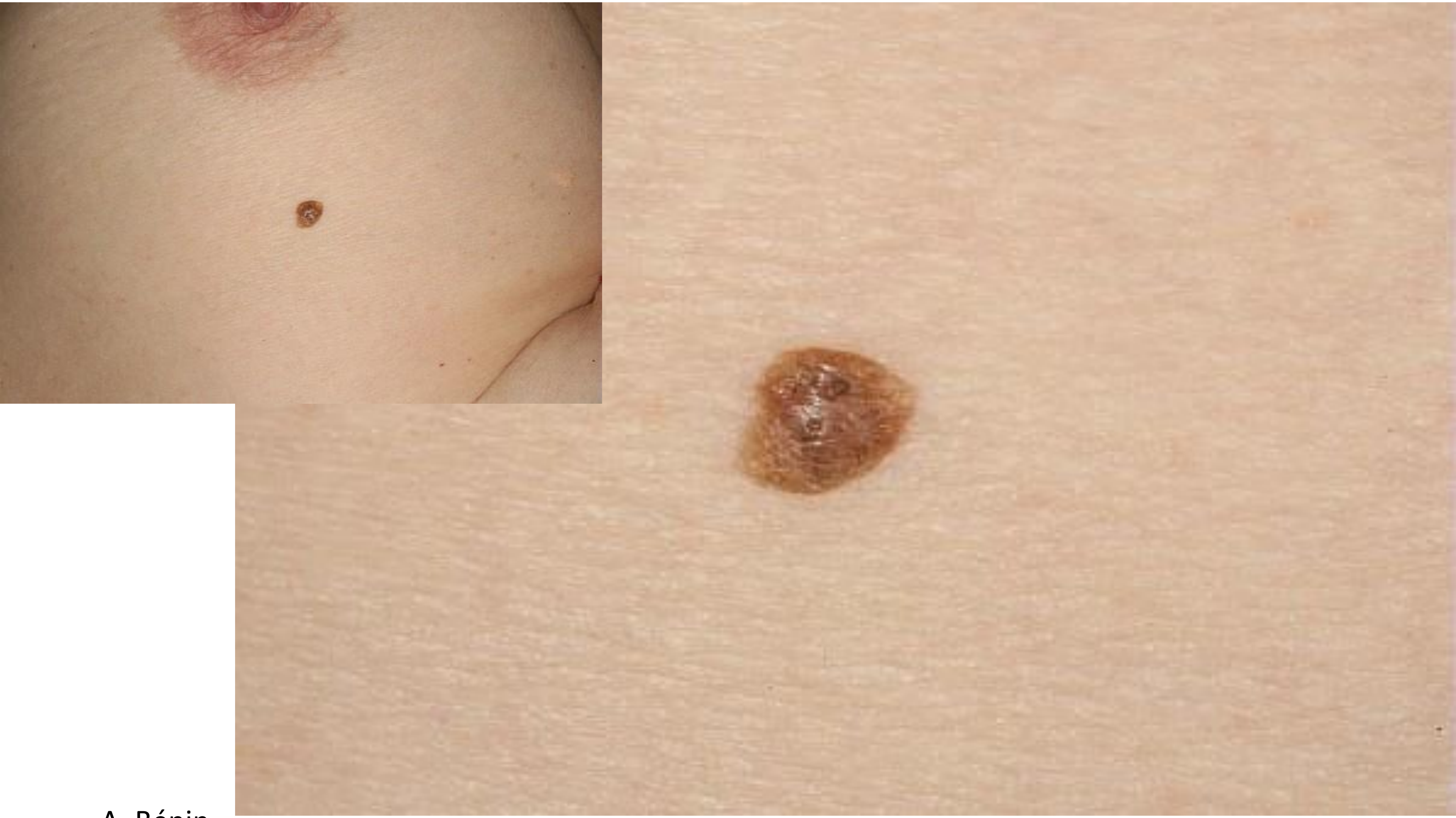

A. Bénin
B. Pré-cancéreux ou malin

Femme 47 ans, suivie pour PR traitée par etanercept depuis 2 ans.
Sollicite un rendez vous en dermatologie en urgence pour une lésion pigmentée du sein qu'elle n'avait pas remarquée auparavant.

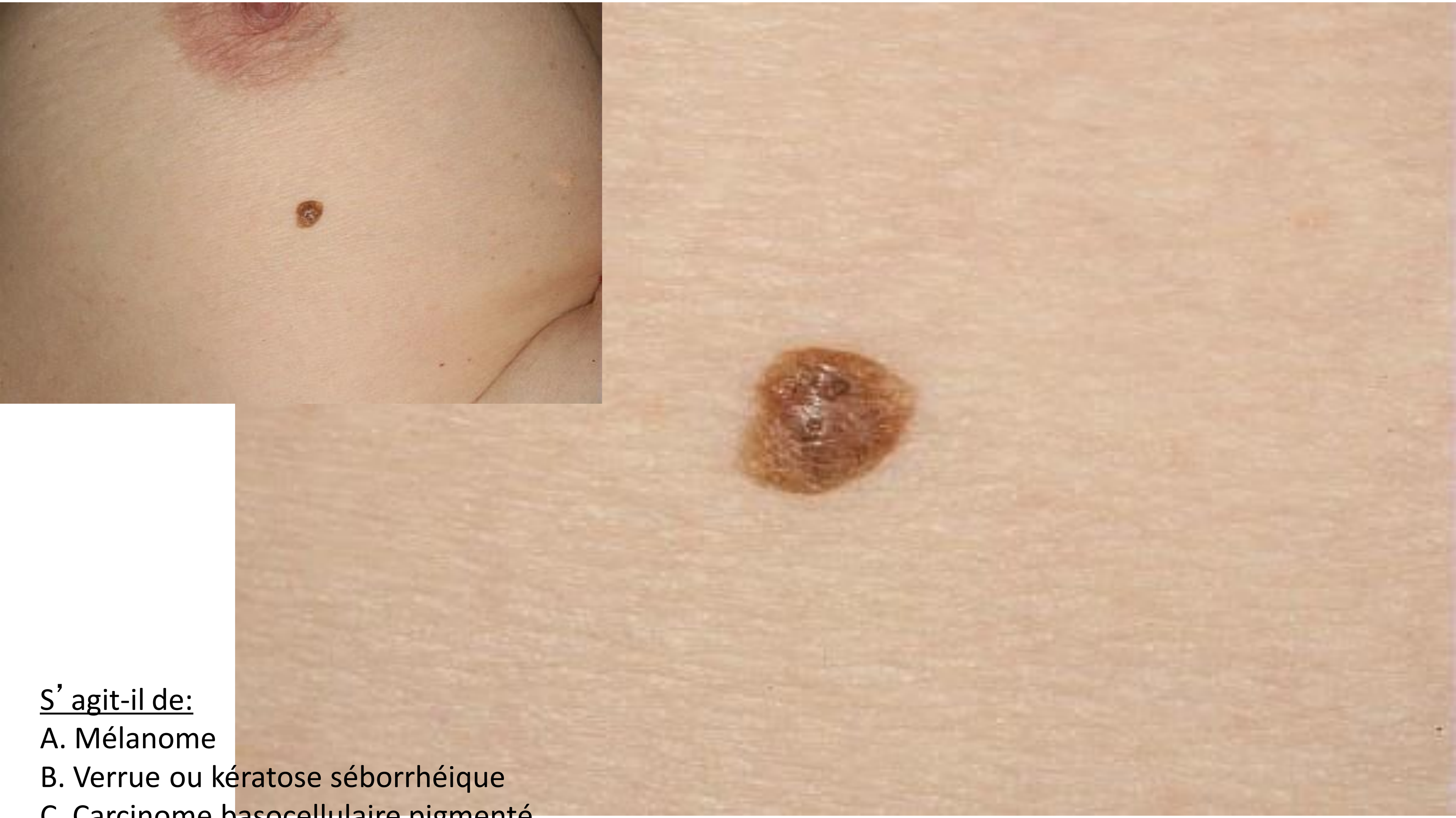

<u>S'agit-il de:</u>

A. Mélanome
B. Verrue ou kératose séborrhéique
C. Carcinome basocellulaire pigmenté
D. Naevus naevo-cellulaire (« grain de beauté »)
E. Naevus dermique (« grain de beauté en relief »)

Homme 80 ans , suivi pour mélanome latéro-thoracique droit avec atteinte ganglionnaire, en rémission depuis 5 ans.
Mise en évidence de cette lésion du dos, en regard de l'omoplate gauche, asymptômatique, de consistance molle, lors de l'examen systématique.

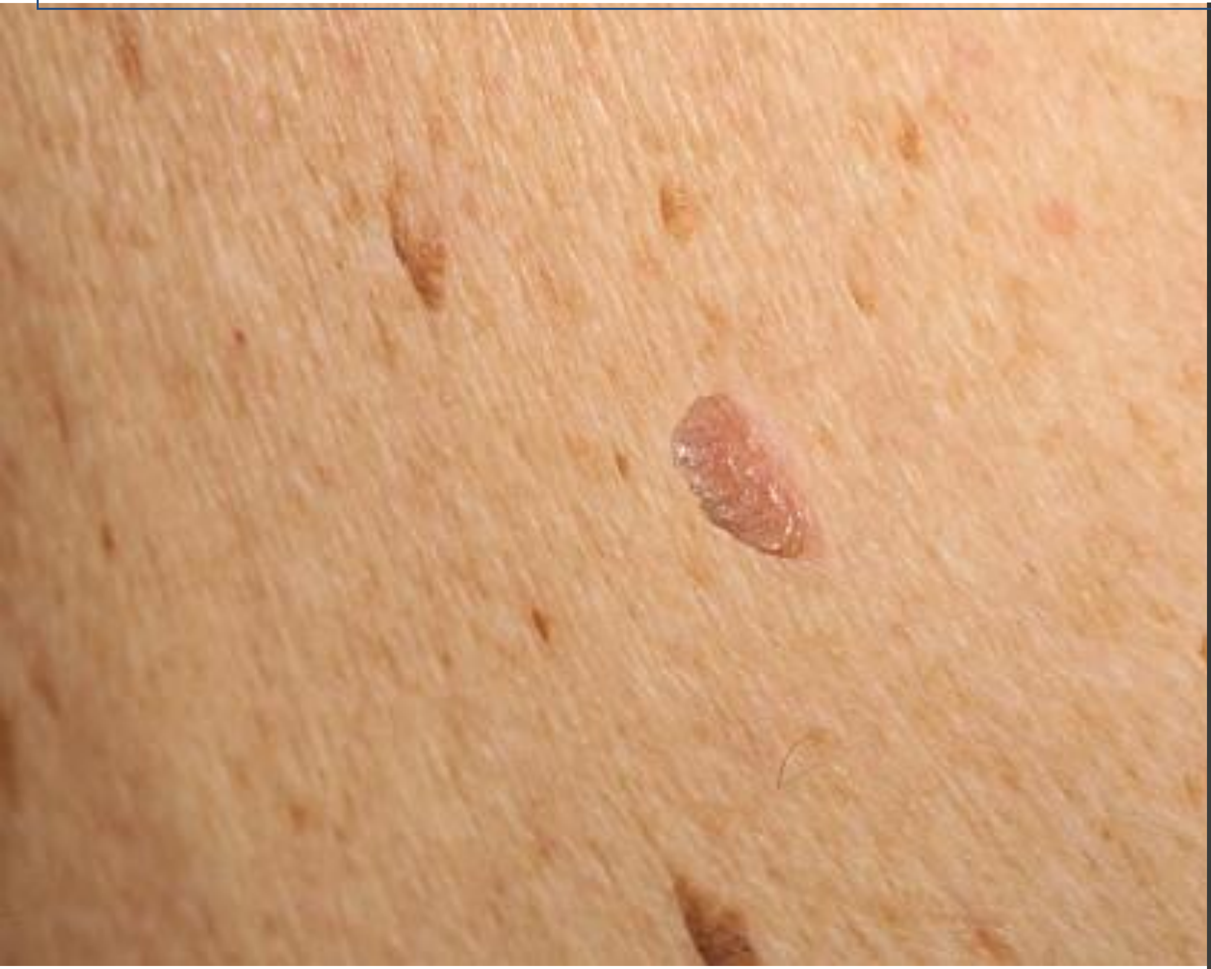

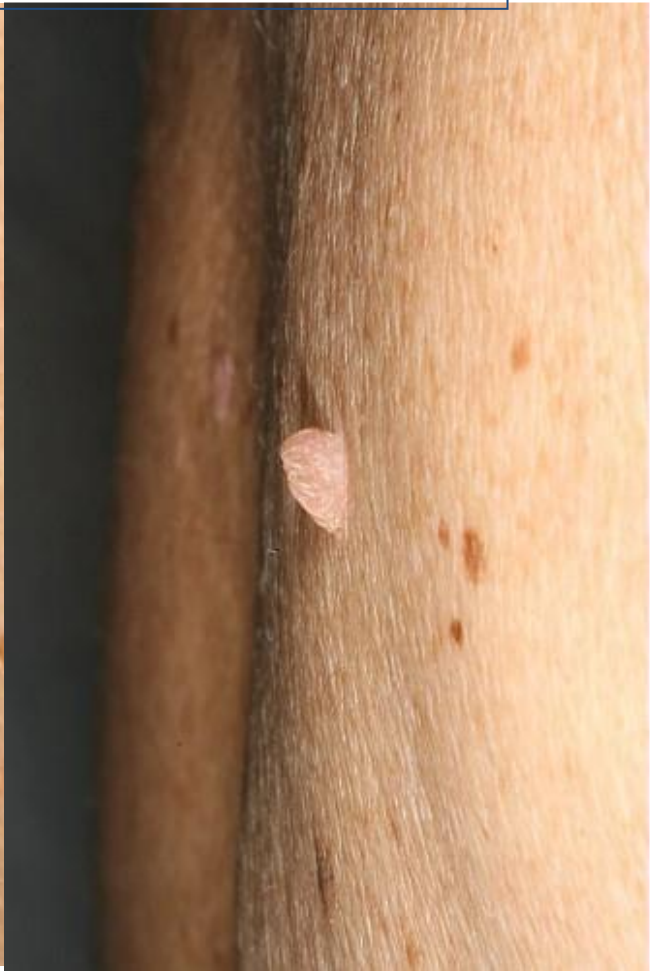

A. Bénin
B. Pré-cancéreux ou malin

Homme 80 ans , suivi pour mélanome latéro-thoracique droit avec atteinte ganglionnaire, en rémission depuis 5 ans.
Mise en évidence de cette lésion du dos, en regard de l' omoplate gauche, asymptômatique, de consistance molle, lors de l' examen systématique.

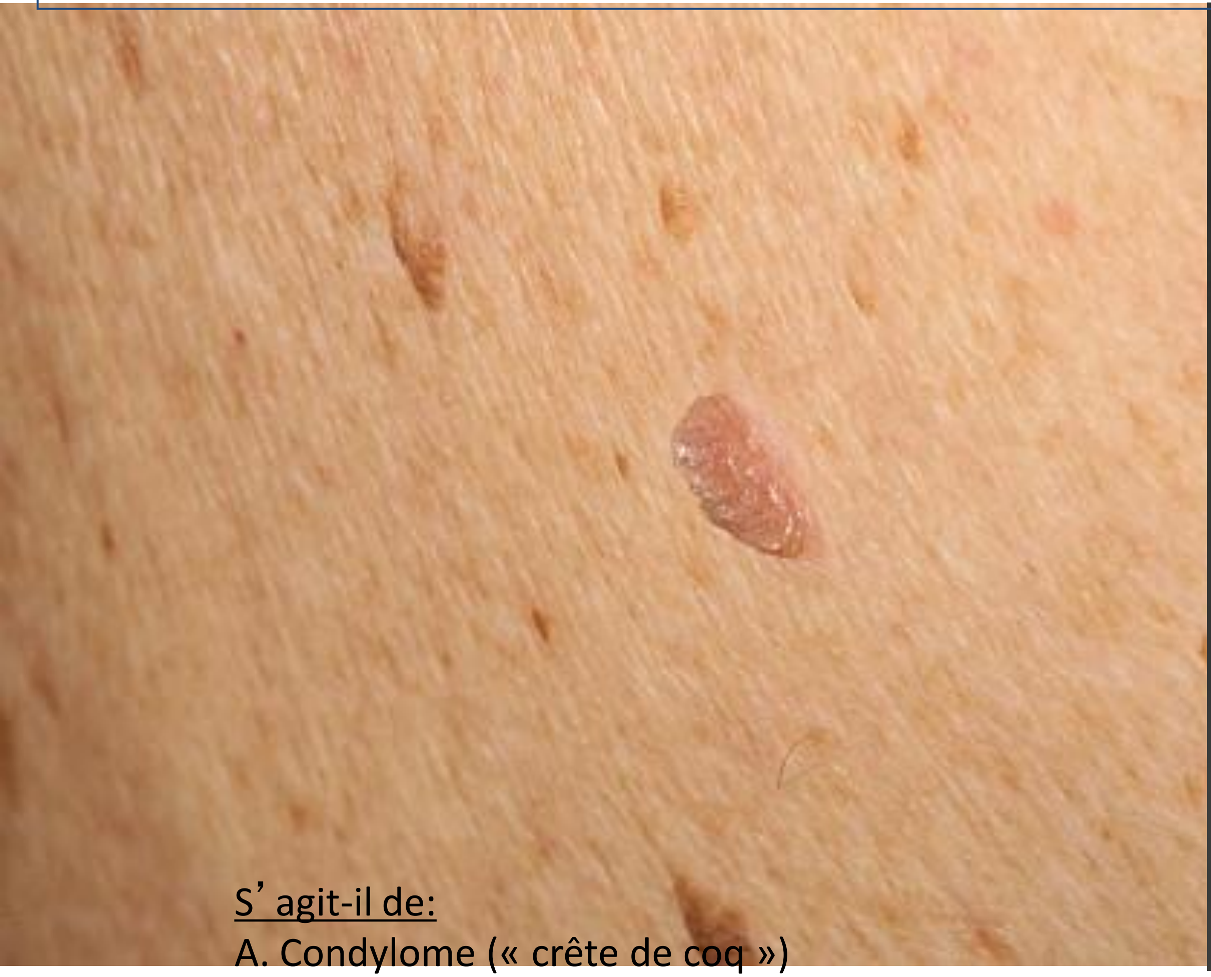

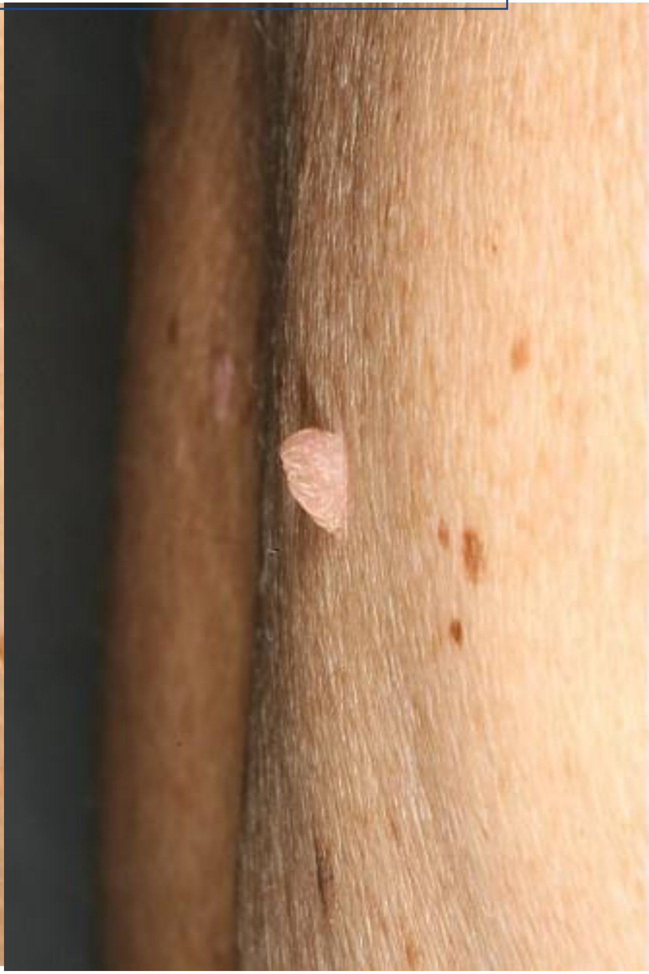

S' agit-il de:

A. Condylome (« crête de coq »)
B. Verrue (ou kératose) séborrhéique
C. Molluscum pendulum (ou achrocordon)
D. Kératose actinique
E. Naevus dermique

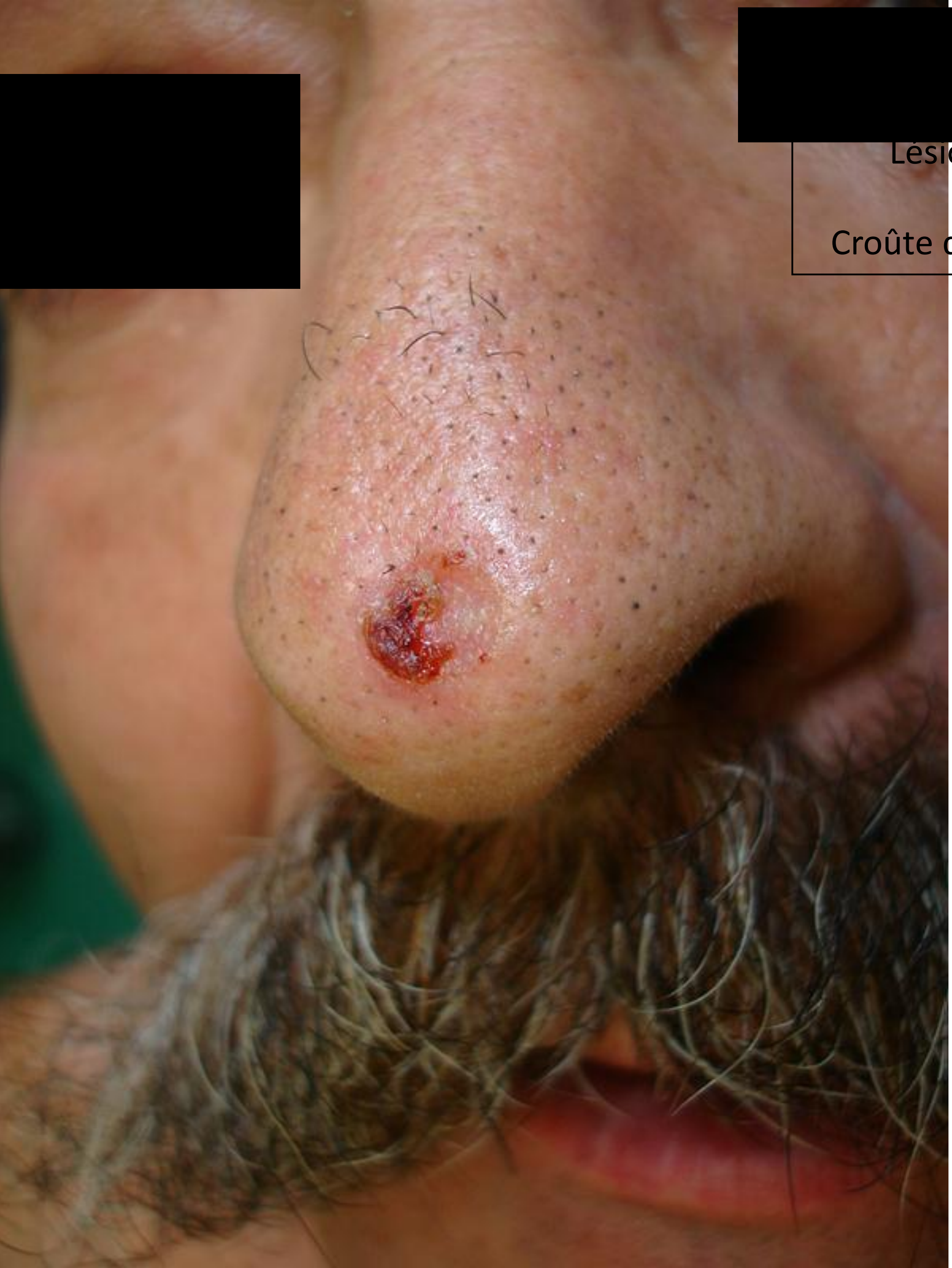

Homme, 48 ans.
Diabète insulino-dépendant.
Lésion pointe du nez depuis environ 12 mois.
Non douloureuse, non prurigineuse.
Croûte qu'il arrache, saignement et nouvelle croûte.

A. Bénin
B. Pré-cancéreux ou malin

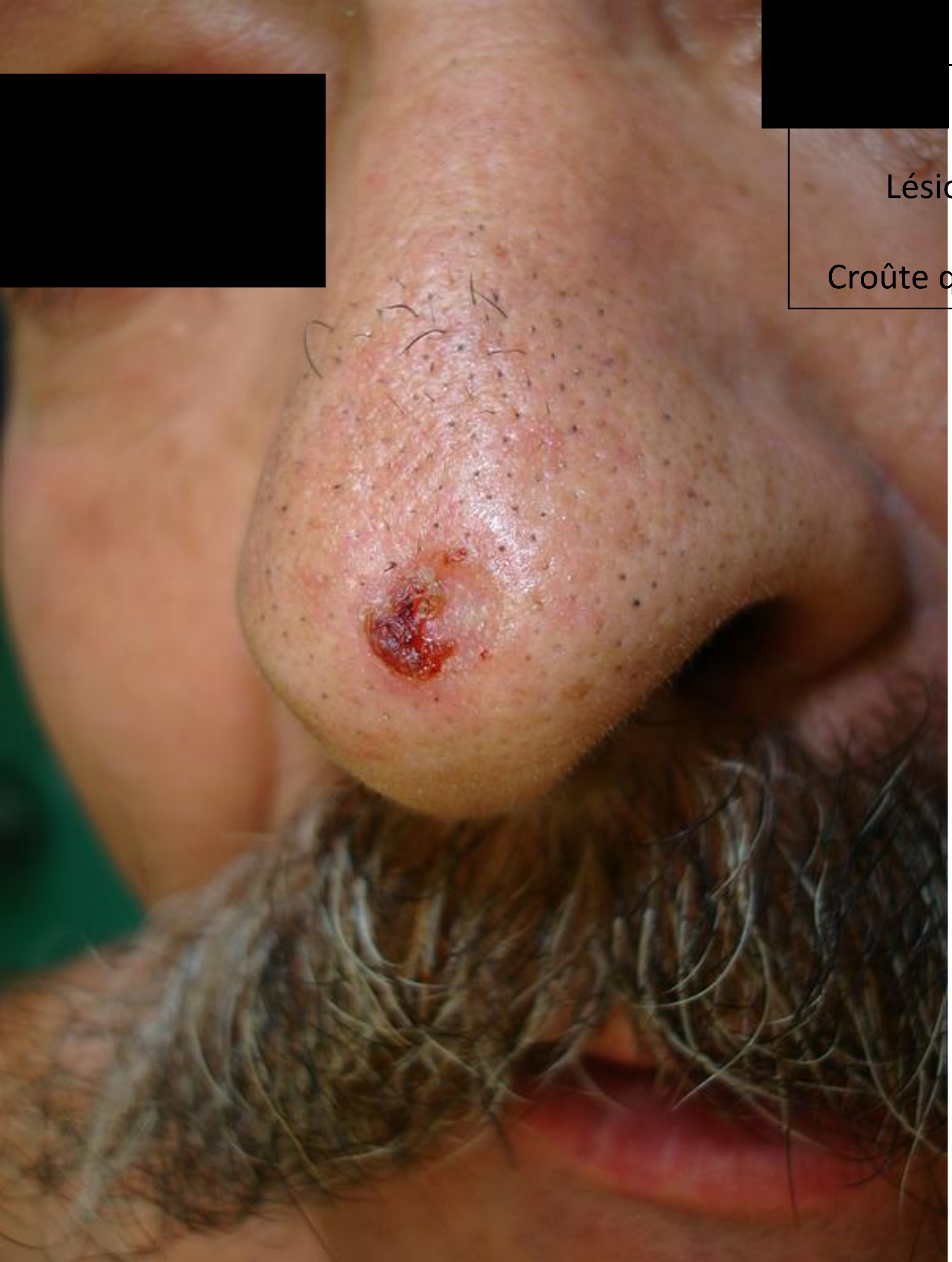

Homme, 48 ans.
Diabète insulino-dépendant.
Lésion pointe du nez depuis environ 12 mois.
Non douloureuse, non prurigineuse.
Croûte qu'il arrache, saignement et nouvelle croûte.

S'agit-il de:

A- Kératose actinique
B- Carcinome épidermoïde
C- Leishmaniose
D- Pathomimie
E- Herpès chronique

F, 35 ans. Psoriasis traité par photothérapie depuis 12 ans
Lésion lèvre supérieure depuis 6 mois. Asymptomatique

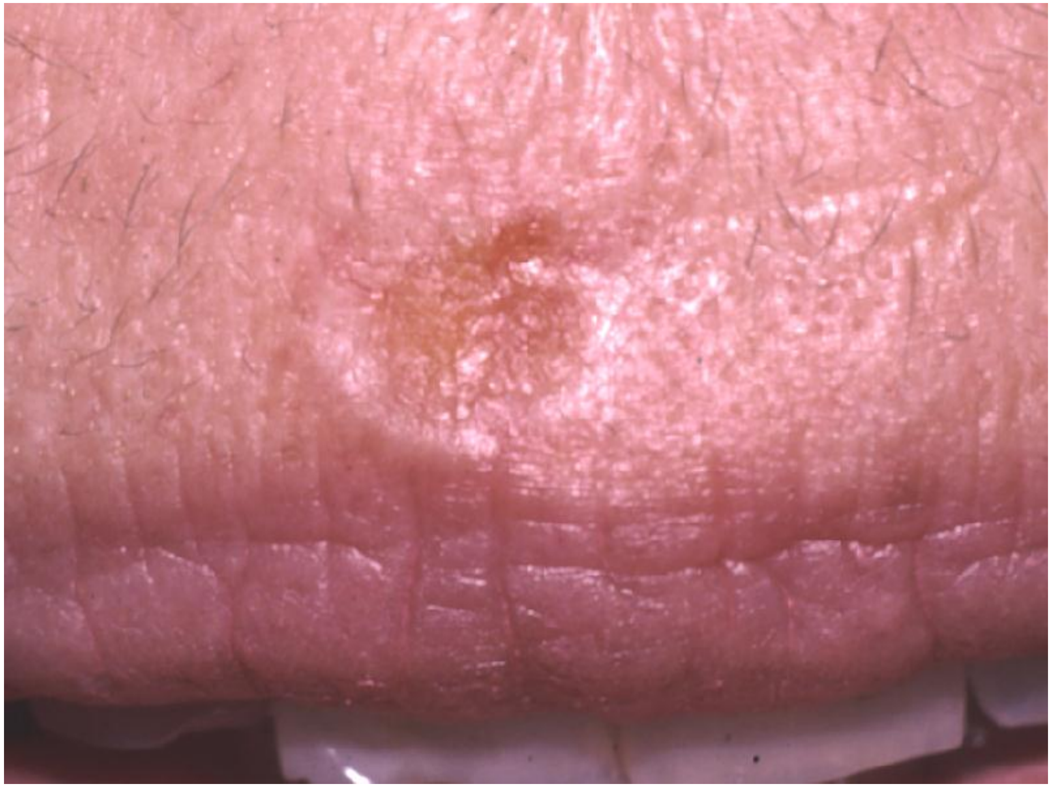

A. Bénin
B. Pré-cancéreux ou malin

F, 35 ans. Psoriasis traité par photothérapie depuis 12 ans
Lésion lèvre supérieure depuis 6 mois. Asymptomatique

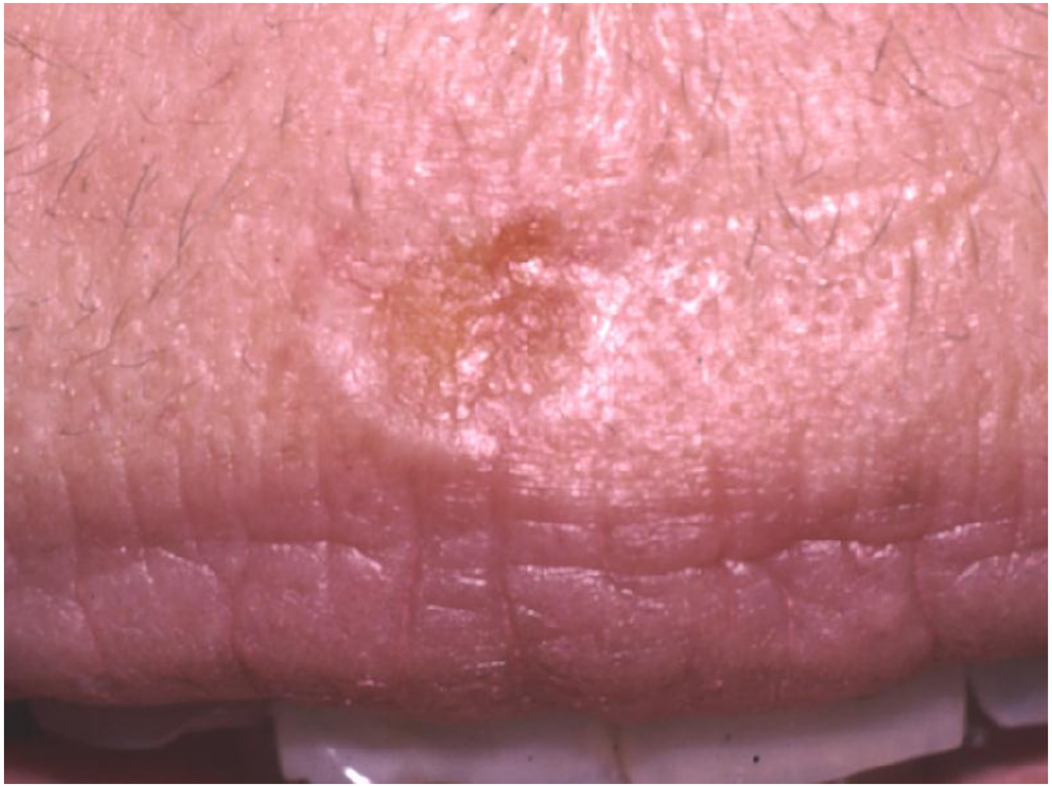

S' agit-il de:

A- Cicatrice à type de chéloïde spontanée
B- Carcinome basocellulaire
C- Dermatophytie (infection mycosique)
D- Granulome annulaire
E- Lésion secondaire à un tic de léchage

Modification rapide d' une lésion sous-cutanée du dos, jusque là de petite taille, ferme à la palpation et indolore chez un homme de 50 ans.
Augmentation de taille, apparition d' un caractère douloureux, consistance devenue molle. Le patient signale appliquer de l'éosine sur la lésion.

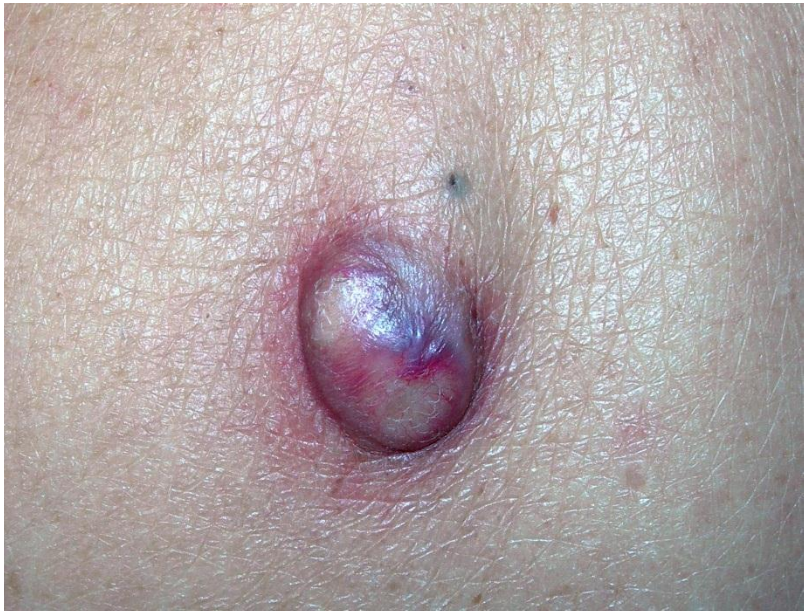

A. Bénin
B. Pré-cancéreux ou malin

Modification rapide d’une lésion sous-cutanée du dos, jusque là de petite taille, ferme à la palpation et indolore chez un homme de 50 ans.
Augmentation de taille, apparition d’un caractère douloureux, consistance devenue molle. Le patient signale appliquer de l’éosine sur la lésion.

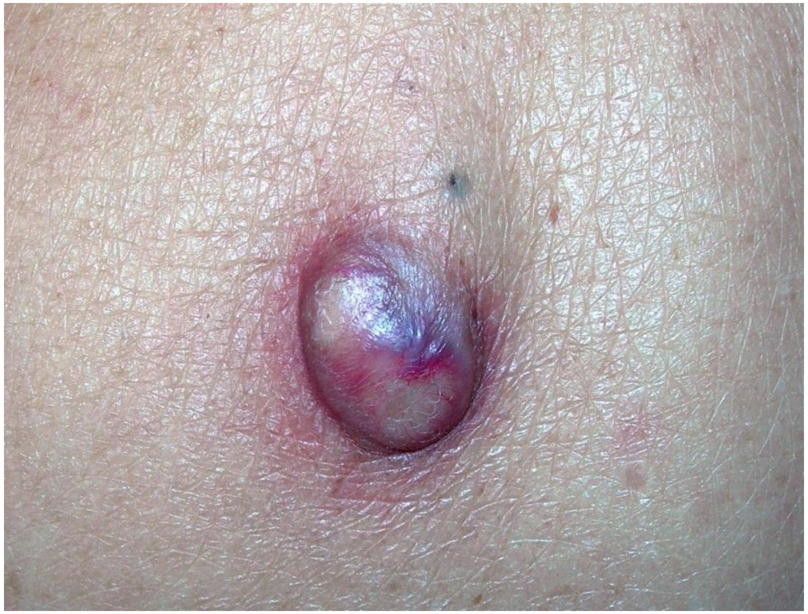

<u>S’agit-il de:</u>

A. Lipome
B. Liposarcome
C. Kyste surinfecté
D. Mélanome nodulaire
E. Lymphome

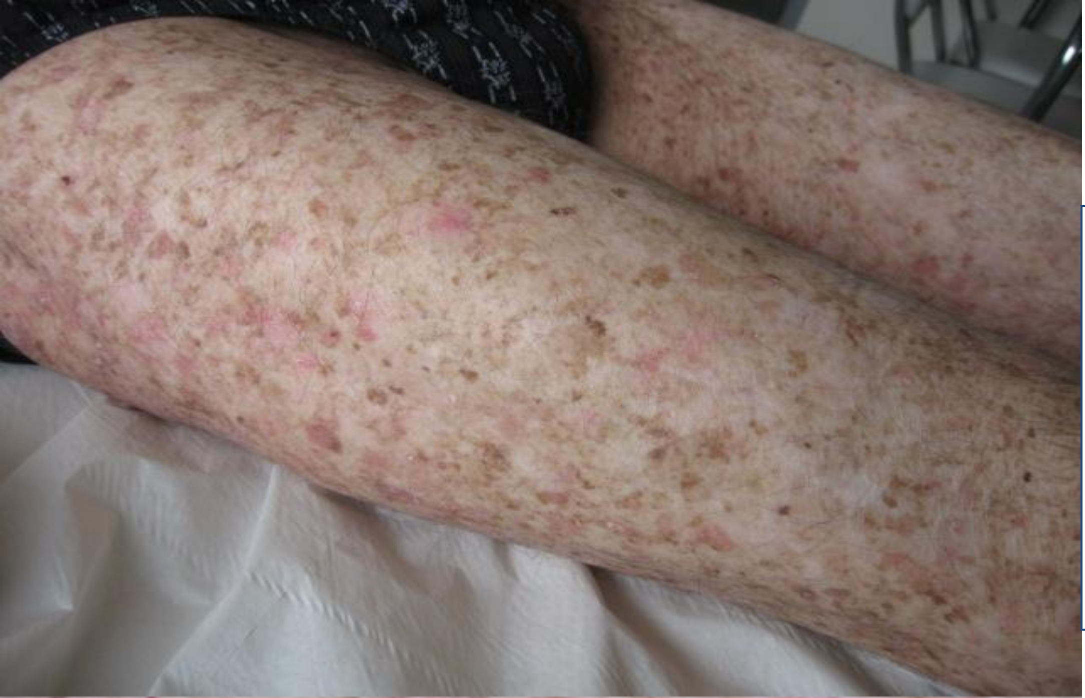

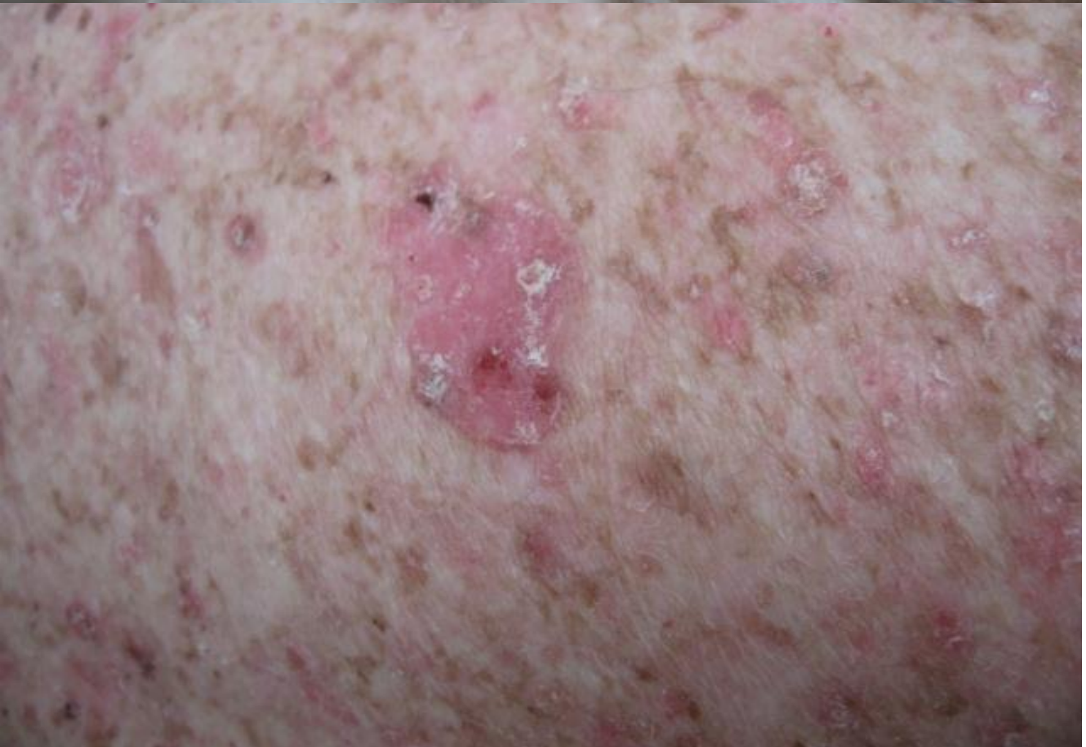

Homme, 55 ans. Psoriasis et rhumatisme psoriasique.
Traitements antérieurs par PUVA et rétinoïdes à multiples reprises (> 200 séances).
Début Méthotrexate 20 mg/ semaine depuis 3 mois pour psoriasis très étendu et rhumatisme actif.
Rhumatisme devenu quiescent et modification rapide de l’état cutané. Multiples lésions érythémateuses.
Absence de prurit.

A. Bénin
B. Pré cancéreux ou malin

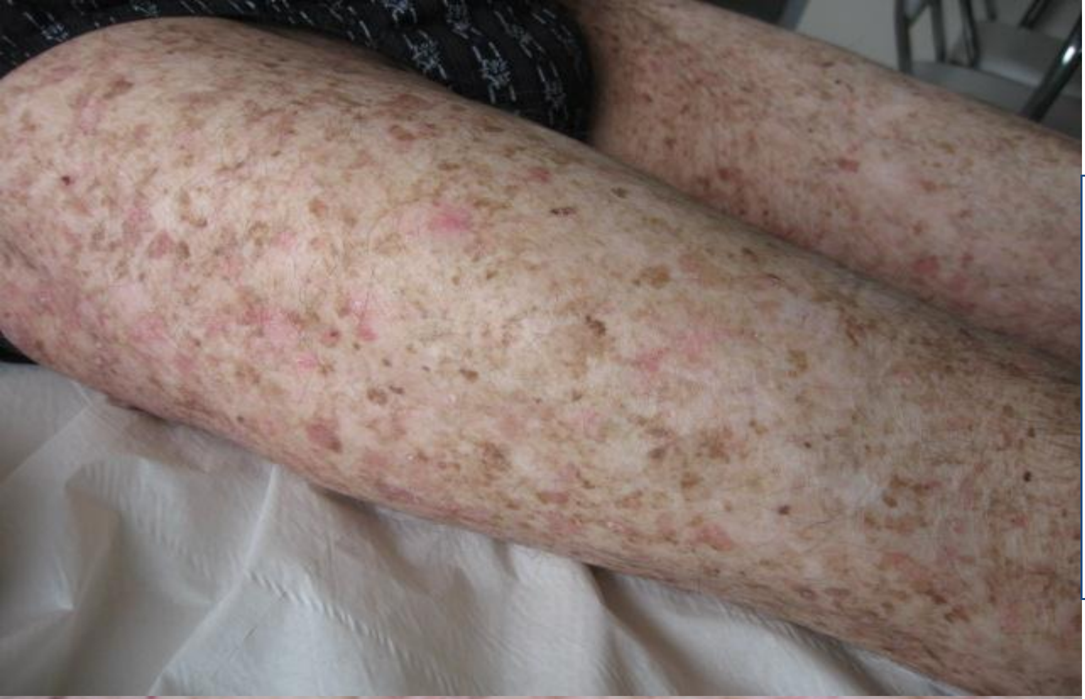

Homme, 55 ans. Psoriasis et rhumatisme psoriasique.
Traitements antérieurs par PUVA et rétinoïdes à multiples reprises (> 200 séances).
Début Méthotrexate 20 mg/ semaine depuis 3 mois pour psoriasis très étendu et rhumatisme actif.
Rhumatisme devenu quiescent et modification rapide de l'état cutané. Multiples lésions érythémateuses.
Absence de prurit.

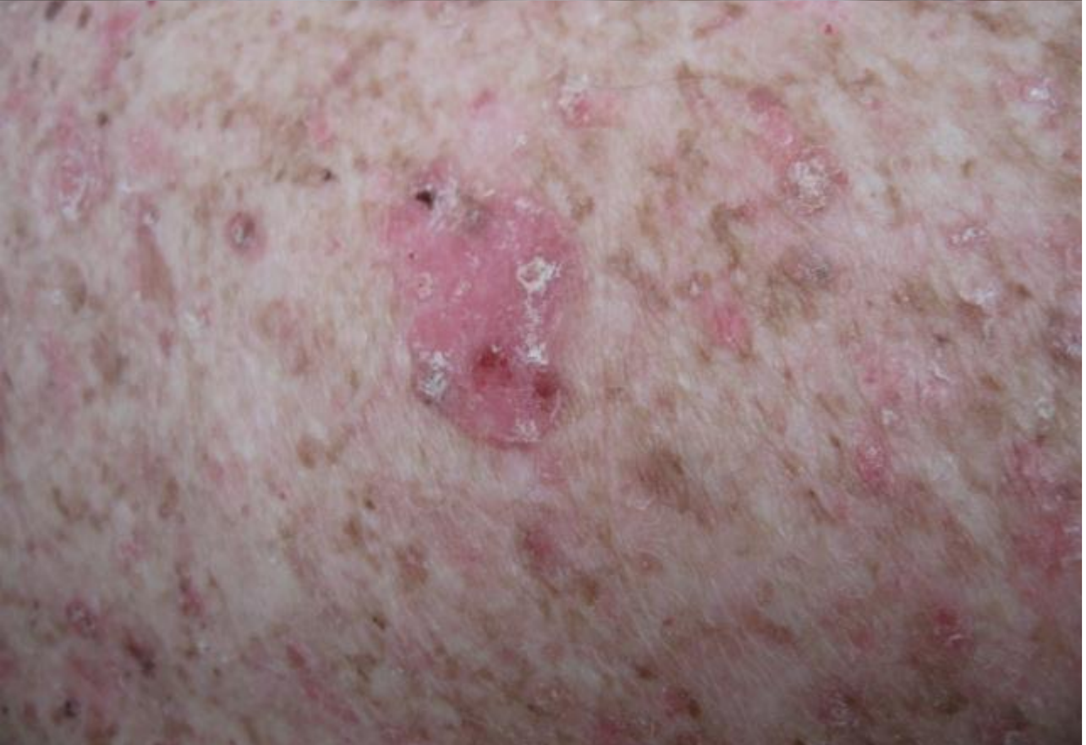

S'agit-il de:

A. Psoriasis en plaques résiduel
B. Eruption psoriasiforme paradoxale
C. Lymphome cutané sous méthotrexate
D. Kératoses actiniques multiples sur héliodermie
E. Toxidermie au méthotrexate

Homme 80 ans, suivi pour mélanome de la tempe gauche. Découverte fortuite lésion pigmentée du dos non repérée préalablement par le patient, asymptomatique, de petite taille (2-3 mm), discrètement en relief. Absence adénopathie périphérique.
(2 vues de la même lésion présentées)

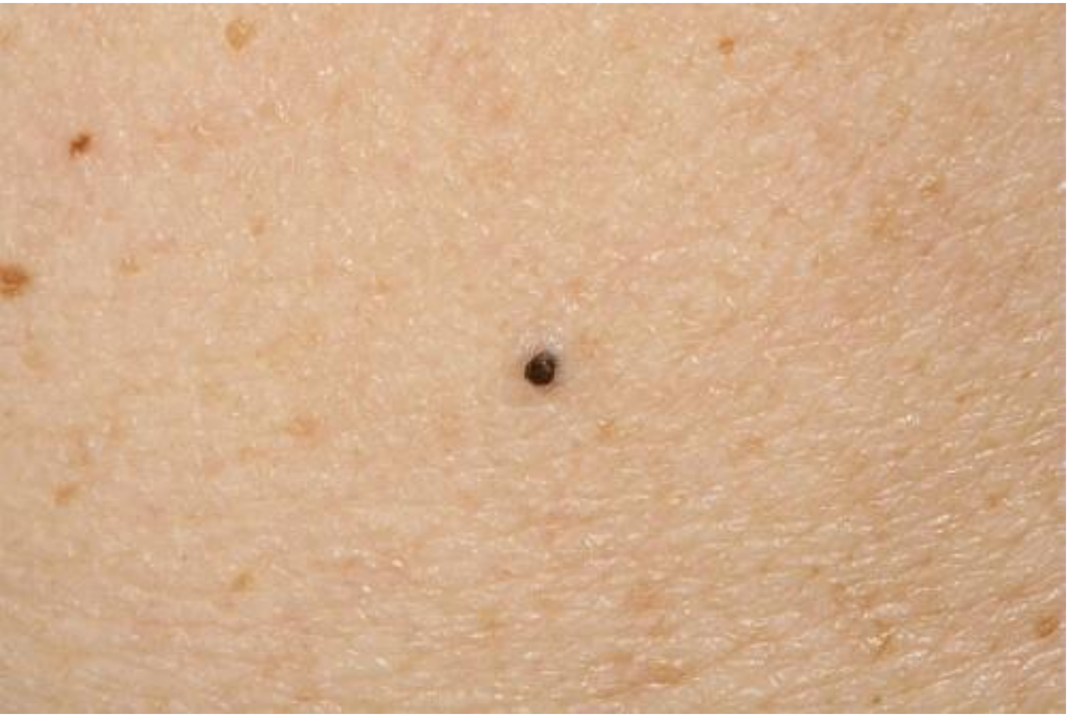

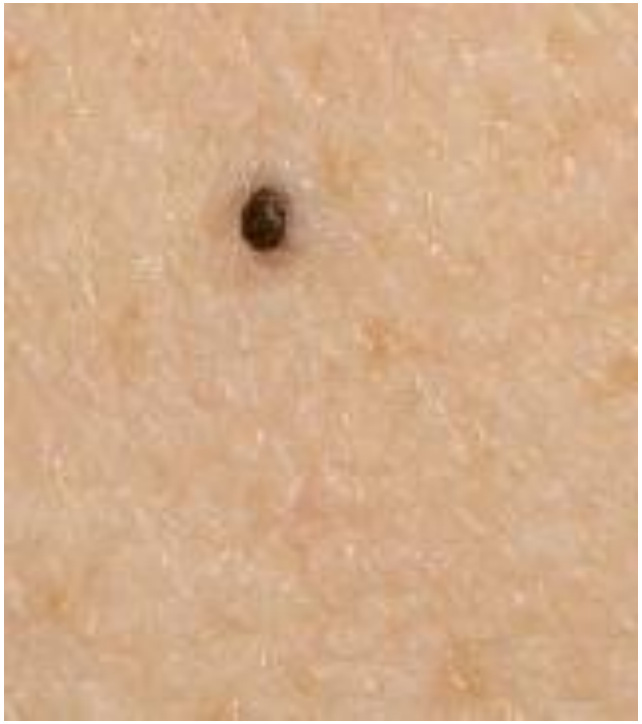

A. Bénin
B. Pré-cancéreux ou malin

Homme 80 ans, suivi pour mélanome de la tempe gauche. Découverte fortuite lésion pigmentée du dos non repérée préalablement par le patient, asymptomatique, de petite taille (2-3 mm), discrètement en relief. Absence adénopathie périphérique.
(2 vues de la même lésion présentées)

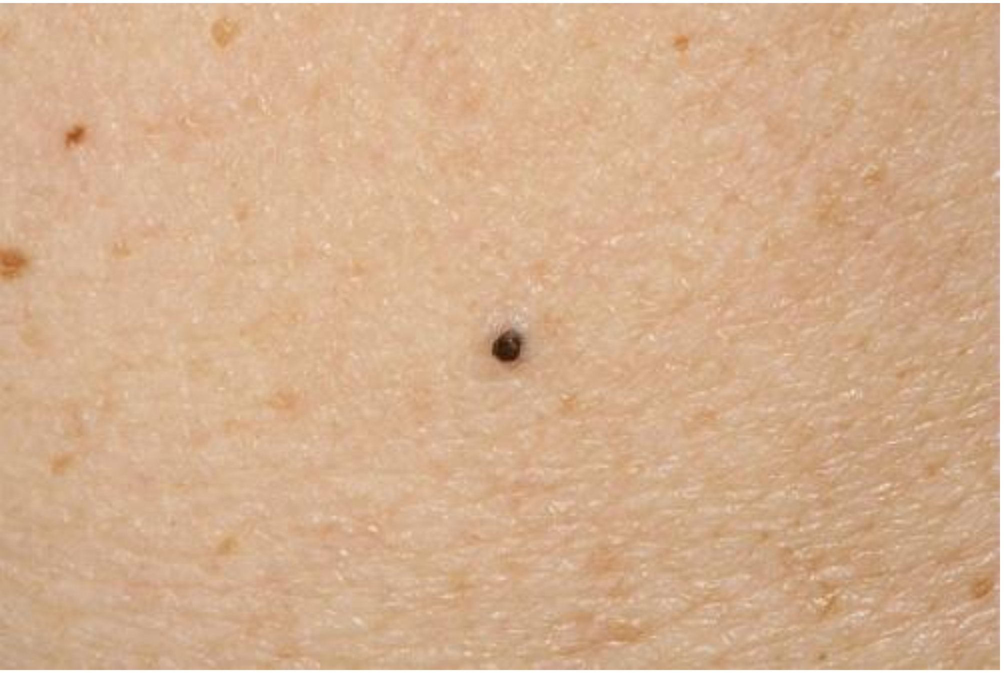

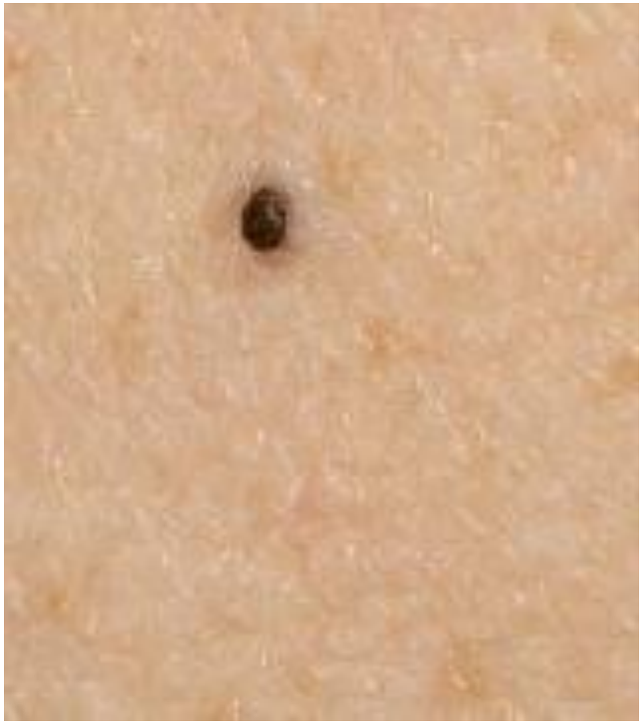

S’ agit-il de:

A. Un autre mélanome primitif
B. Une métastase cutanée de mélanome
C. Un carcinome baso cellulaire pigmenté
D. Un comédon (« point noir »)
E. Un histiocytofibrome

Femme 35 ans suivie pour SPA, traitée par Enbrel depuis 2 ans.
Lésion pigmentée en relief face externe bras droit, dure au toucher, non fluctuante, facilement préhensible entre les doigts, entraînant alors une invagination.
Absence adénopathie axillaire homolatérale.

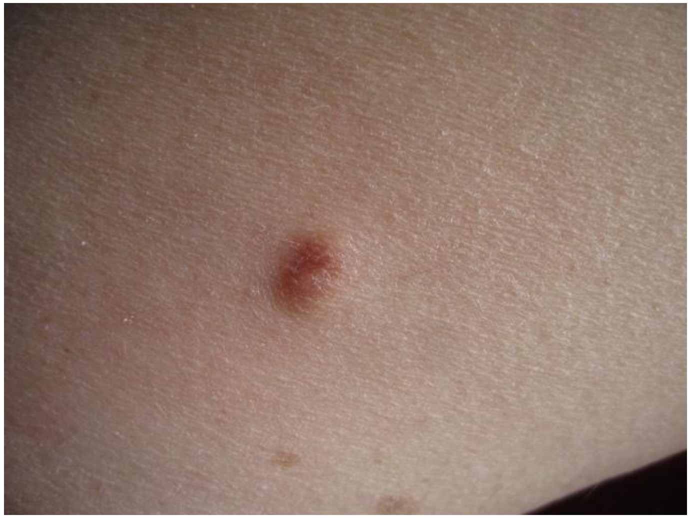

A. Bénin
B. Malin ou pré-cancéreux

Femme 35 ans suivie pour SPA, traitée par Enbrel depuis 2 ans.
Lésion pigmentée en relief face externe bras droit, dure au toucher, non fluctuante, facilement préhensible entre les doigts, entraînant alors une invagination.
Absence adénopathie axillaire homolatérale.

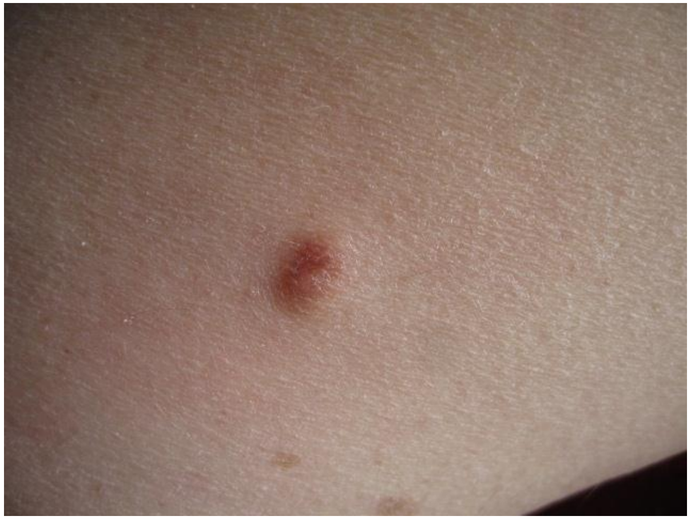

S' agit-il de:

A. Sarcome cutané
B. Mélanome
C. Naevus dermique
D. Histiocytofibrome
E. Carcinome baso-cellulaire pigmenté

Homme, 55 ans. PR traitée par MTX depuis 12 ans + DID mal équilibré.
Lésion du talon s'étendant progressivement depuis 6 mois.
Pas de notion de traumatisme.
Asymptomatique, traitements locaux inefficaces.

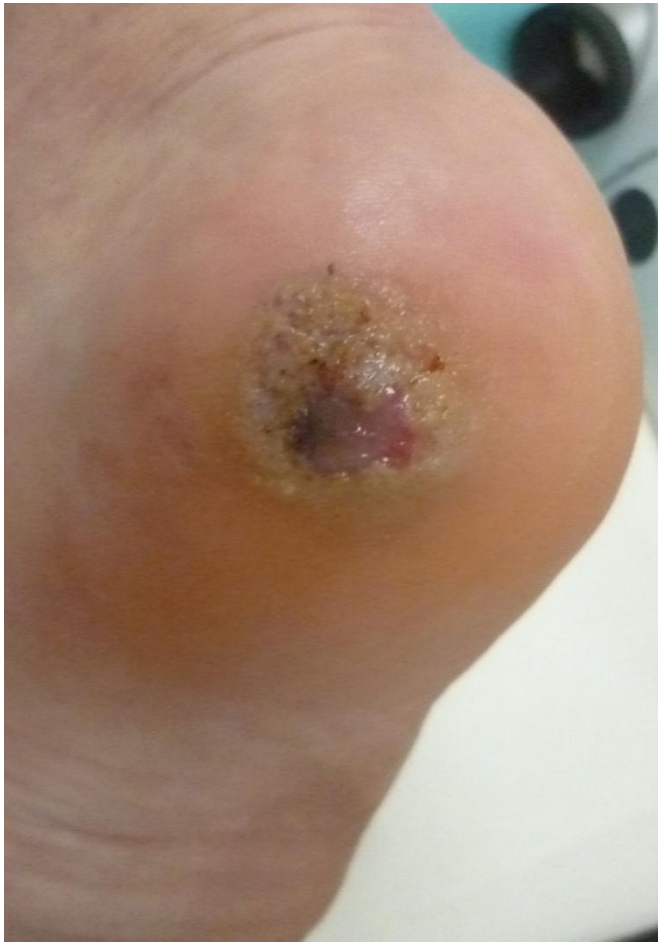

A. Bénin
B. Pré-cancéreux ou malin

Homme, 55 ans. PR traitée par MTX depuis 12 ans + DID mal équilibré.
Lésion du talon s'étendant progressivement depuis 6 mois.
Pas de notion de traumatisme.
Asymptomatique, traitements locaux inefficaces.

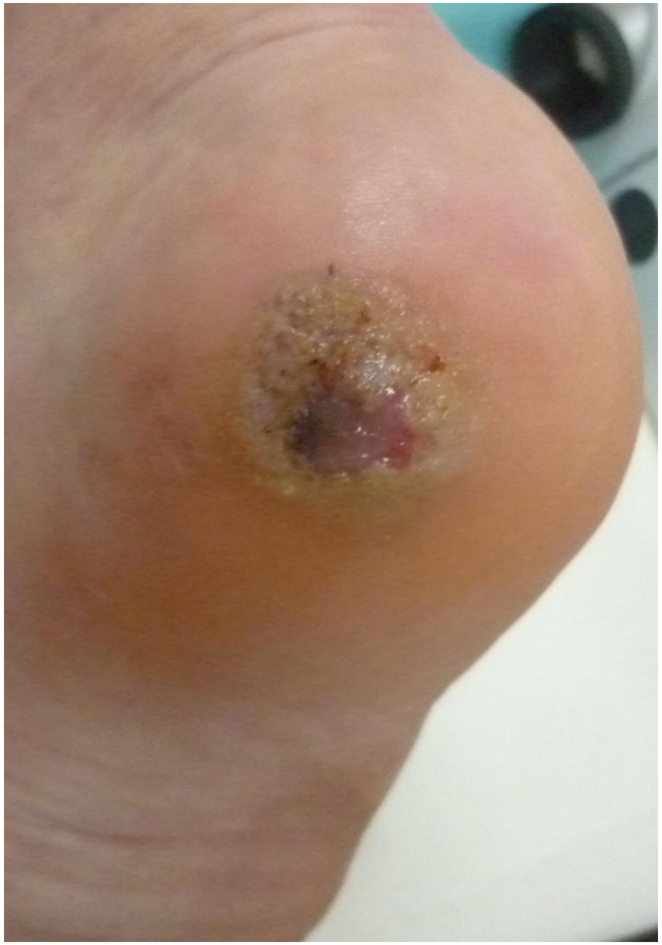

S'agit-il de:

A- Mal perforant plantaire
B- Carcinome basocellulaire
C- Eczéma de contact
D- Mélanome
E- Verrue plantaire

Femme 74 ans, diabétique. Plusieurs lésions rugueuses du dos des mains, non prurigineuses, non douloureuses, qu'elle « gratte pour faire tomber les croûtes ». Respect des paumes. Lésions d'aspect similaire sur front et le nez. Pas d'arthralgies, pas de myalgies.

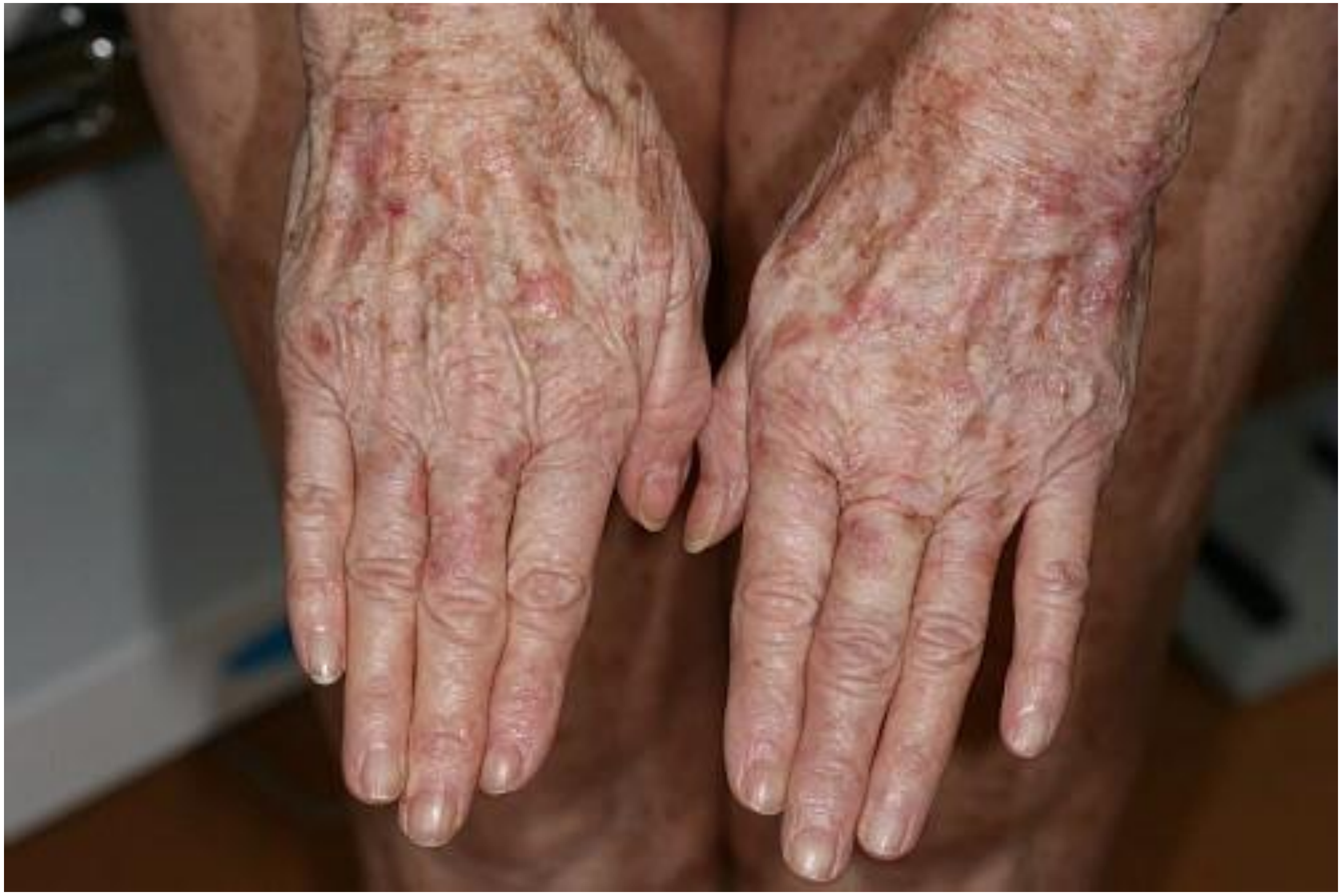

A. Bénin
B. Pré-cancéreux ou malin

Femme 74 ans, diabétique. Plusieurs lésions rugueuses du dos des mains, non prurigineuses, non douloureuses, qu'elle « gratte pour faire tomber les croûtes ». Respect des paumes. Lésions d'aspect similaire sur front et le nez. Pas d'arthralgies, pas de myalgies.

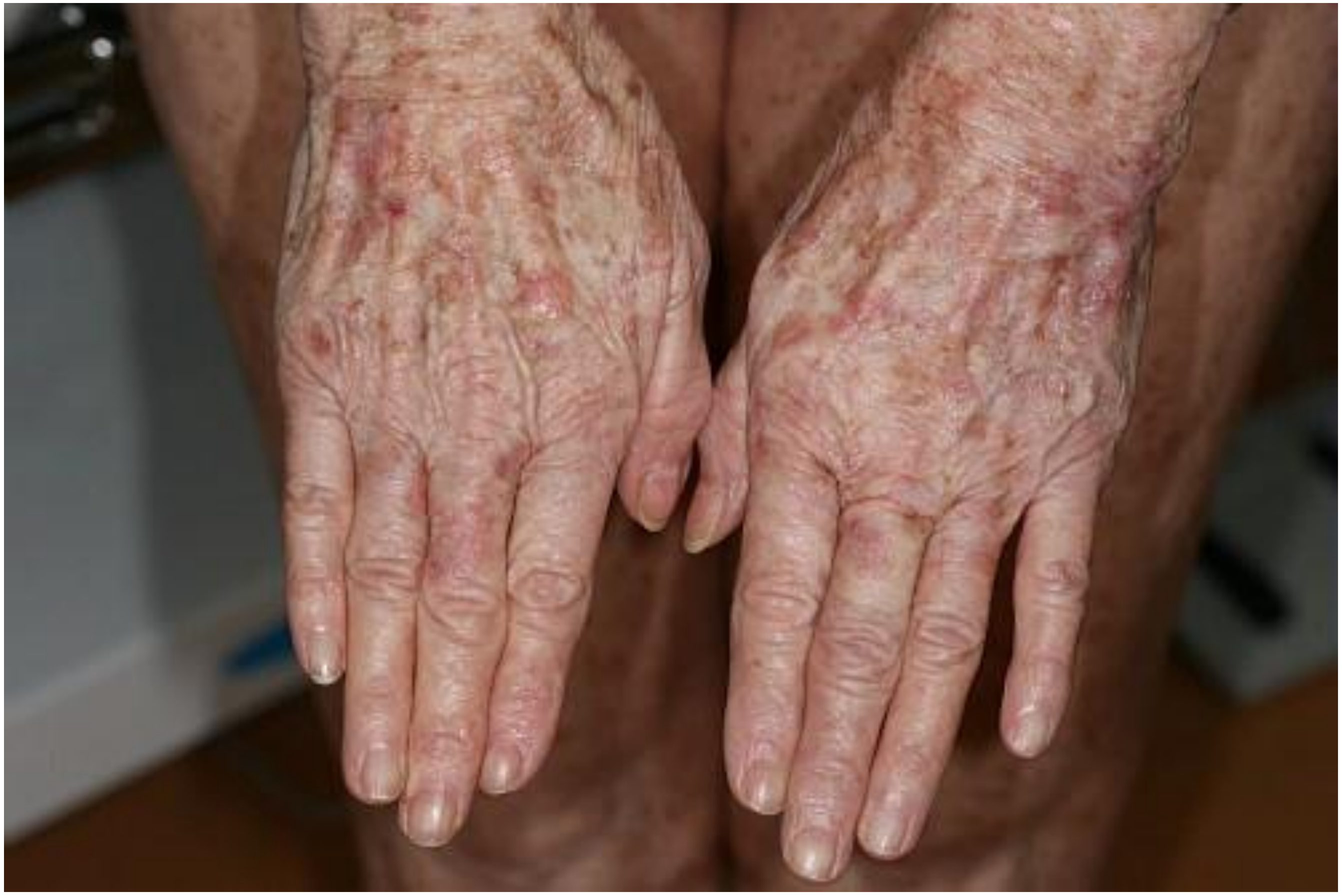

S'agit-il de :

A. Dermatomyosite amyopathique
B. Lupus érythémateux discoïde
C. Eczéma de contact
D. Kératoses actiniques
E. Dermatophytie profuse (infection cutanée à champignon)

Lésion face postérieure de la jambe droite chez une femme de 74 ans, niçoise, non douloureuse, non prurigineuse, présente depuis au moins 6 mois, avec tendance à l’extension lentement progressive.

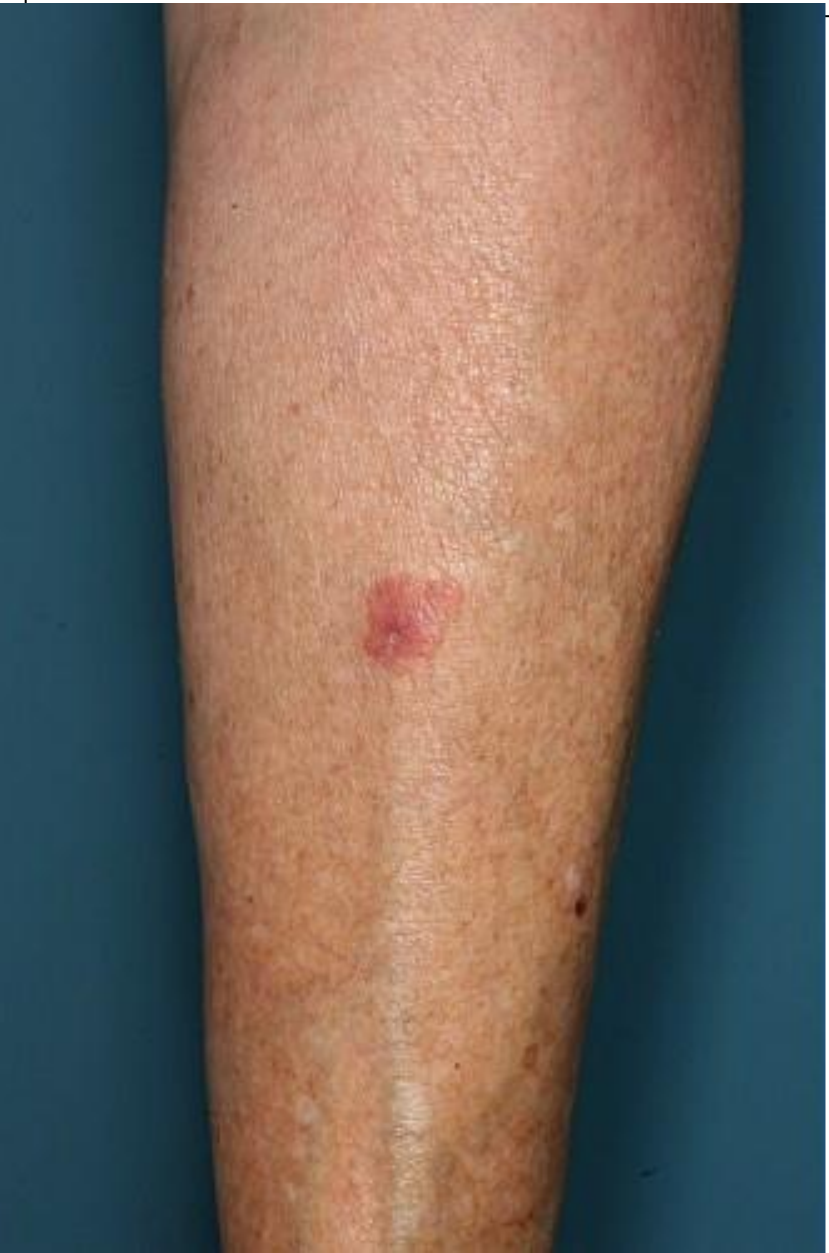

S’ agit-il de :

A. Bénin

B. Pré-cancéreux ou malin

Lésion face postérieure de la jambe droite chez une femme de 74 ans, niçoise, non douloureuse, non prurigineuse, présente depuis au moins 6 mois, avec tendance à l'extension lentement progressive.

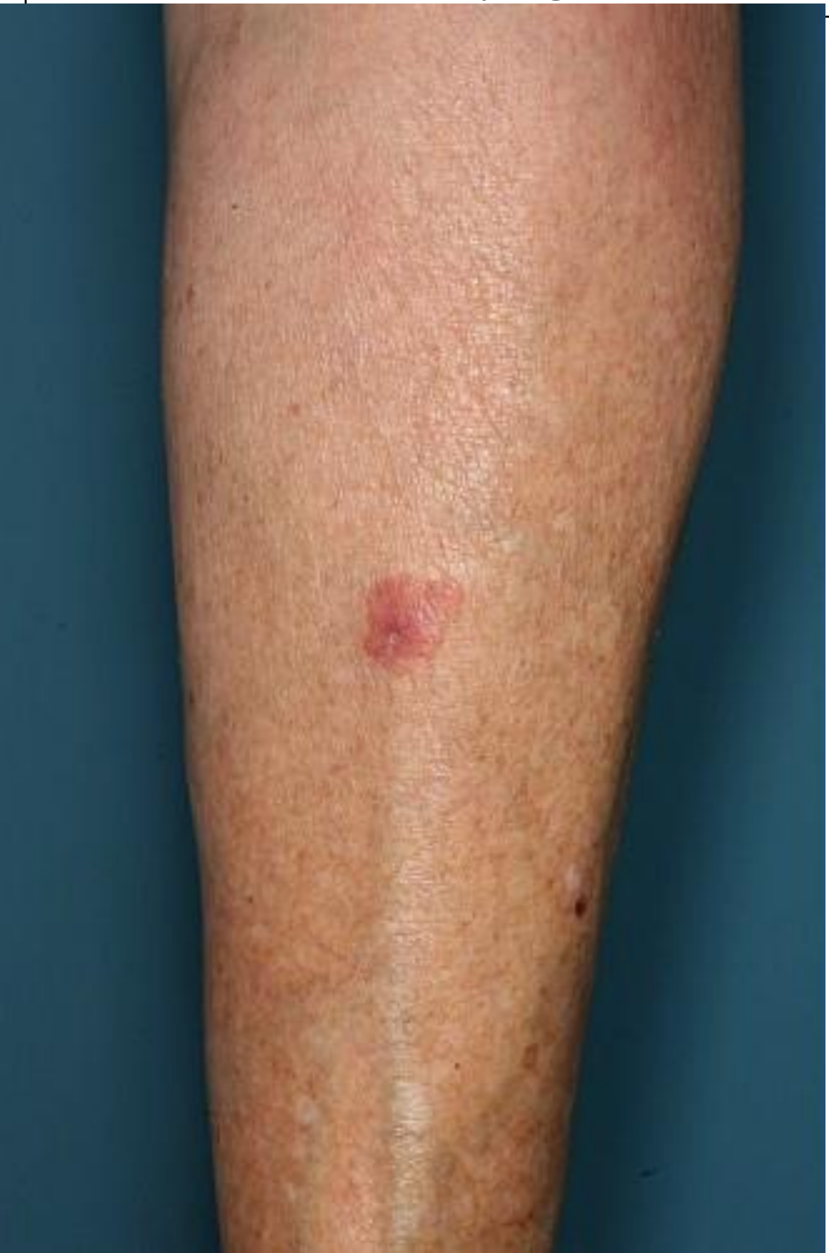

S' agit-il de :

A. Dermatophytie (infection cutanée mycosique)
B. Psoriasis vulgaire
C. Eczéma nummulaire (en forme de pièce de monnaie)
D. Maladie de Bowen (carcinome épidermoïde *in situ*)
E. Maladie de Kaposi

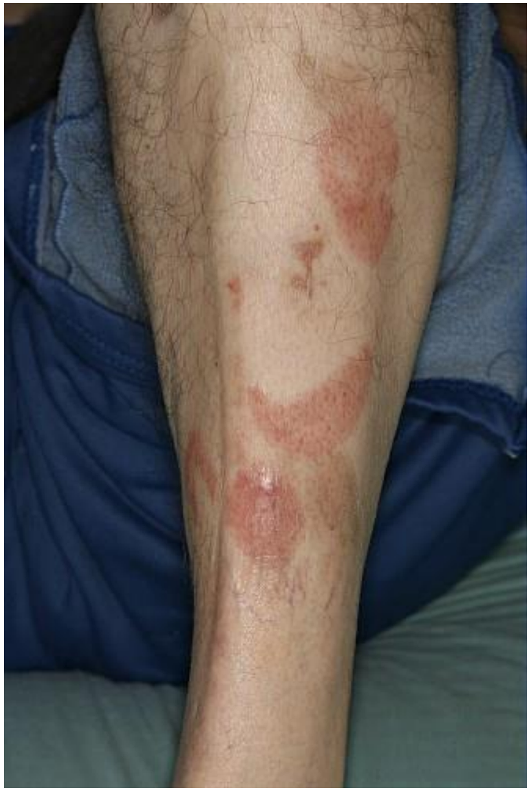
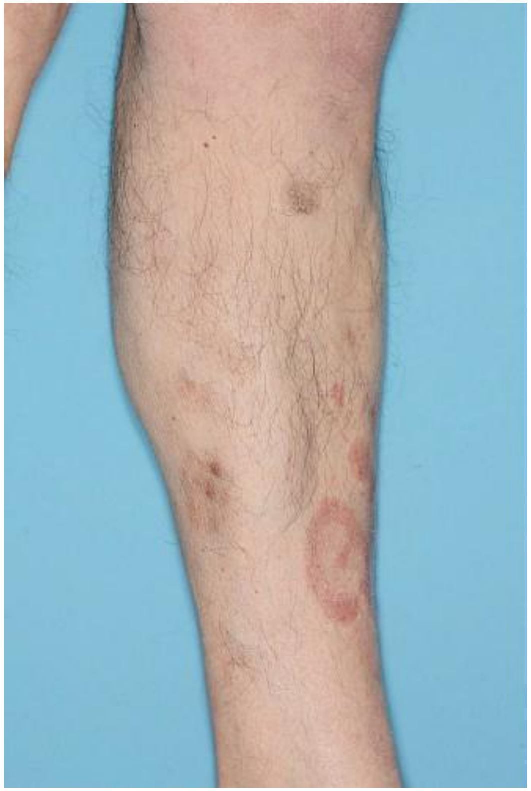
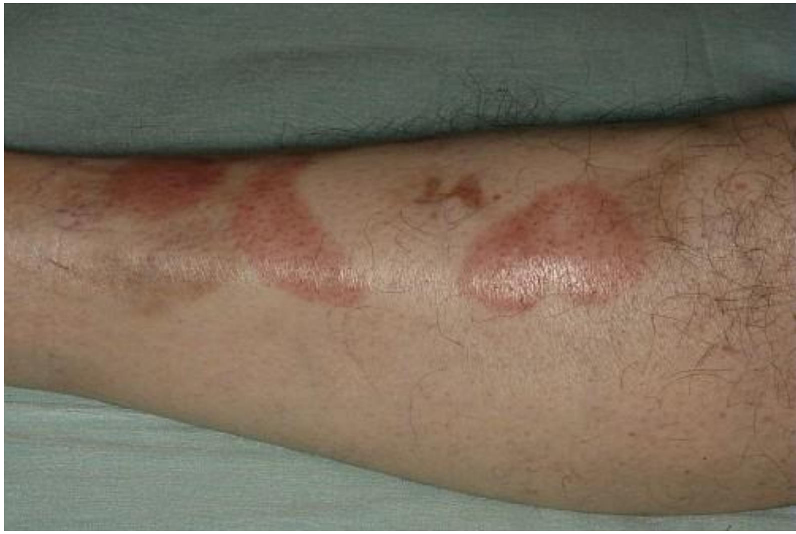

Homme, 55 ans. PR en rémission sous Adalimumab depuis 2 ans + DID mal équilibré.
Voyage pendant 15 jours au Mexique, il y a 8 mois.
Lésions des jambes s'étendant progressivement depuis 6 mois.
Prurit modéré.

A. Bénin
B. Pré-cancéreux ou malin

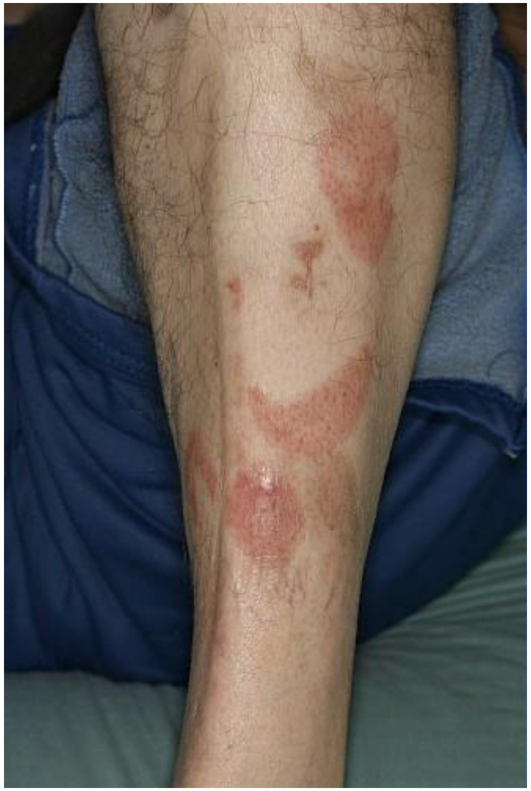
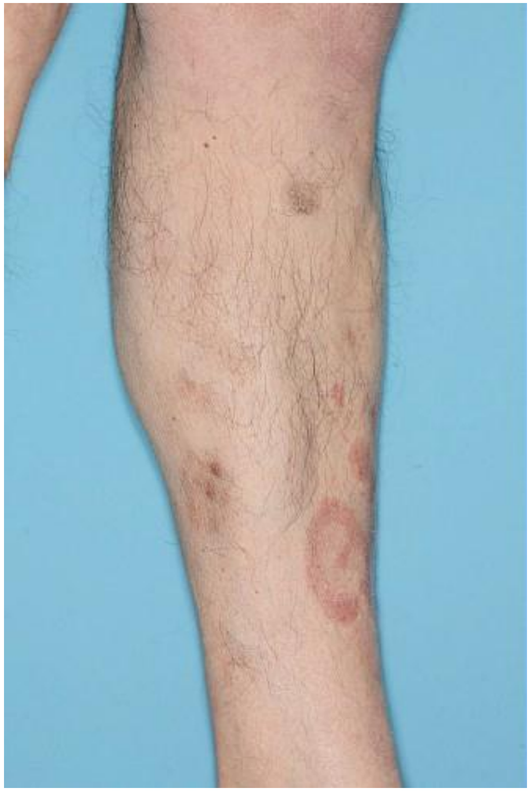
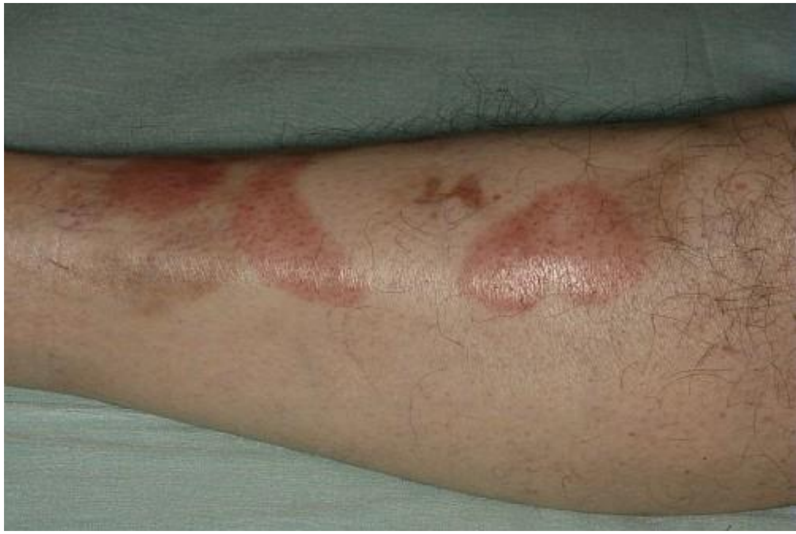

Homme, 55 ans. PR en rémission sous Adalimumab depuis 2 ans + DID mal équilibré.
Voyage pendant 15 jours au Mexique, il y a 8 mois.
Lésions des jambes s'étendant progressivement depuis 6 mois.
Prurit modéré.

<u>S'agit-il de:</u>

A. Psoriasis
B. Dermatophytie profuse (infection mycosique cutanée)
C. Erythème noueux
D. Sarcome de Kaposi
E. Lymphome cutané

Homme, 44 ans. Psoriasis traité par photothérapie depuis 12 ans.
Lésion digitale depuis 15 jours, légèrement douloureuse.
Notion de traumatisme.

A. Bénin
B. Pré-cancéreux ou malin

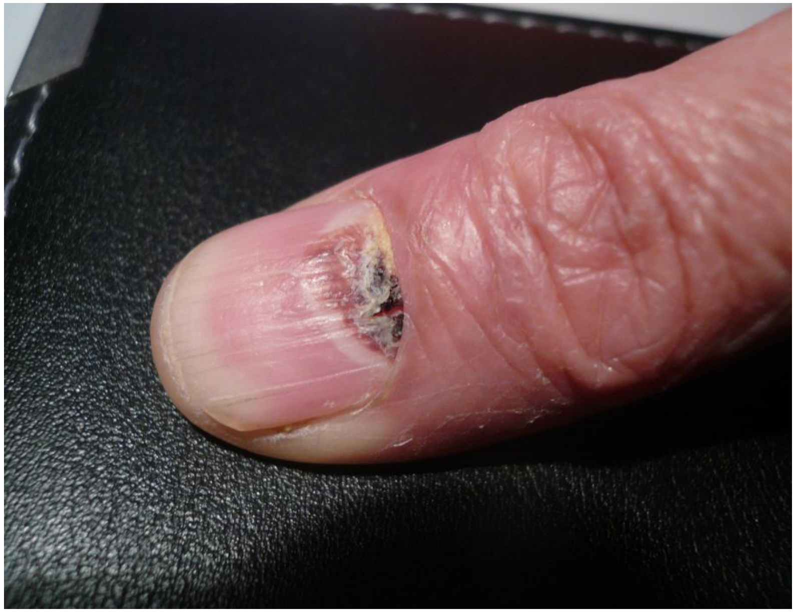

Homme, 44 ans. Psoriasis traité par photothérapie depuis 12 ans.
Lésion digitale depuis 15 jours, légèrement douloureuse.
Notion de traumatisme.

<u>S'agit-il de:</u>

A- Mélanome unguéal
B- Hématome sous-unguéal
C- Psoriasis unguéal
D- Lichen unguéal
E- Exostose sous-unguéale

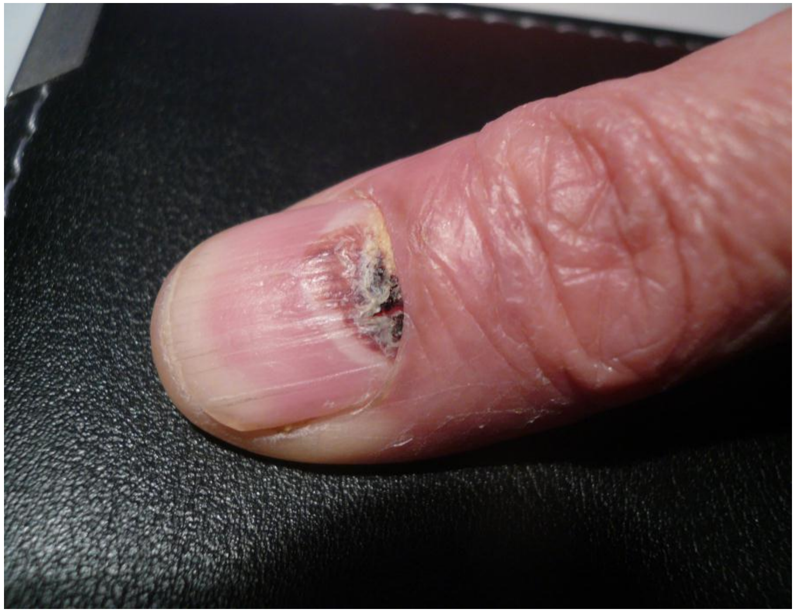

Supporting information 2:
Baseline evaluation
English

MCQ 1. Among the followings, which can be considered as risk factors for developing skin cancer in patients affected with inflammatory rheumatism ?
A - History of cutaneous carcinoma.
B - Patient with fair skin and frequent sunburns.
C - Smoking.
D – More than 200 PUVAtherapy sessions.
E - Family history of melanoma.

QCM 2. Among the followings, which are predisposing factors for developing skin cancer?
A – Duration of immunosuppressive treatment (ciclosporin, mycophenolate mofetil, azathioprine…).
B – Asbestos exposure.
C – Important past sun exposure.
D – Regular use of indoor tanning.
E – Number of phototherapy sessions.

QCM 3. Which photoprotection advices do you give to patients receiving immunosuppressive treatment ?
A – Stay in the shade during midday hours (the most hazardous hours).
B – Prefer protection with clothes and hat rather than protection with sunscreens.
C – Use high SPF sunscreens in order to spend the whole day on the beach.
D – Select the SPF of the sunscreen in fonction of the skin type of the patient.
E – Prepare the skin to sun by using artificial UV radiation.

QCM 4. The following situations are classical contra-indications to TNF alpha blockers :
A. Nodular basal cell carcinoma of the back completely resected by surgery 1 year ago.
B. Epidermoid carcinoma of the leg with metastatic lymph node, both completely resected with surgery 2 years ago.
C. Past history of malignant lentigo in the mother of the patient.
D. Dysplastic naevi syndrom.
E. Nodular melanoma of the leg, Clark IV, Breslow 2.3 mm, completely resected with 2 cm margins 6 years ago.

QCM 5. Which, among the followings, are true propositions ?
A. Basal cell carcinoma is a frequent skin tumor of good prognosis.
B. No visceral metastasis can be observed with squamous cell carcinoma .
C. Most of melanomas are definitively cured, thanks to early surgical resection.
D. The survival of melanoma with visceral metastasis usually ranges from 6 to 9 months.
E. Mucosal squamous cell carcinomas have a worse prognosis, compared to skin squamous cell carcinomas.

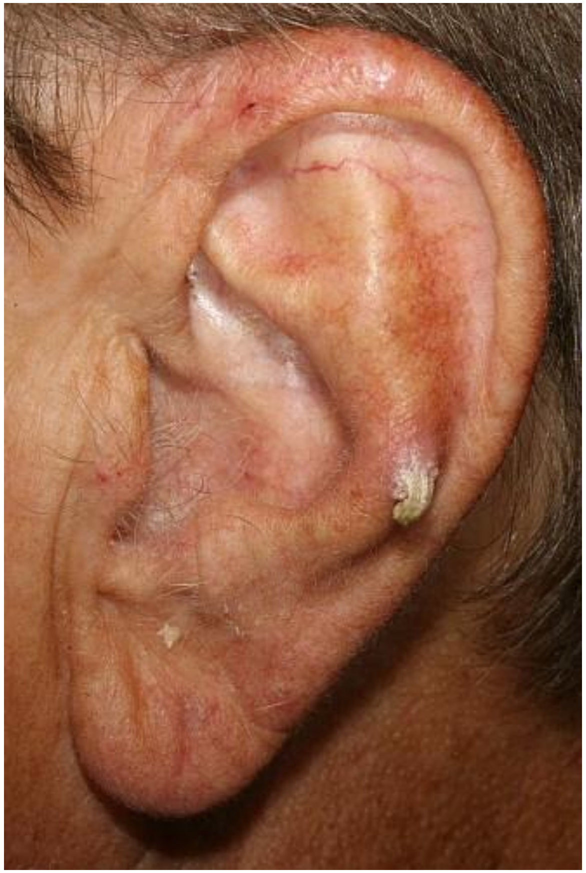 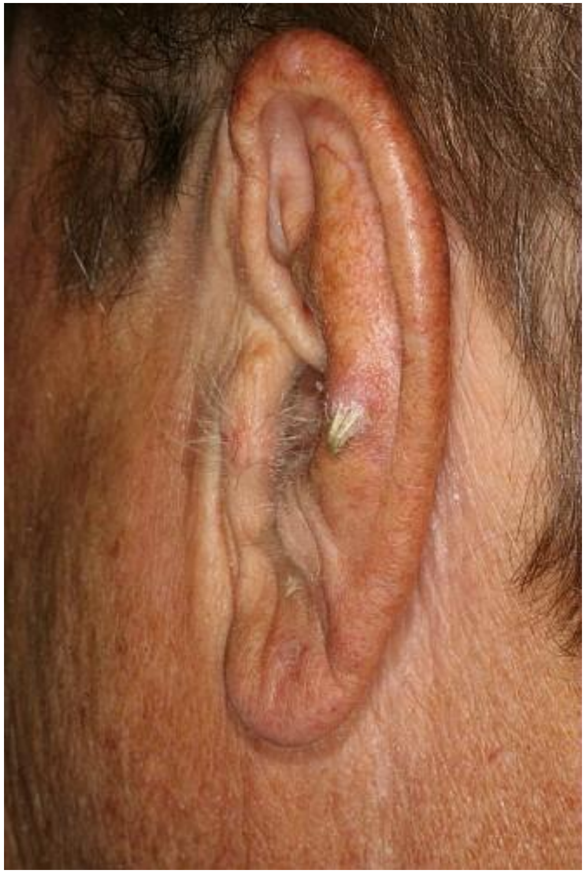

Skin lesion of the ear, for more than 1 year ago in a 70-year-old male with past history of basal cell carcinoma of the face.
Slow increase in size. No bleeding. No pain.

A. Benign
B. Pre-malignant or malignant

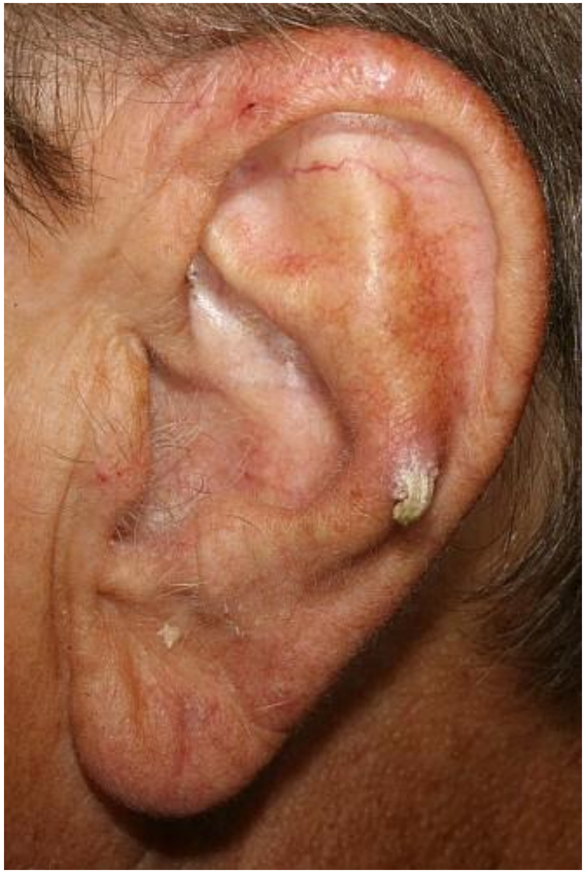

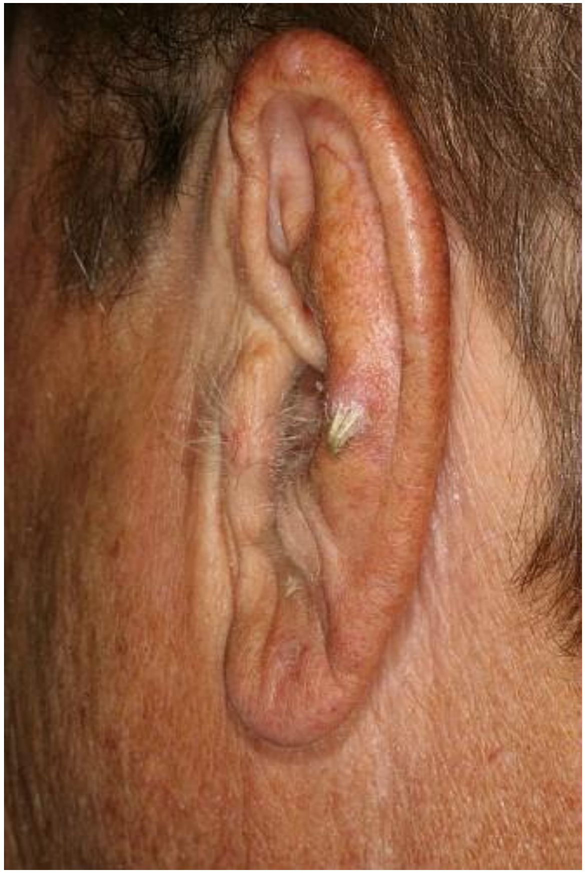

Skin lesion of the ear, for more than 1 year ago in a 70-year-old male with past history of basal cell carcinoma of the face.
Slow increase in size. No bleeding. No pain.

<u>What is your diagnosis?:</u>

A. Molluscum pendulum
B. Viral papilloma
C. Actinic keratosis
D. Squamous cell carcinoma
E. Epidermal cyst

Female, 42 years-old. Psoriasis successfully treated with etanercept for 1 year (after failure of phototherapy and methotrexate).
Skin lesion of the foreharm for 3 months (when returning from Madagascar).
Symptom-free. Progressive extension

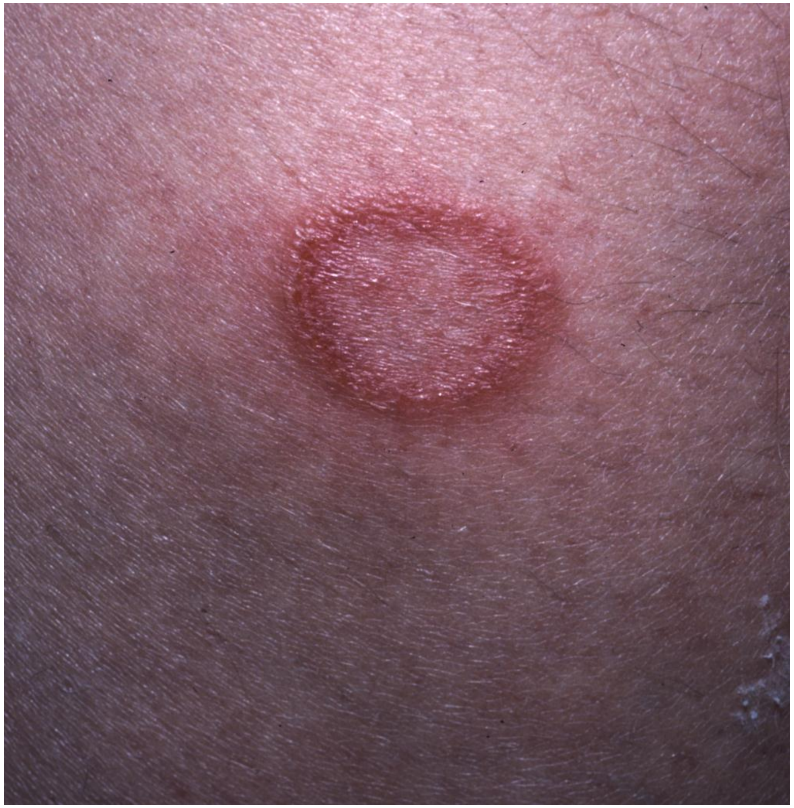

A. Benign
B. Pre-malignant or malignant

Female, 42 years-old. Psoriasis successfully treated with etanercept for 1 year (after failure of phototherapy and methotrexate).
Skin lesion of the foreharm for 3 months (when returning from Madagascar).
Symptom-free. Progressive extension.

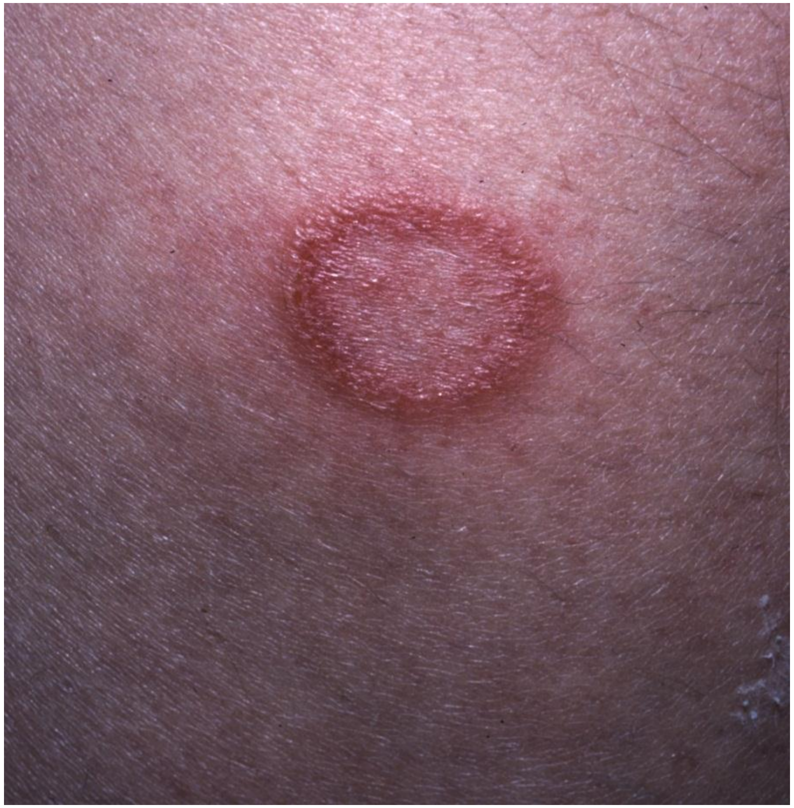

What is your diagnosis?:

A- Psoriasis
B- Bowen’s disease
C- Dermatophytosis
D- Superficial basal cell carcinoma
E- Cutaneous lymphoma

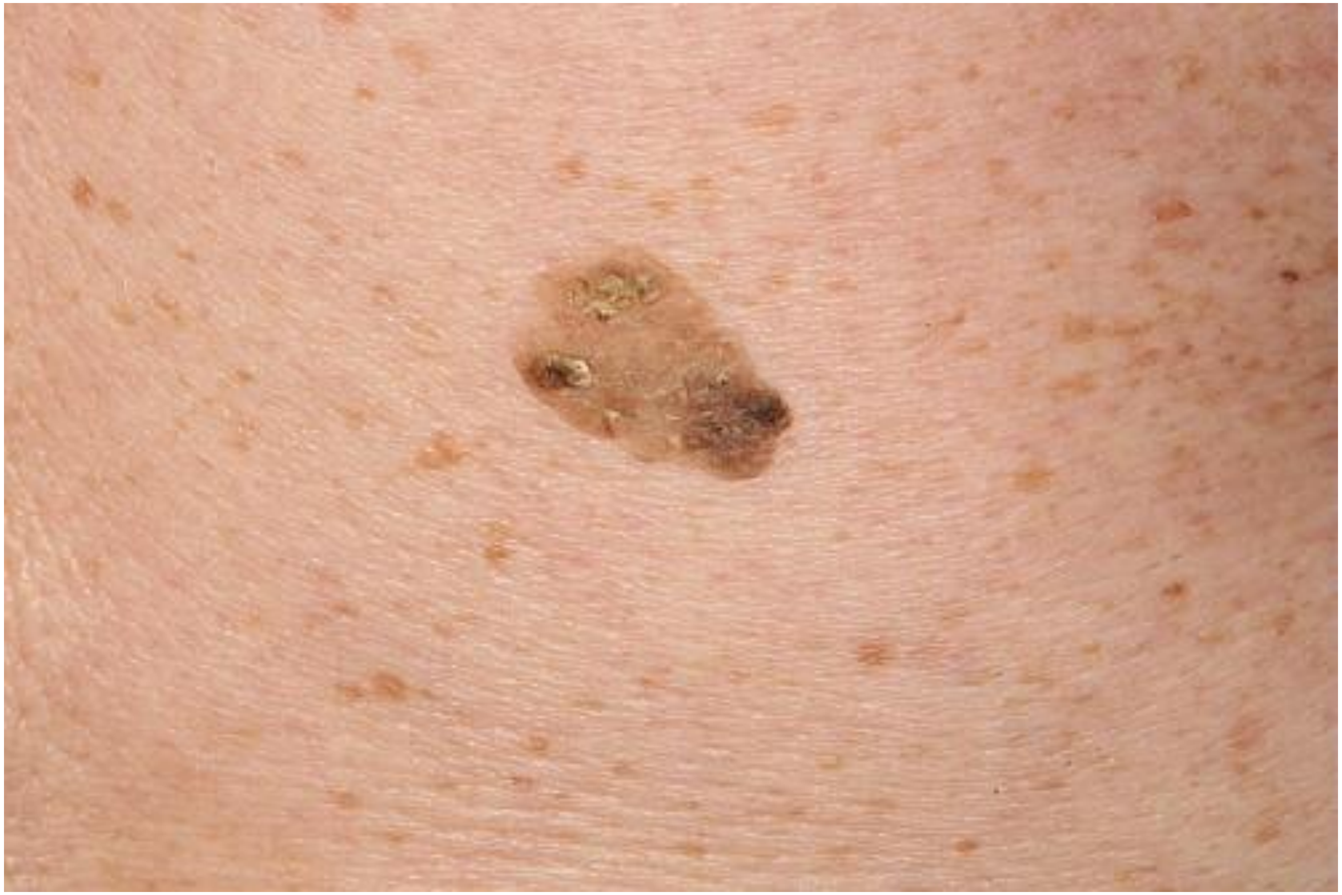

Skin lesion of the back in a 55-year-old female with past history of melanoma. Duration and evolution can not be precised by the patient.

A. Benign
B. Pre-malignant or malignant

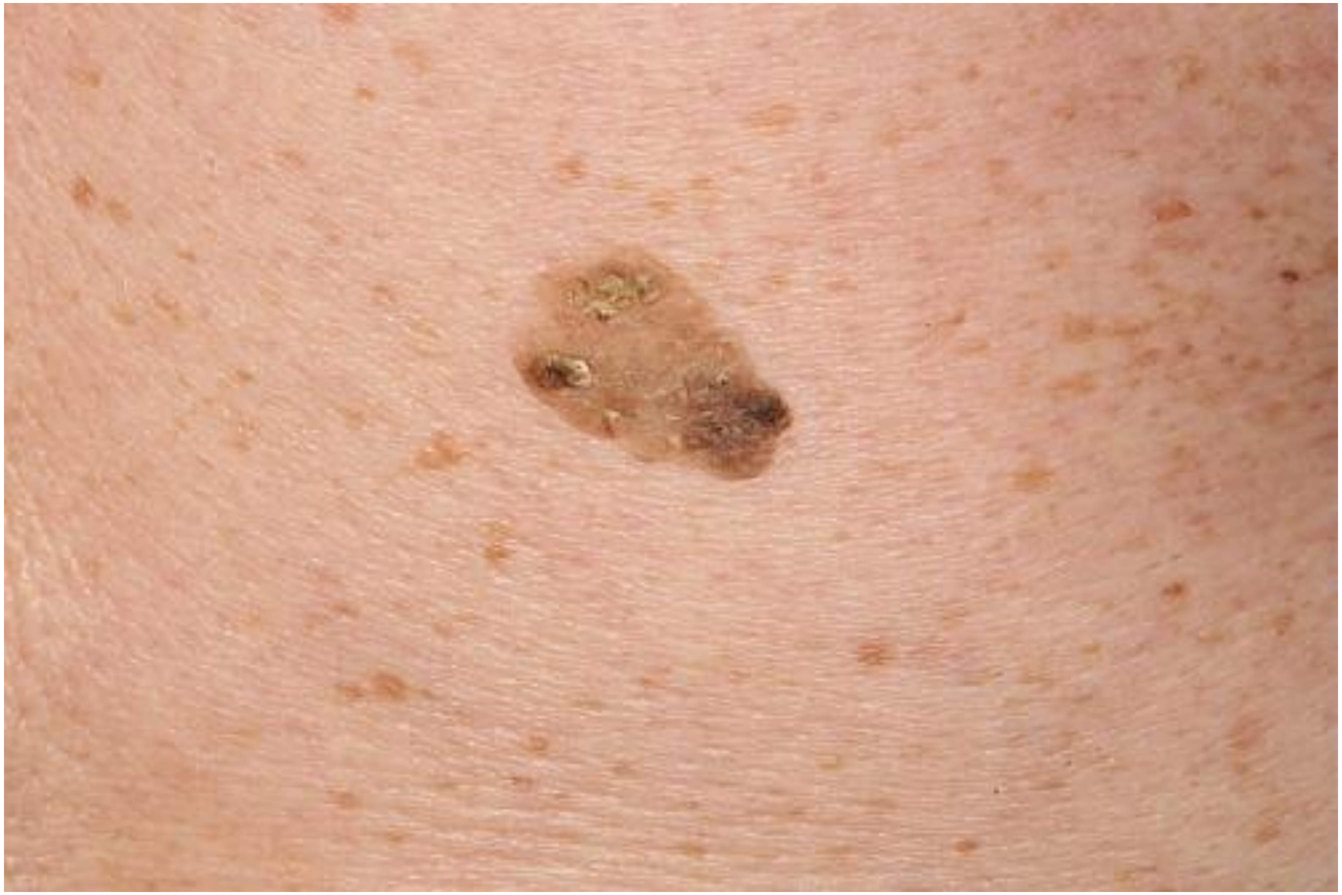

Skin lesion of the back in a 55-year-old female with past history of melanoma. Duration and evolution can not be precised by the patient.

What is your diagnosis?:

A. Seborrheic keratosis
B. Melanoma
C. Bowen's disease
D. Naevus
E. Basal cell carcinoma

Sole skin lesion (basis of the 5irth toe), painful in charge, in a 55-year-old woman with past history of melanoma, appeared several months ago, without healing.

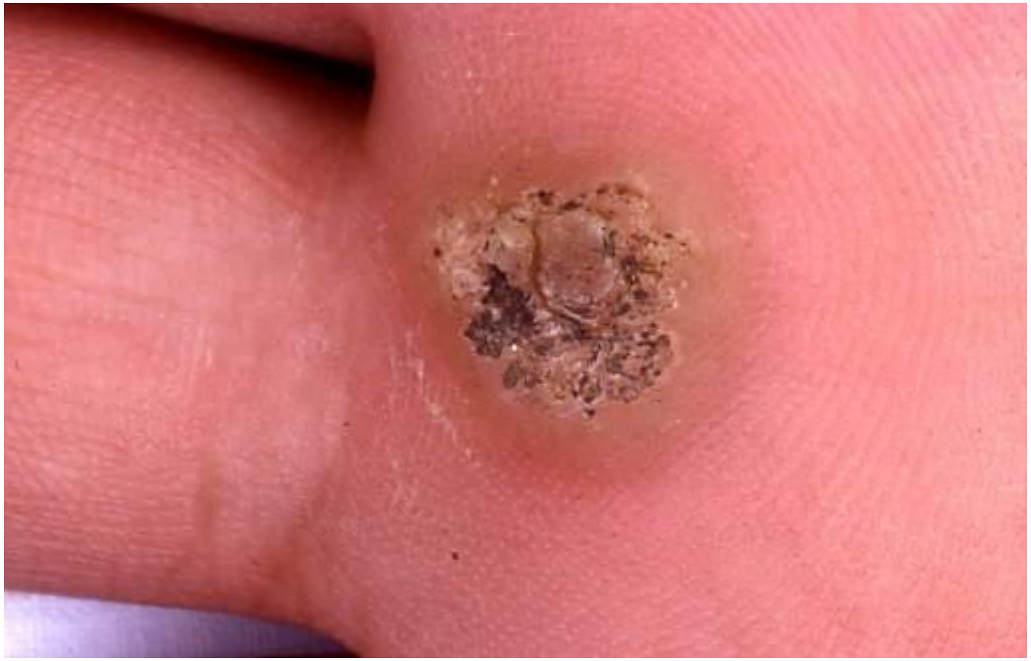

A. Benign
B. Pre-malignant or malignant

Sole skin lesion (basis of the 5irth toe), painful in charge, in a 55-year-old woman with past history of melanoma, appeared several months ago, without healing.

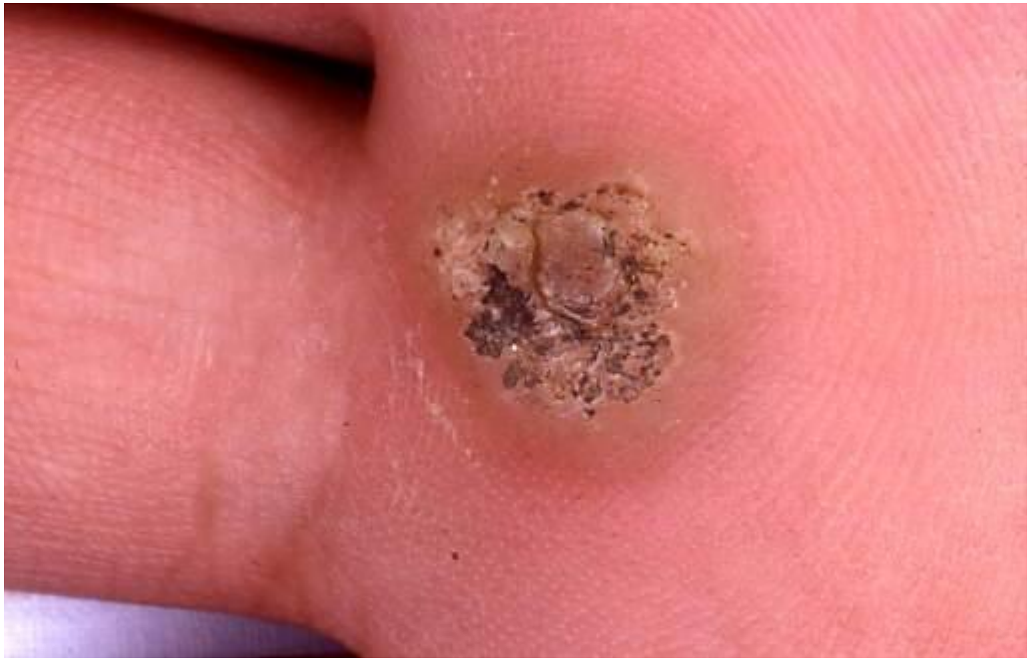

What is your diagnosis?:

A. Wart
B. Melanoma
C. Squamous cell carcinoma
D. Callus
E. Kaposi's sarcoma

Female, 75-years. Psoriasis treated with phototherapy for 20 years.
Cheek asymptomatic skin lesion, with slight size increase.

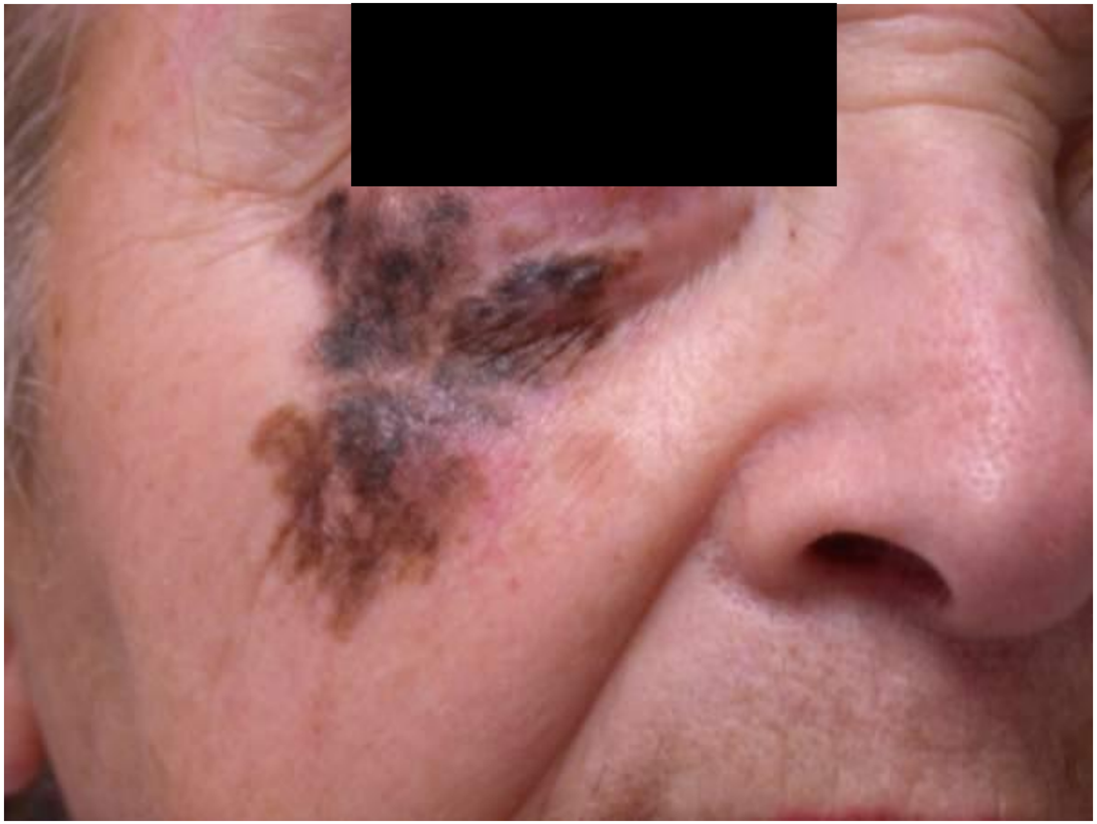

A. Benign
B. Pre-malignant or malignant

Female, 75-years. Psoriasis treated with phototherapy for 20 years.
Cheek asymptomatic skin lesion, with slight size increase.

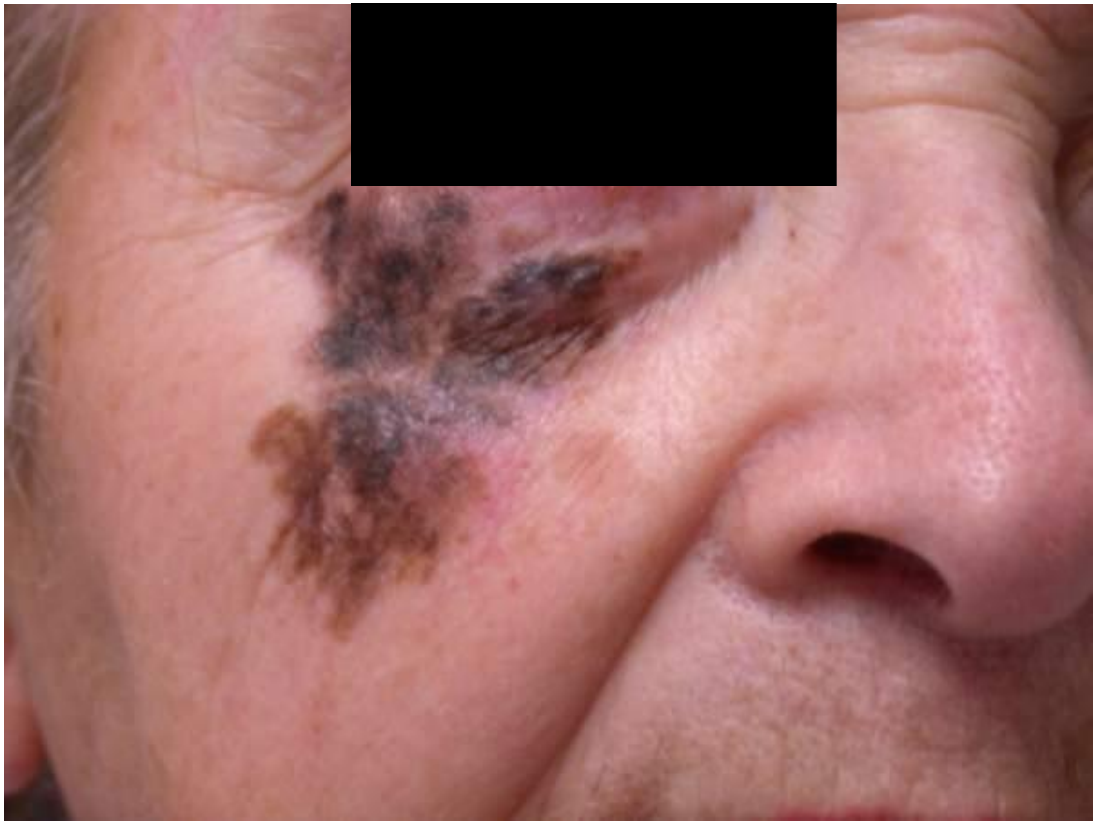

<u>What is your diagnosis?:</u>

A- Naevus
B- Melanoma
C- Lentigo
D- Pigmented basal cell carcinoma
E- Post trauma haematoma

Male, 40-years. Psoriasis treated with phototherapy then methotrexate.
Unilateral skin lesion close to the eye for 6 months.
No associated symptoms.
Wearing glasses.

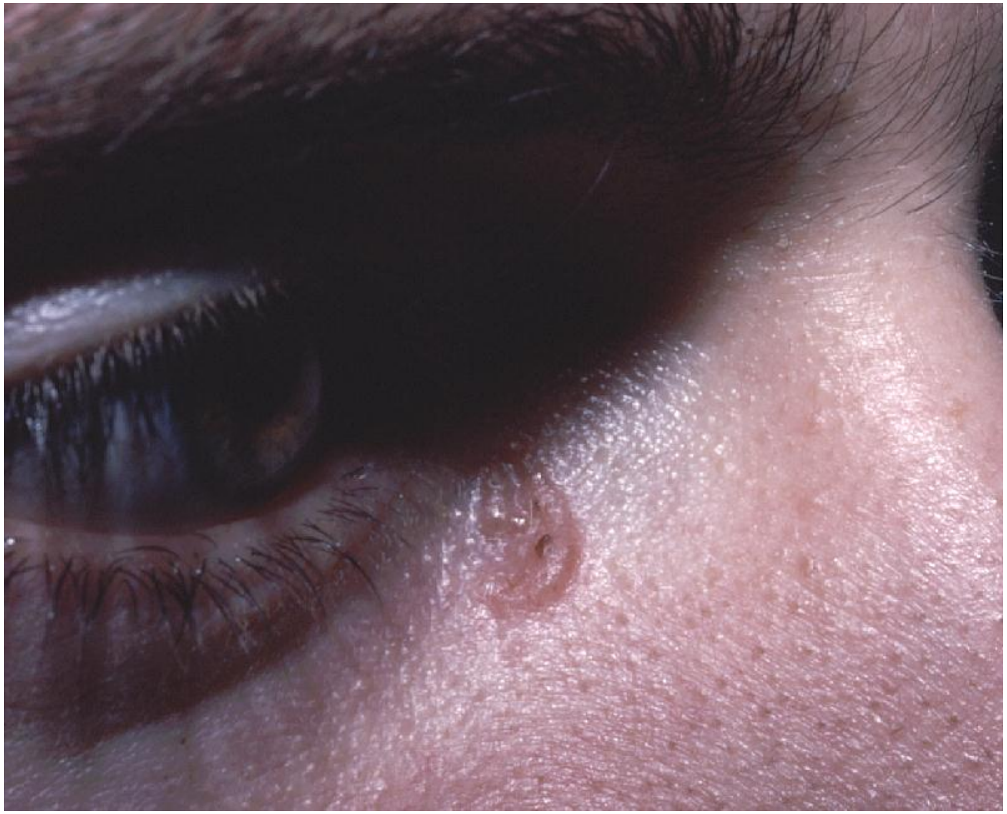

A. Benign
B. Pre-malignant or malignant

Male, 40-years. Psoriasis treated with phototherapy then methotrexate.
Unilateral skin lesion close to the eye for 6 months.
No associated symptoms.
Wearing glasses.

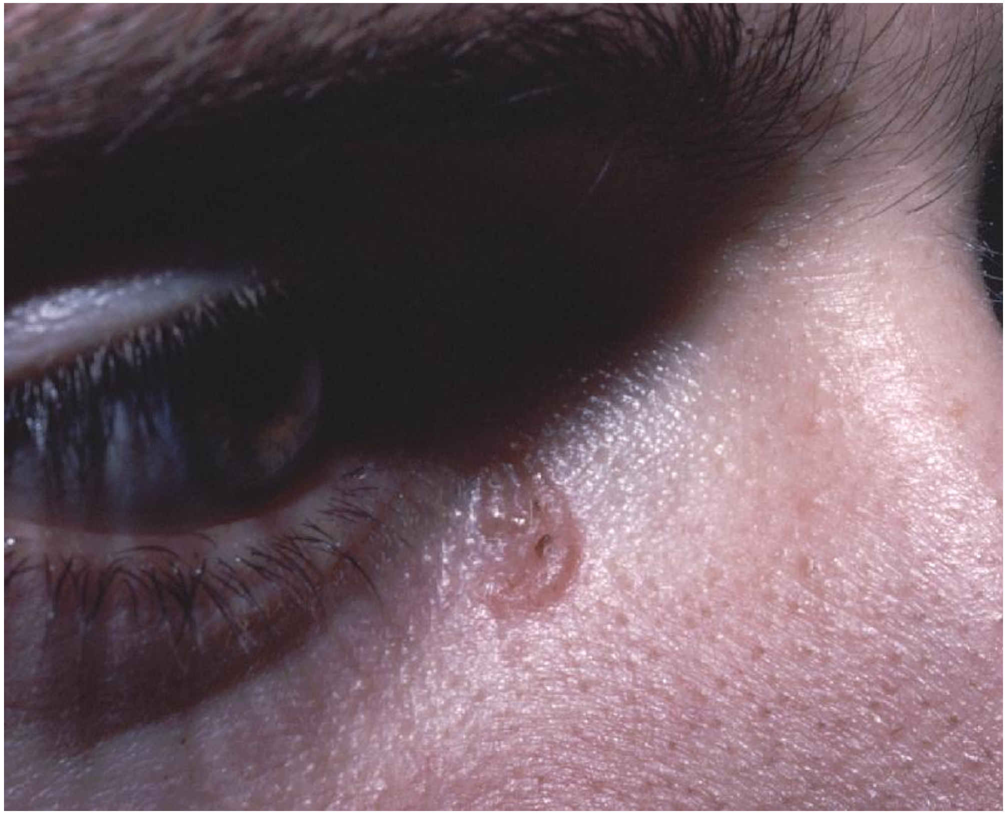

What is your diagnosis?:

A. Basal cell carcinoma
B. Melanoma
C. Xanthelasma
D. Actinic keratosis
E. Post trauma lesion (glasses)

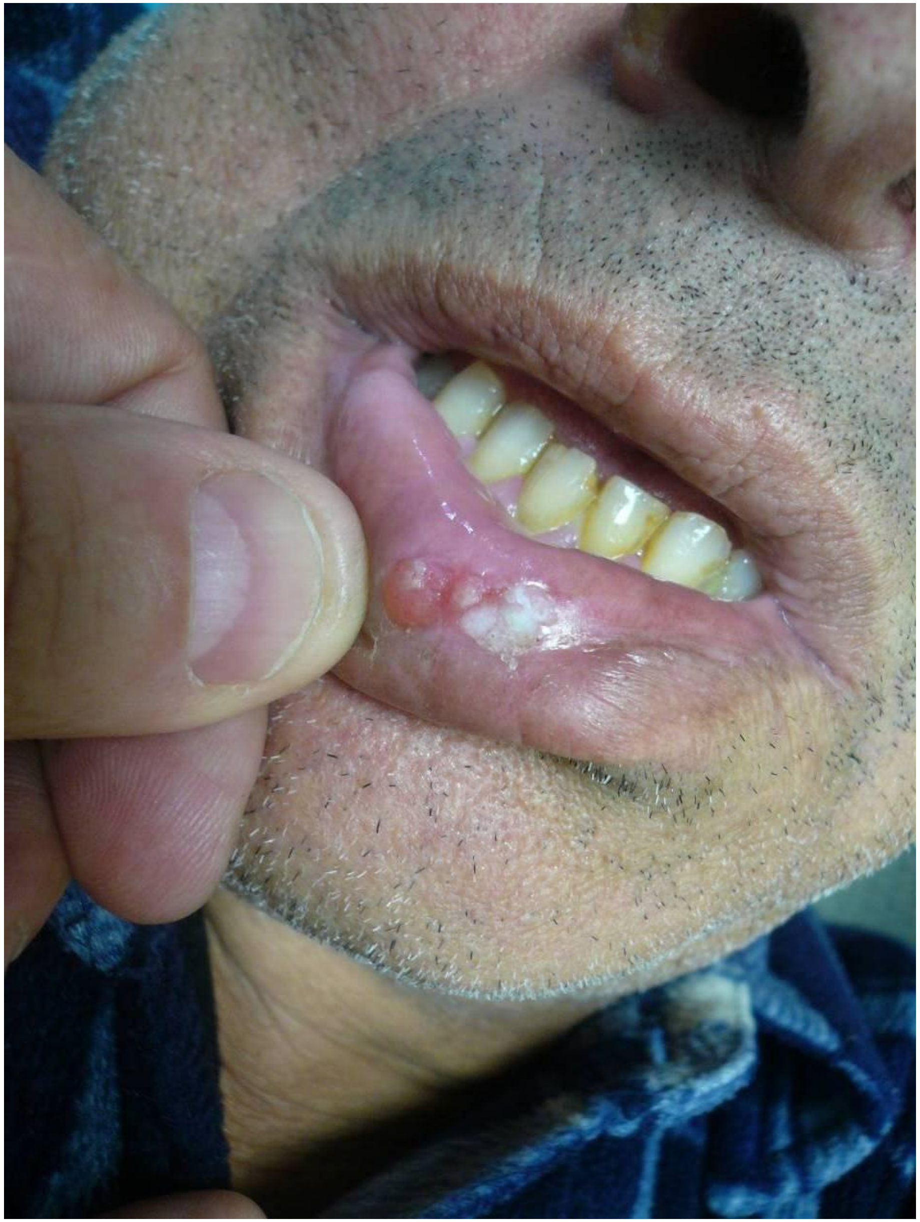

Male, 48-years. Heavy smoker++
RA treated with steroids and methotrexate for 12 years. Asymptomatic lesion of the lip for 3 months. No palpable lymph node.

A. Benign
B. Pre-malignant or malignant

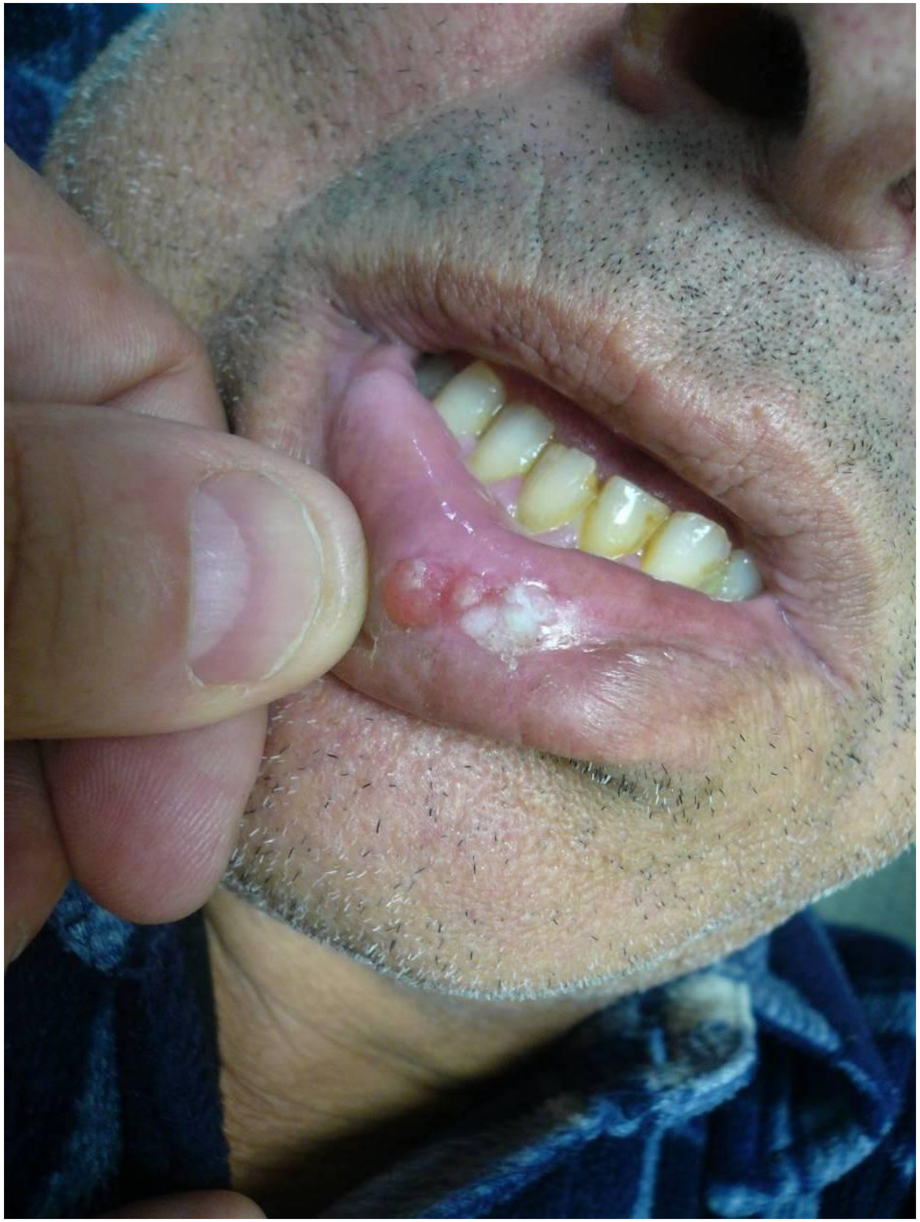

Male, 48-years. Heavy smoker++
RA treated with steroids and methotrexate for 12 years. Asymptomatic lesion of the lip for 3 months. No palpable lymph node.

What is your diagnosis?:

A- Aphtous ulcer
B- Oral diapneusia (lip sucking habit)
C- Squamous cell carcinoma
D- Reccurent herpes labialis
E- Achromic melanoma

Female, 47-years, RA treated with etanercept for 2 years.
Very anxious about a pigmented lesion of the breast that she did not notice before.

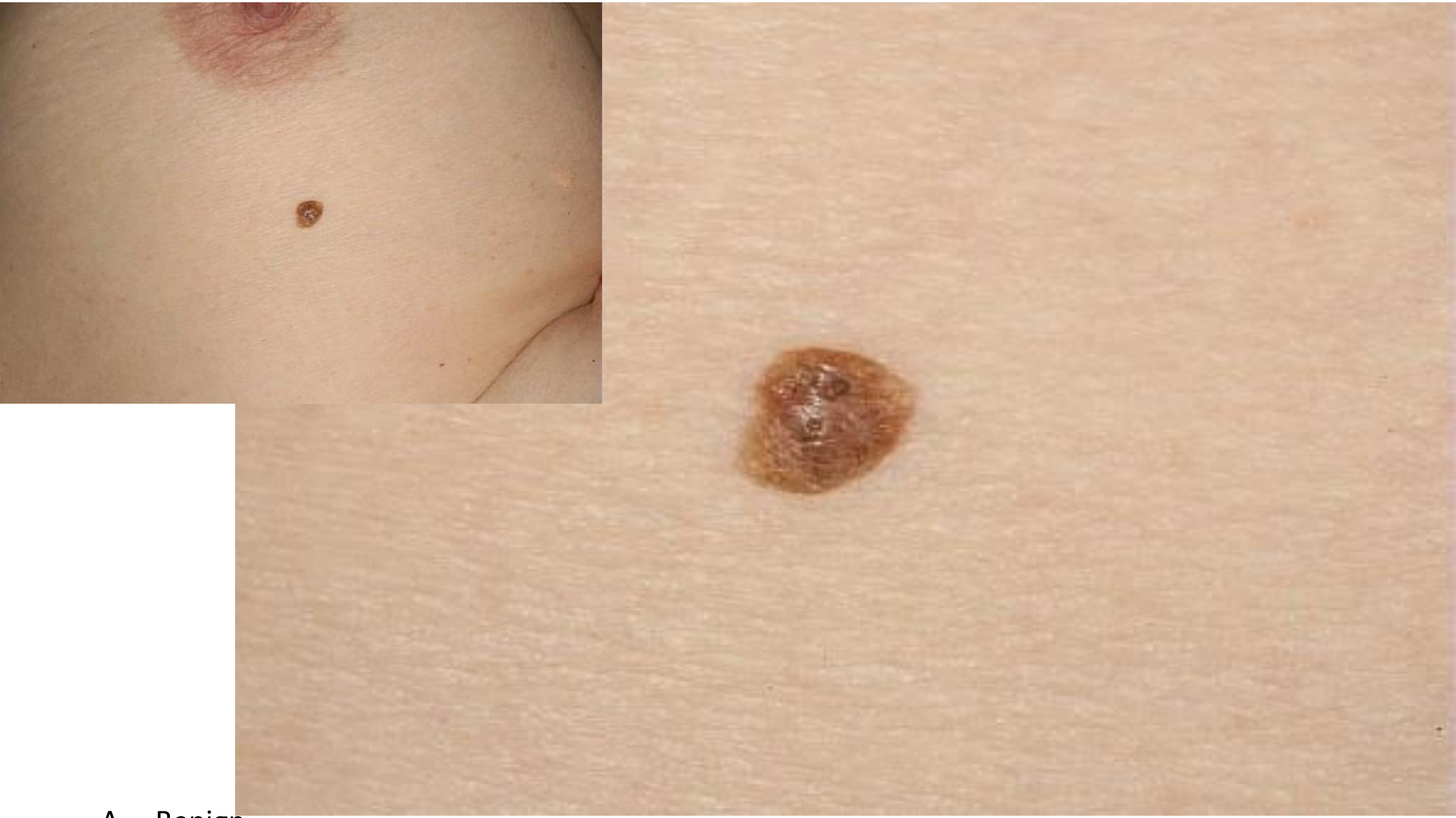

A. Benign
B. Pre-malignant or malignant

Female, 47-years, RA treated with etanercept for 2 years.
Very anxious about a pigmented lesion of the breast that she did not notice before.

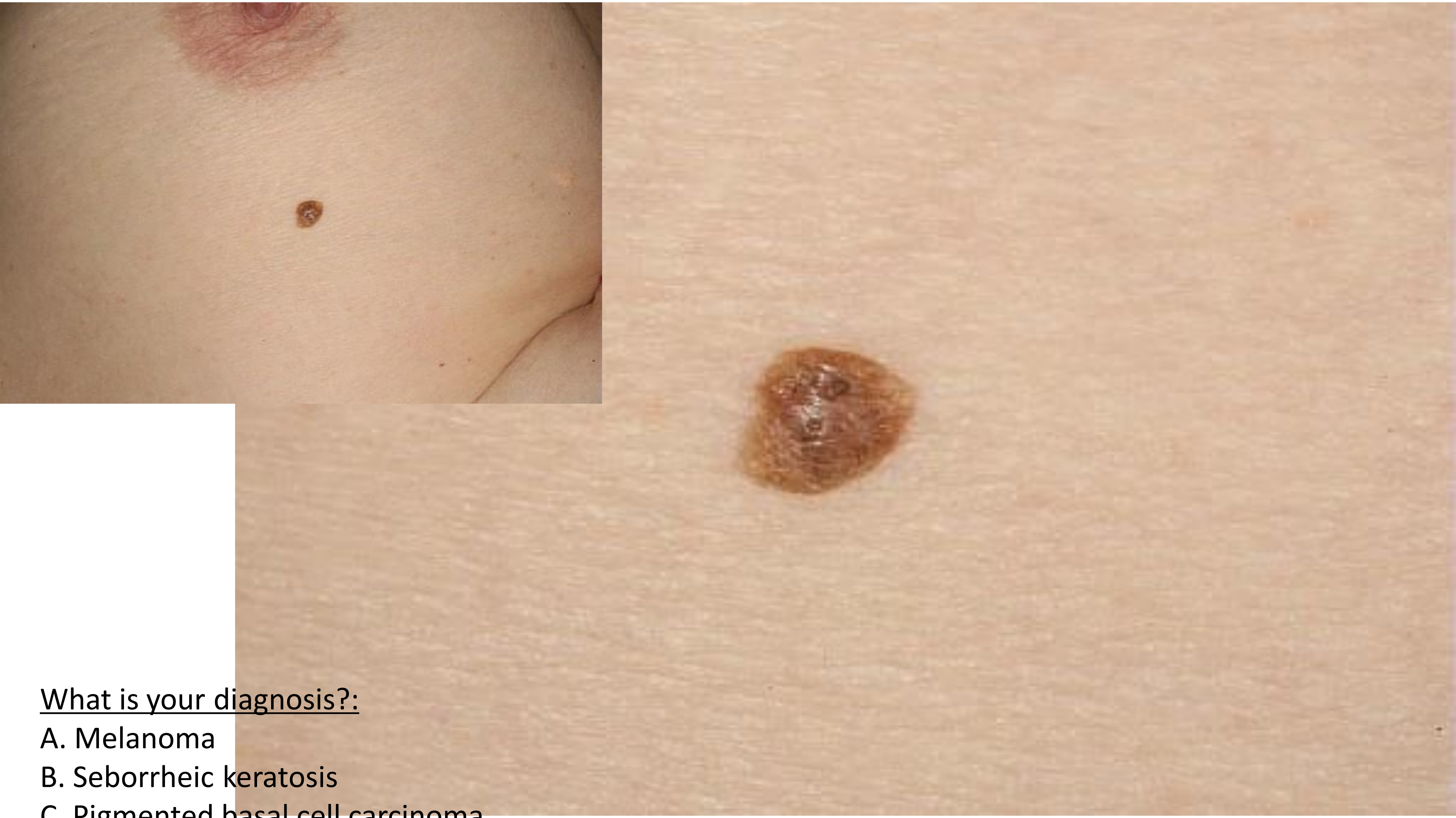

<u>What is your diagnosis?:</u>

A. Melanoma
B. Seborrheic keratosis
C. Pigmented basal cell carcinoma
D. Naevus
E. Dermal naevus

Male, 80-years, past history of right hemi thoracic melanoma with LN involvement, in remission for 5 years.
Asymptomatic skin lesion of the back (left shoulder), soft consistance.

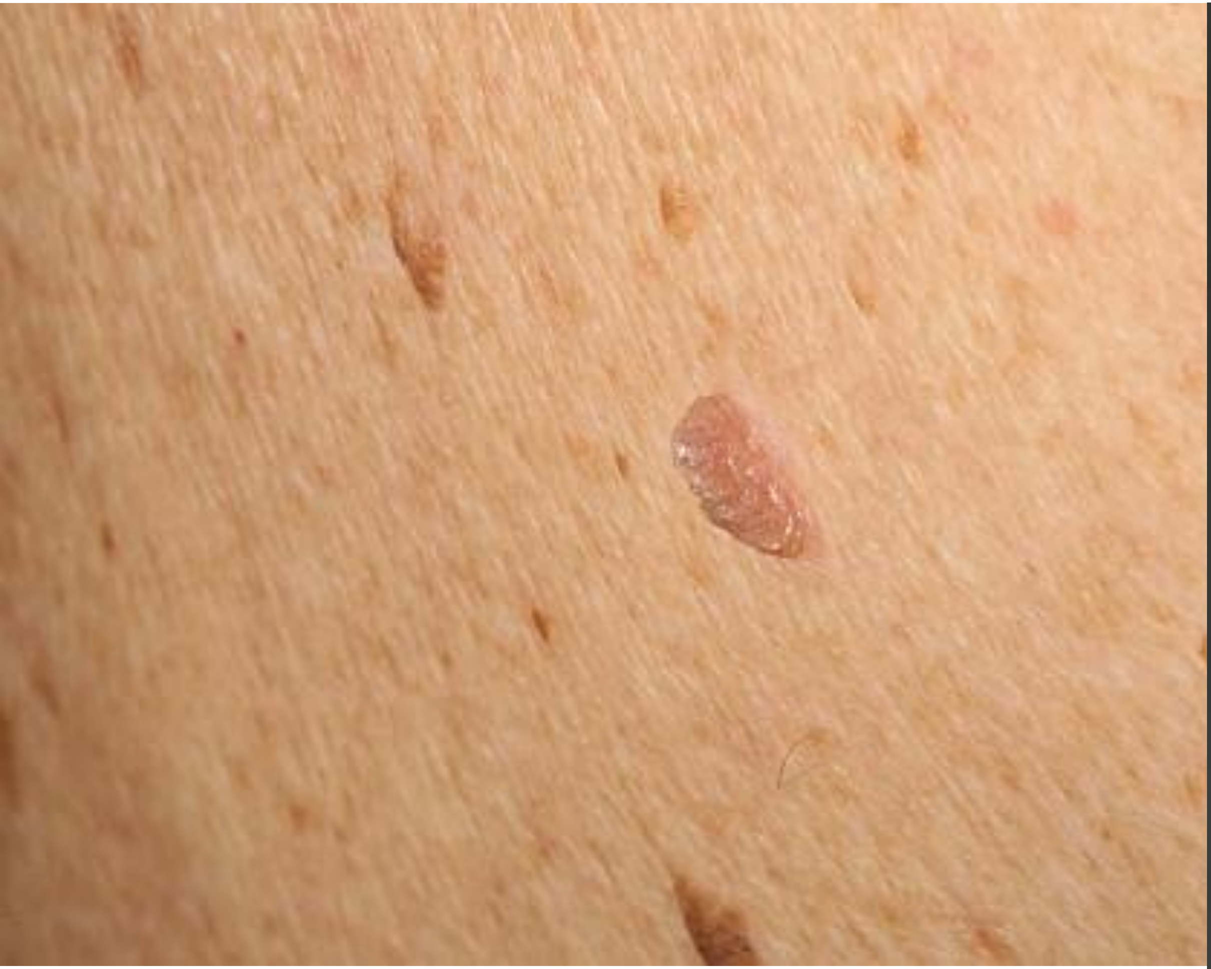

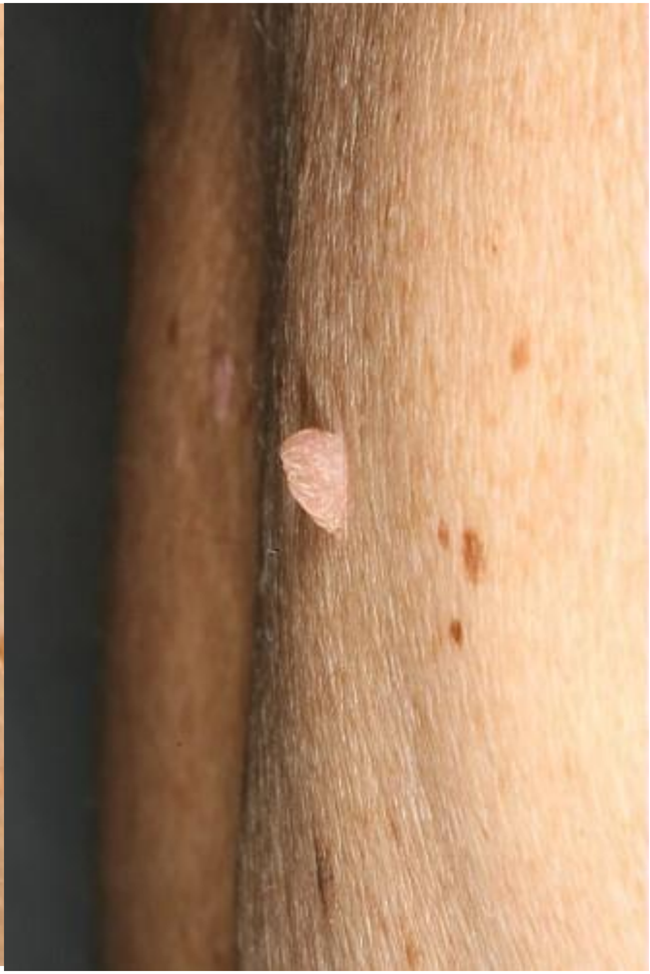

A. Benign
B. Pre-malignant or malignant

Male, 80-years, past history of right hemi thoracic melanoma with LN involvement, in remission for 5 years.
Asymptomatic skin lesion of the back (left shoulder), soft consistance.

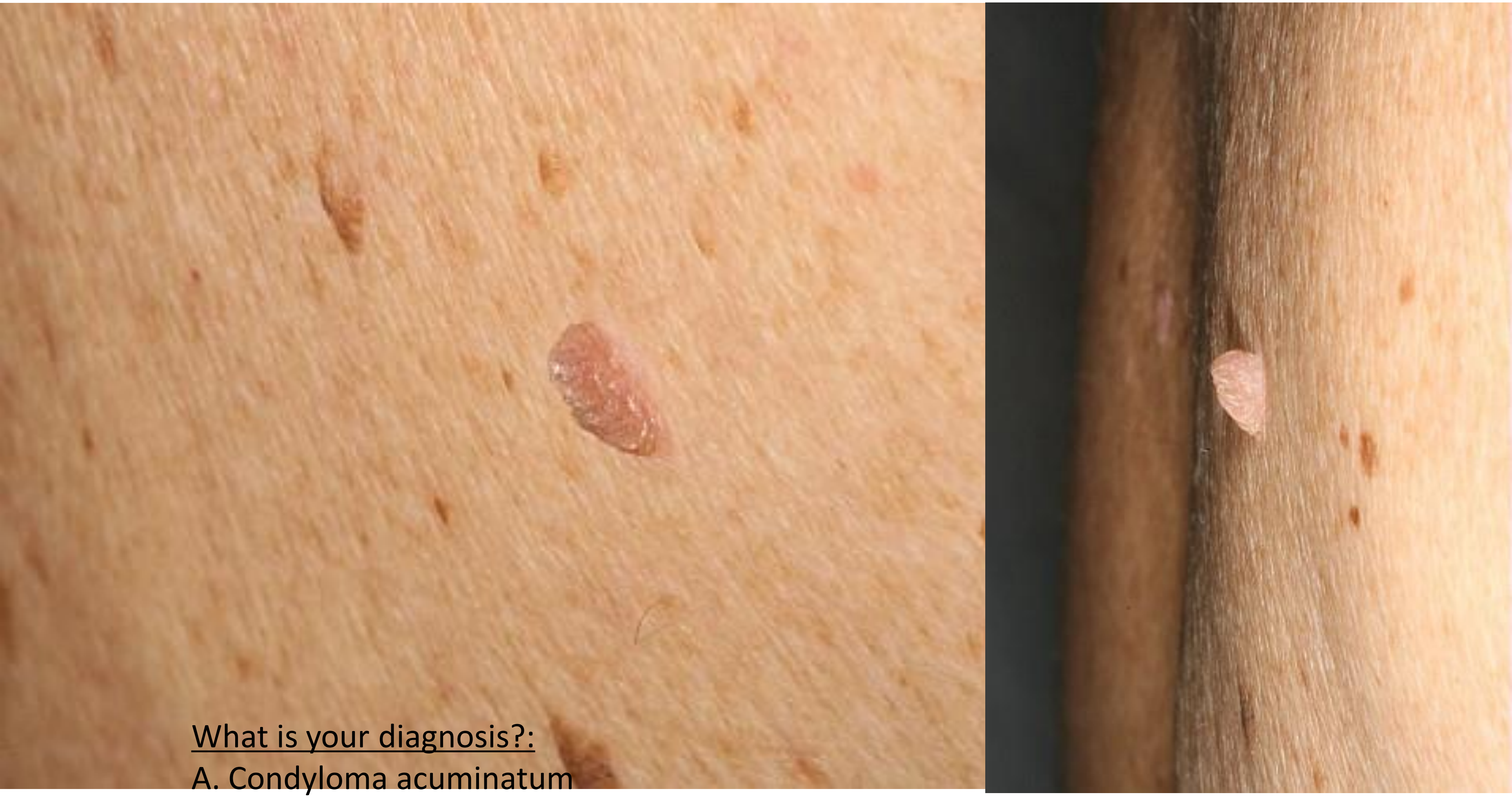

<u>What is your diagnosis?:</u>

A. Condyloma acuminatum
B. Seborrheic keratosis
C. Molluscum pendulum (acrochordon)
D. Actinic keratosis
E. Dermal naevus

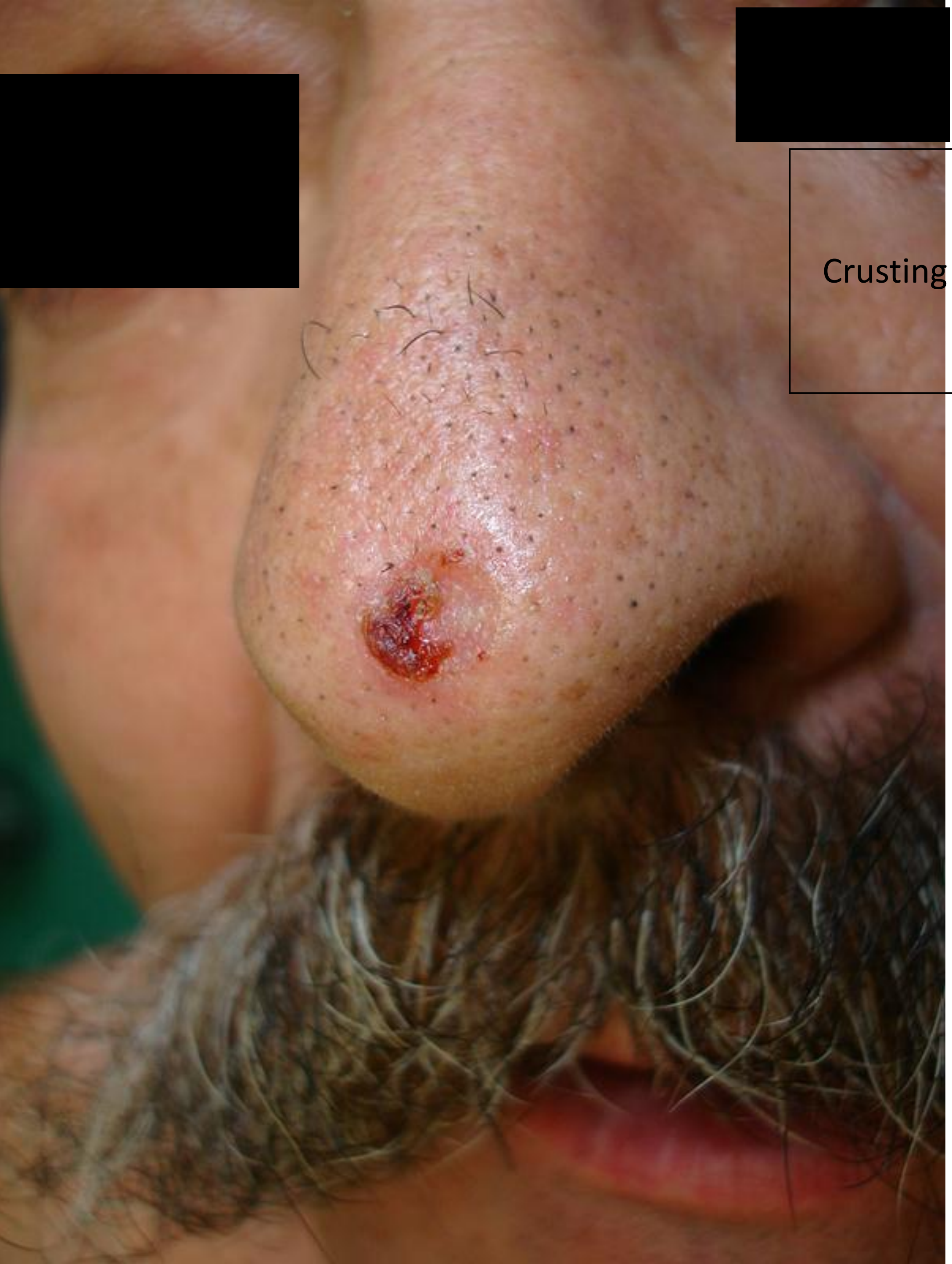

Male, 48-years.
Diabetes mellitus
Crusting cutaneous lesion on the nose for 12 months.
No pain, no itch.
Occasional bleeding

A. Benign
B. Pre-malignant or malignant

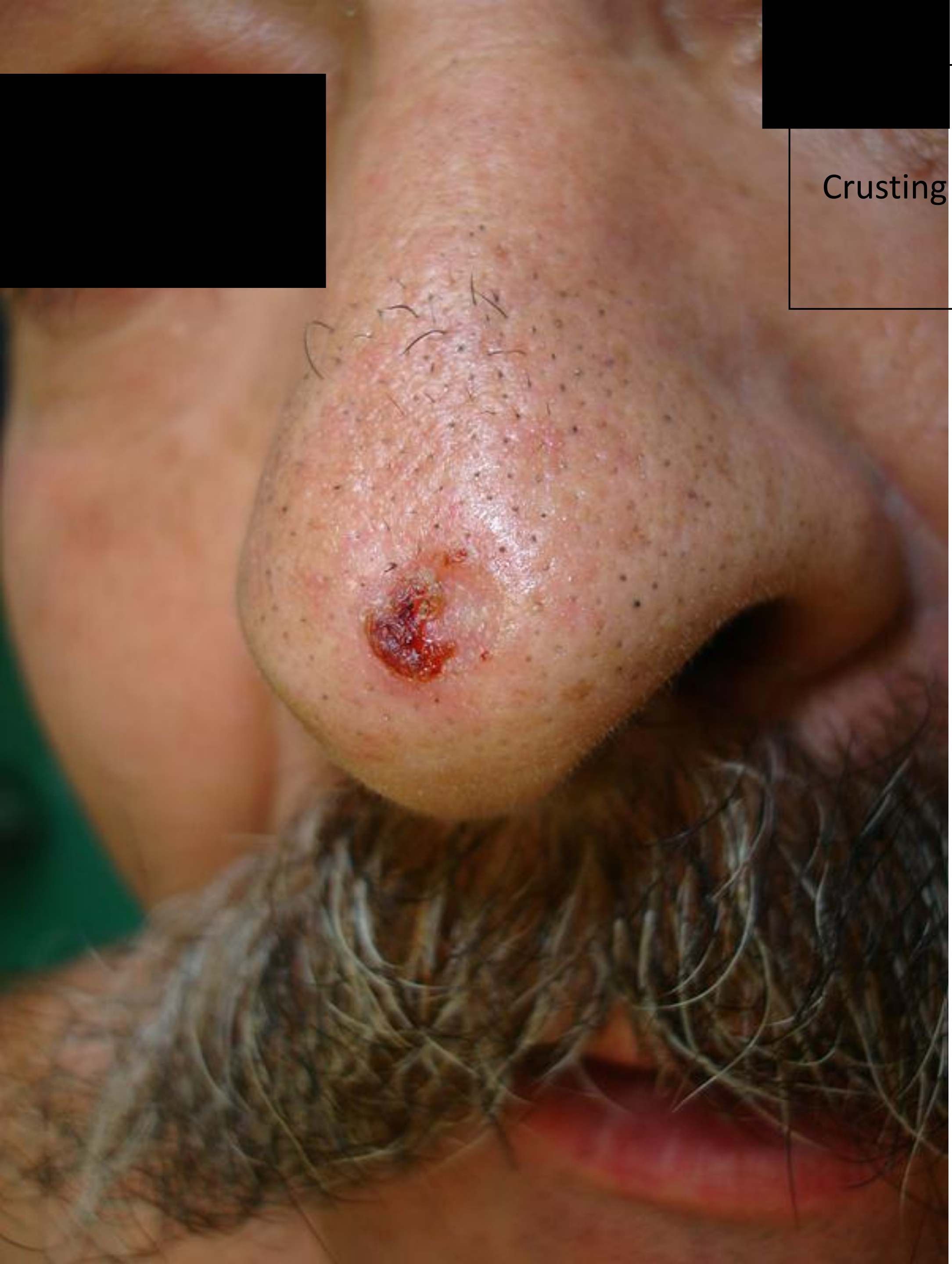

Male, 48-years.
Diabetes mellitus
Crusting cutaneous lesion on the nose for 12 months.
No pain, no itch.
Occasional bleeding

<u>What is your diagnosis?:</u>

A- Actinic keratosis
B- Squamous cell carcinoma
C- Leishmaniosis
D- Pathomimia
E- Recurrent herpes

Female, 35-years. Psoriasis treated with phototherapy for 12 years.
Labial lesion for 6 months. Symptoms-free.

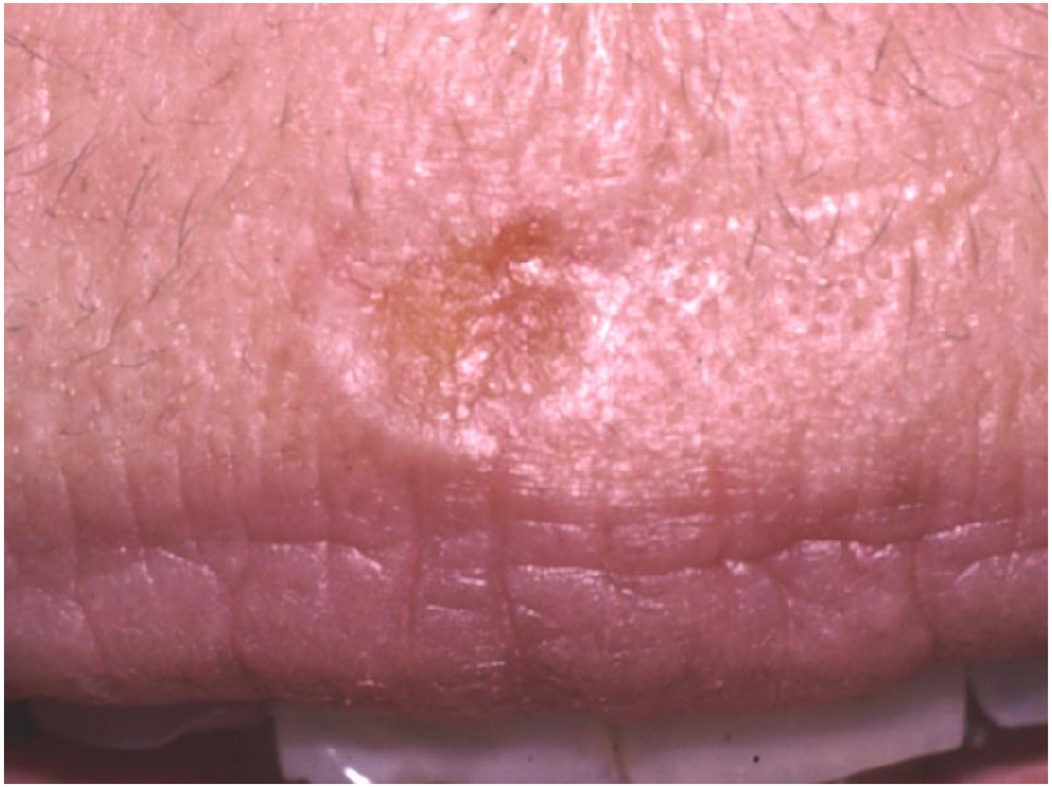

A. Benign
B. Pre-malignant or malignant

Female, 35-years. Psoriasis treated with phototherapy for 12 years.
Labial lesion for 6 months. Symptoms-free.

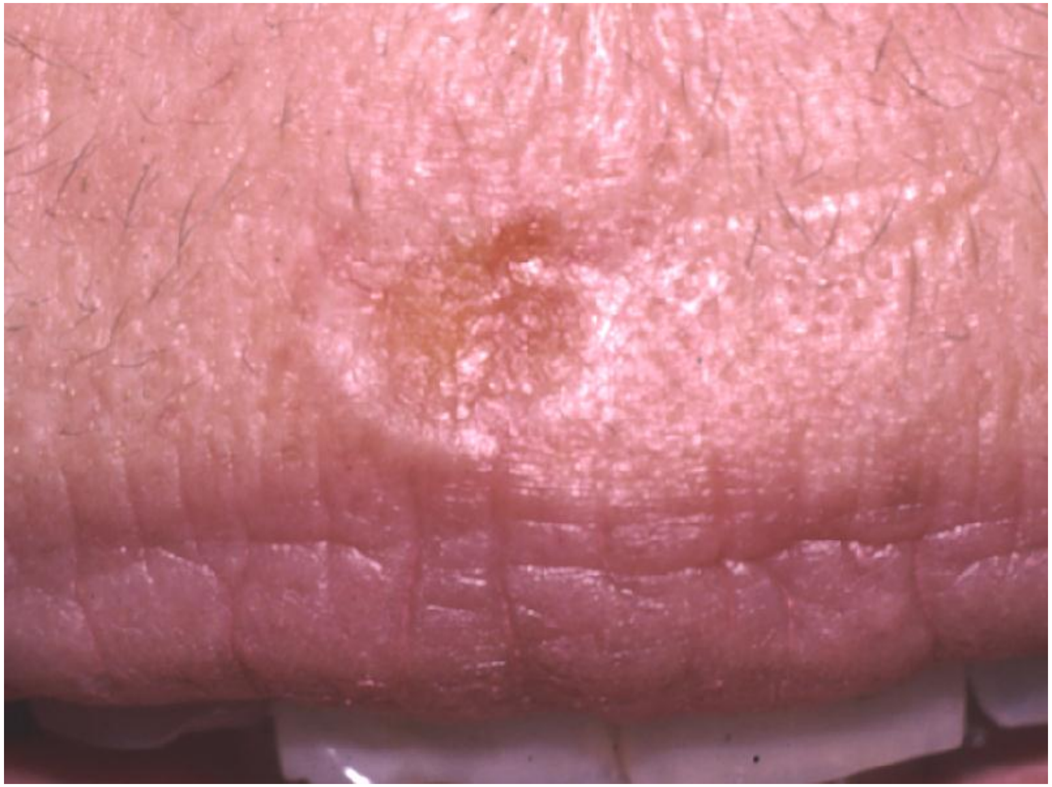

<u>What is your diagnosis?:</u>

A- Cheloid scar

B- Basal cell carcinoma

C- Dermatophytosis

D- Granuloma annulare

E- Lesion secondary to lip sucking habit

Male, 50-years. Rapid modification of a sub cutaneous lesion of the back, initially of small size, unpainful and with a strong consistance .
Increased size, painful, soft consistance .
Topical application of eosin.

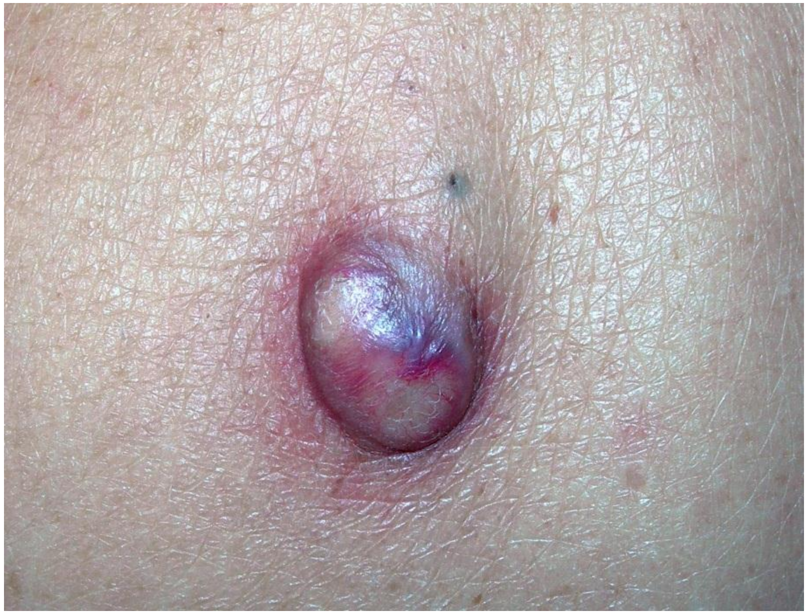

A. Benign
B. Pre-malignant or malignant

Male, 50-years. Rapid modification of a sub cutaneous lesion of the back, initially of small size, unpainful and with a strong consistance .
Increased size, painful, soft consistance .
Topical application of eosin.

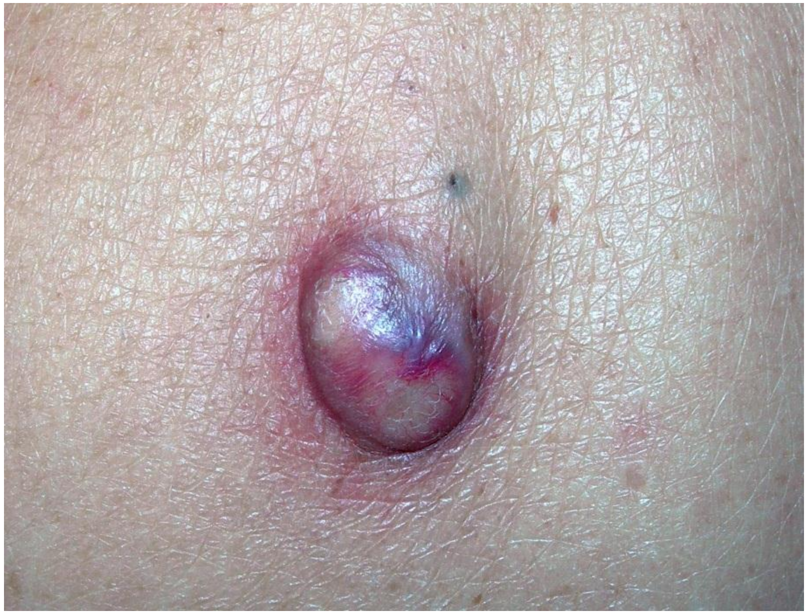

<u>What is your diagnosis?:</u>

A. Lipoma
B. Liposarcoma
C. Infected cyst
D. Nodular melanoma
E. Lymphoma

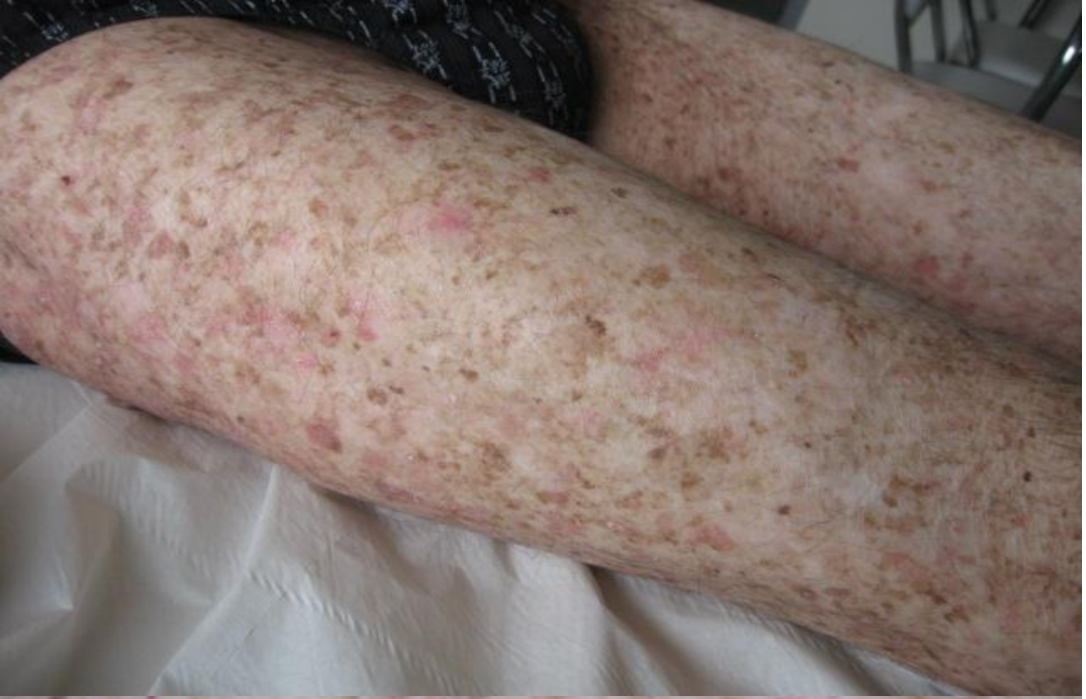

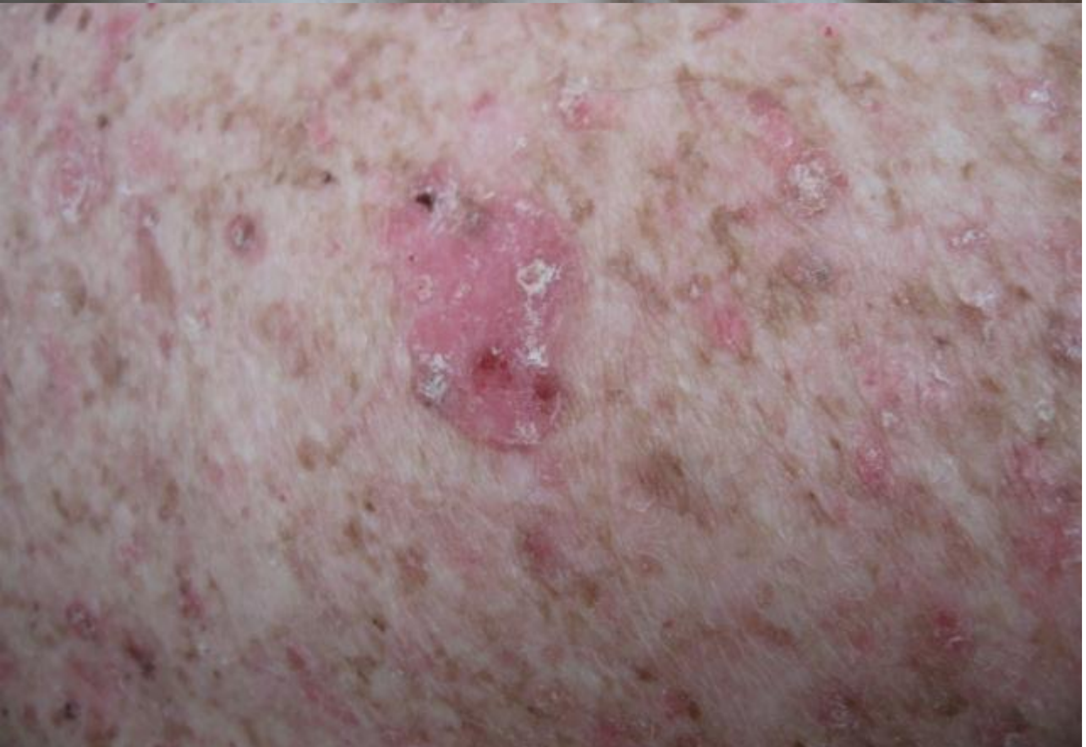

Male, 55-years. Psoriasis and psoriatic arthritis.
Previous treatments with PUVA and acitretin
(> 200 sessions).
Start of methotrexate 20 mg/ week for 3 months.
Rapid modification of skin lesions, disparition of Arthralgia.
Multiple erythematous skin lesions.
No itch.

A. Benign
B. Pre-malignant or malignant

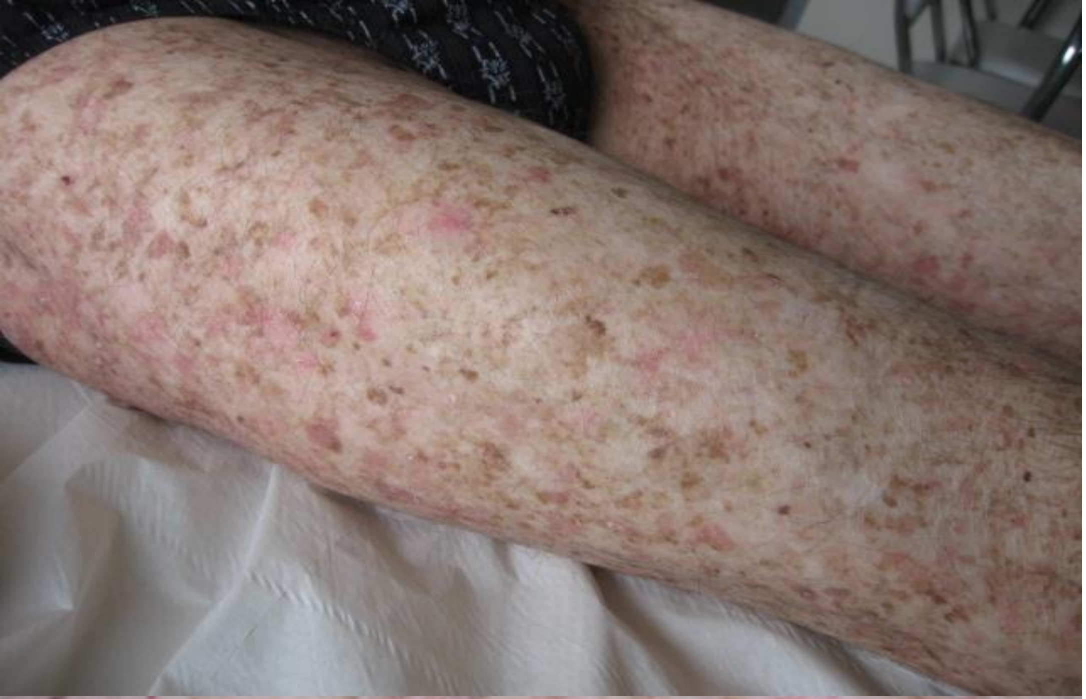

Male, 55-years. Psoriasis and psoriatic arthritis.
Previous treatments with PUVA and acitretin
(> 200 sessions).
Start of methotrexate 20 mg/ week for 3 months.
Rapid modification of skin lesions, disparition of
Arthralgia.
Multiple erythematous skin lesions.
No itch.

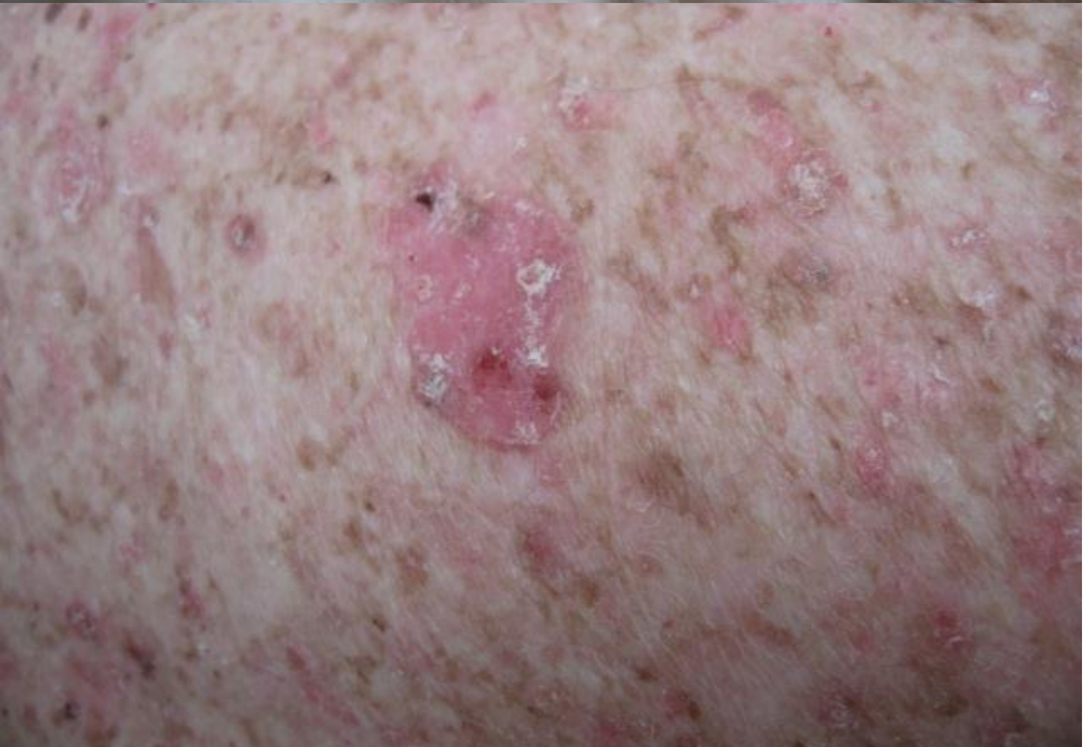

<u>What is your diagnosis?:</u>

A. Residual psoriasis vulgaris
B. Paradoxical psoriasis
C. Cutaneous lymphoma induced by methotrexate
D. Multiple actinic keratosis and helioderma
E. Cutaneous drug reaction to methotrexate

Male, 80-years, history of melanoma of the face. Pigmented lesion of the back, small size (2-3 mm), in relief. Symptoms free. No palpable lymph node.

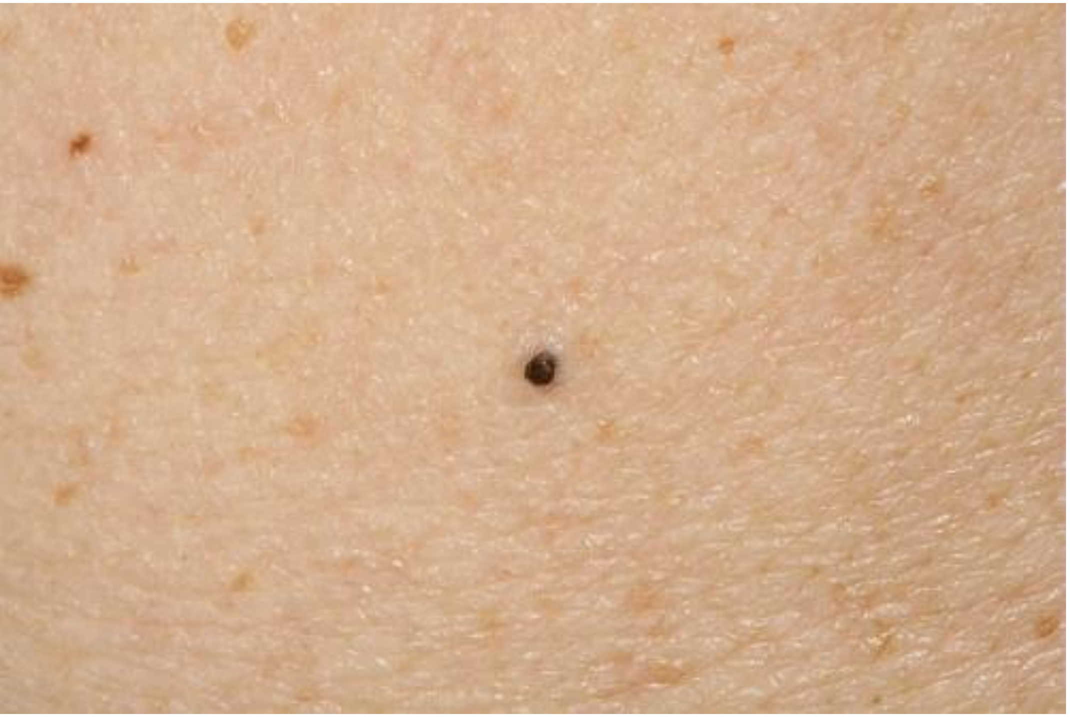

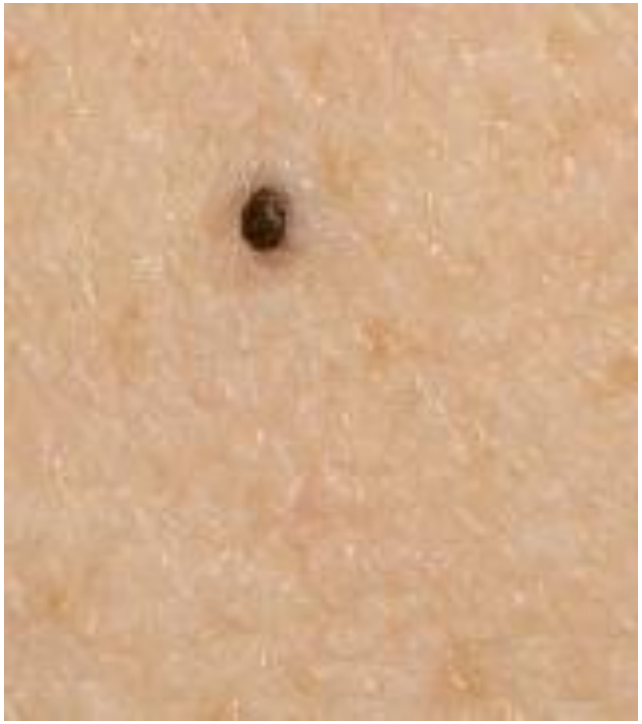

A. Benign
B. Pre-malignant or malignant

Male, 80-years, history of melanoma of the face. Pigmented lesion of the back, small size (2-3 mm), in relief. Symptoms free. No palpable lymph node.

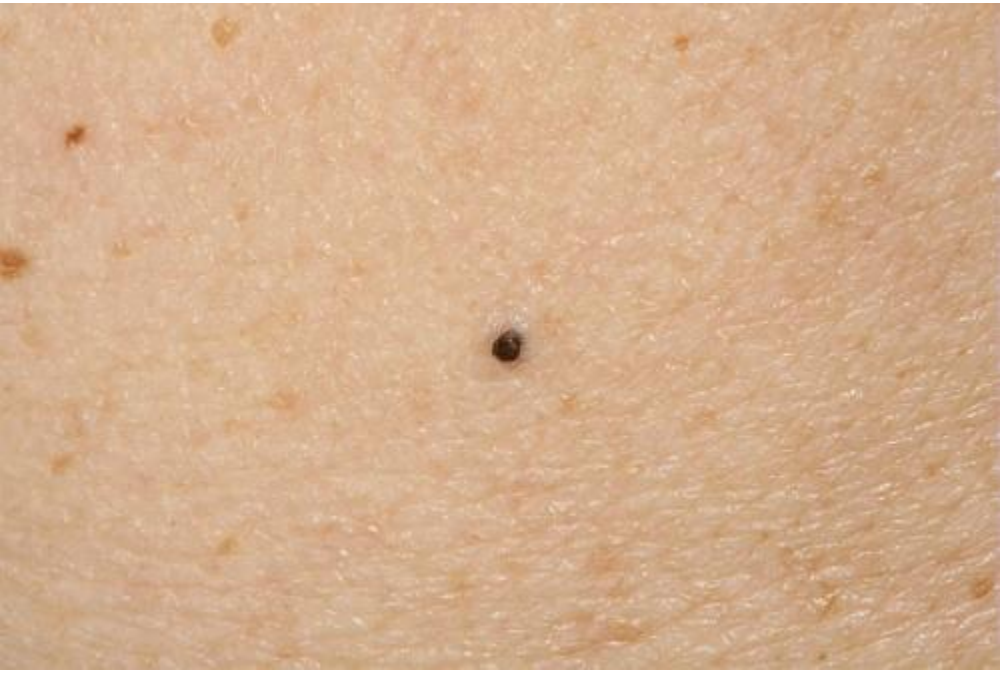

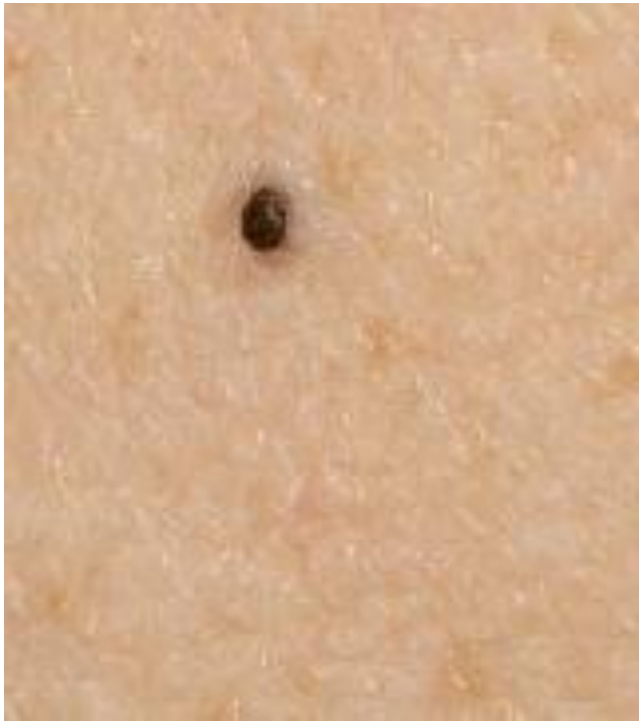

What is your diagnosis?:

A. A new primary cutaneous melanoma

B. Melanoma cutaneous metastasis

C. Pigmented basal cell carcinoma

D. Comedone

E. Dermatofibroma

Female, 35-years, AS treated with etanercept for 2 years.
Pigmented skin lesion in relief with strong consistance (arm).
No palpable axillary lymph node.

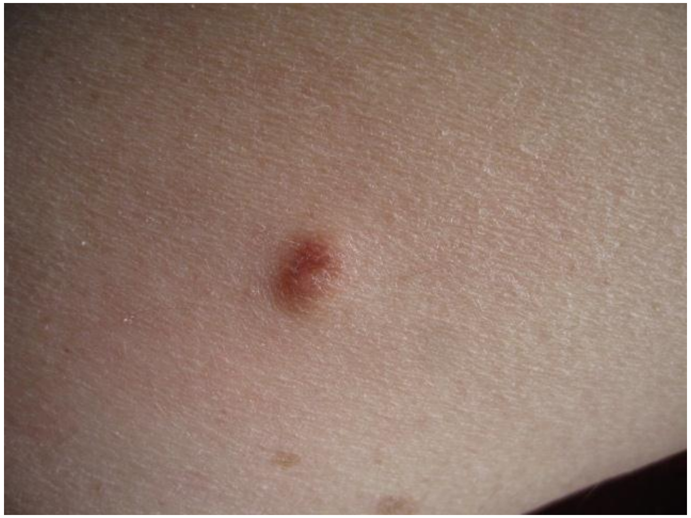

A. Benign
B. Pre-malignant or malignant

Female, 35-years, AS treated with etanercept for 2 years.
Pigmented skin lesion in relief with strong consistance (arm).
No palpable axillary lymph node.

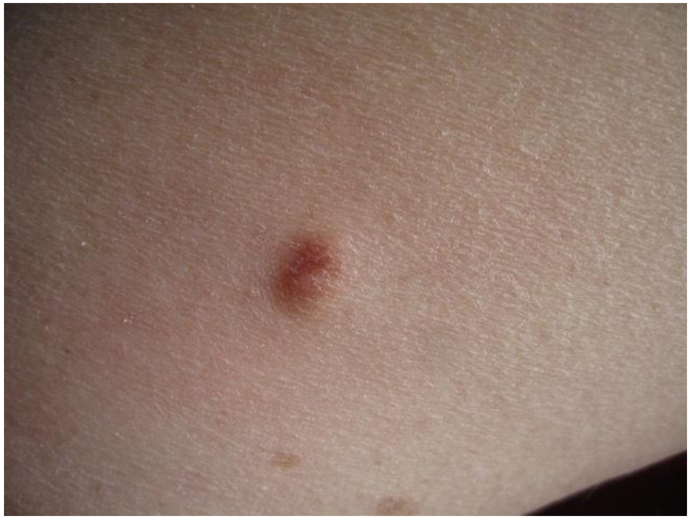

<u>What is your diagnosis?:</u>

A. Cutaneous sarcoma
B. Melanoma
C. Dermal naevus
D. Dermatofibroma
E. Pigmented basal cell carcinoma

Male, 55-years. RA treated with MTX for 12 years + uncontrolled diabetes mellitus.
Heel lesion for 6 months.
No history of trauma.
Symptom-free.
Failure of topical treatment.

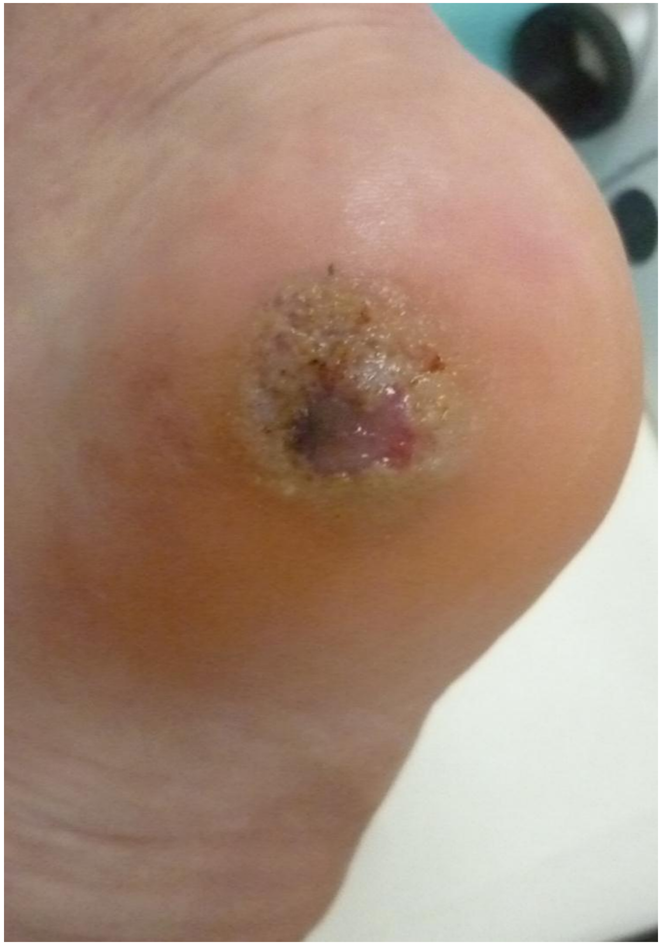

A. Benign
B. Pre-malignant or malignant

Male, 55-years. RA treated with MTX for 12 years + uncontrolled diabetes mellitus.
Heel lesion for 6 months.
No history of trauma.
Symptom-free.
Failure of topical treatment.

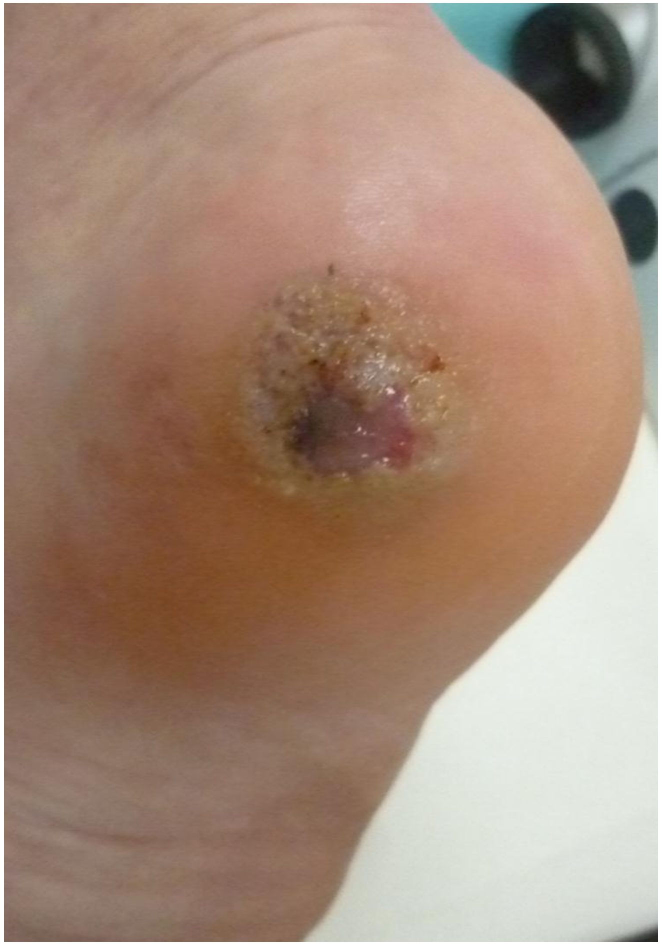

What is your diagnosis?:

A- Diabetic foot ulcer
B- Basal cell carcinoma
C- Contact dermatitis
D- Melanoma
E- Wart

Female, 74-years, with diabetes. Several rough skin lesions of the back of the hands. No pain, no itch. No palm lesions. Similar lesions on the forehead and the nose. No arthralgia, no myalgia.

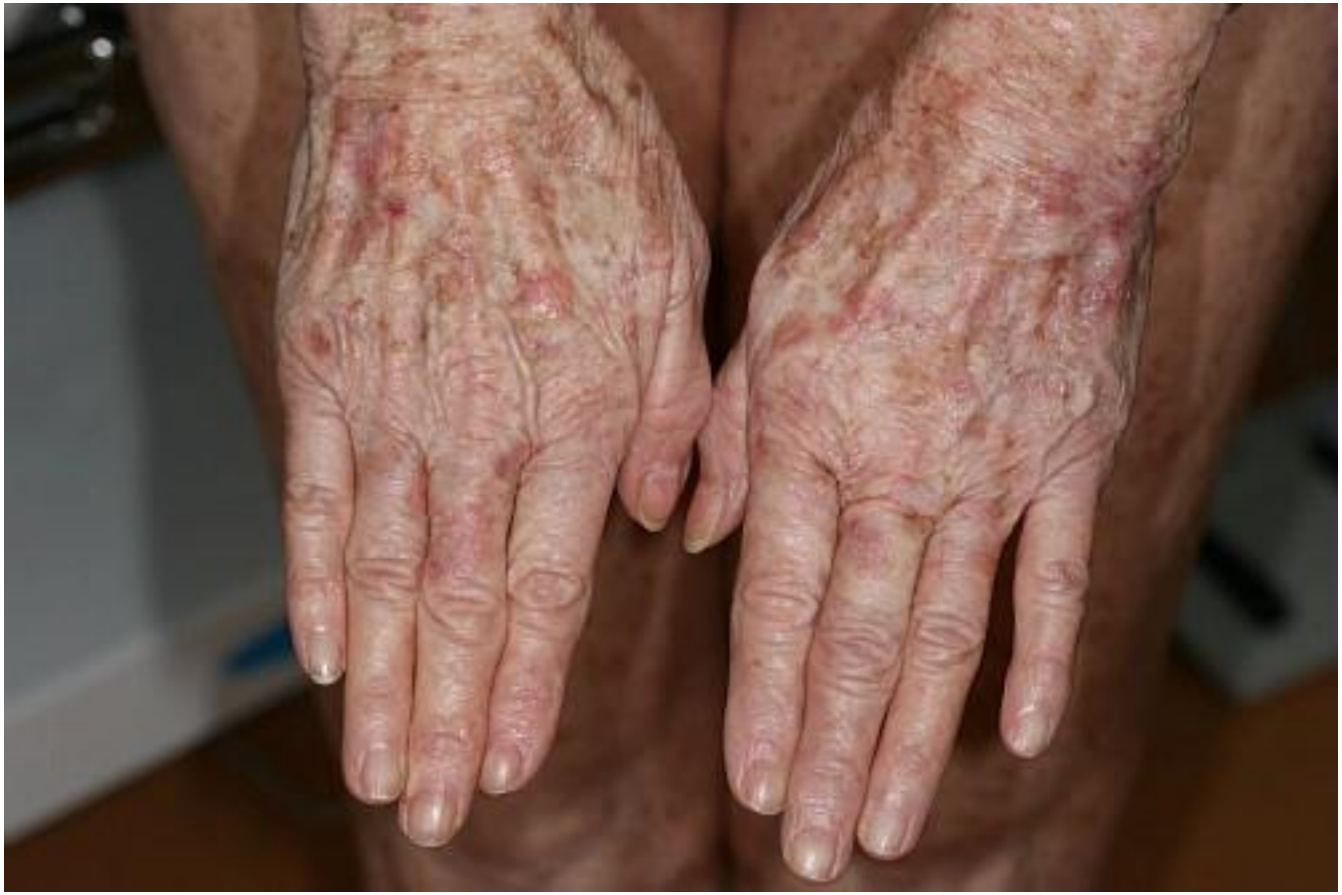

A. Benign
B. Pre-malignant or malignant

Female, 74-years, with diabetes. Several rough skin lesions of the back of the hands. No pain, no itch. No palm lesions. Similar lesions on the forehead and the nose. No arthralgia, no myalgia.

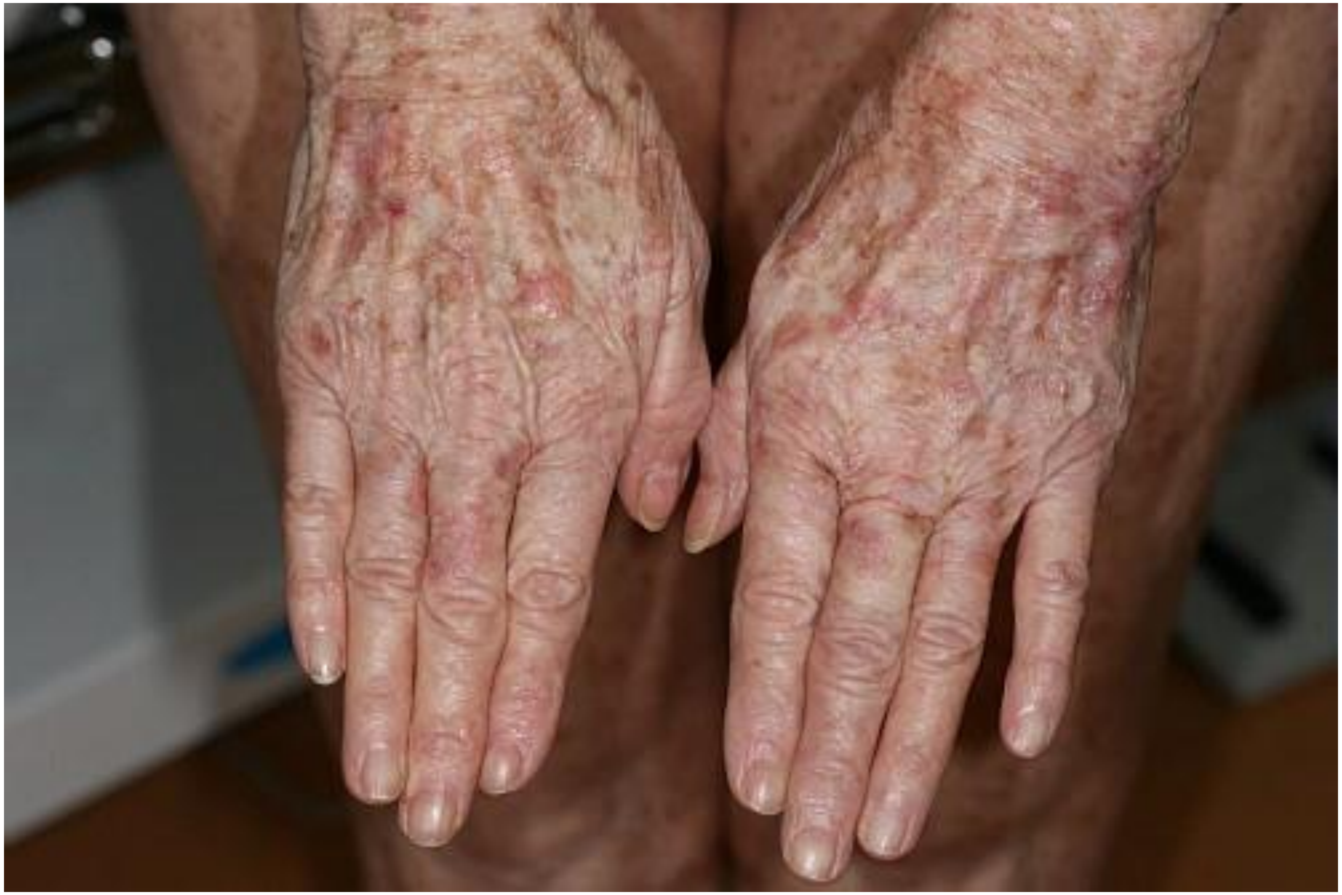

<u>What is your diagnosis?:</u>

A. Amyopathic dermatomyositis
B. Discoid cutaneous lupus
C. Contact dermatitis
D. Actinic keratosis
E. Dermatophytosis

Skin lesion of the leg in a 74-year-old female. No pain, no itch. Evoluting for 6 months with slow increase.

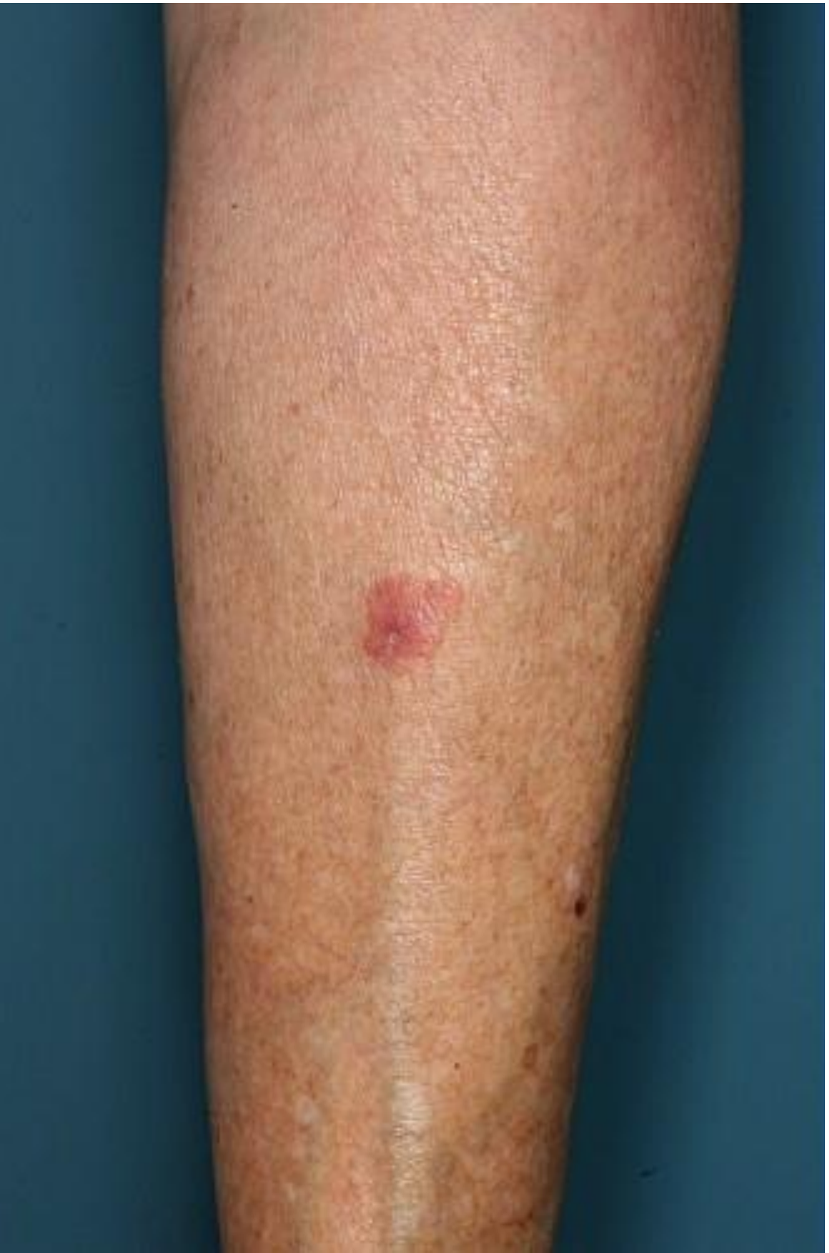

A. Benign
B. Pre-malignant or malignant

Skin lesion of the leg in a 74-year-old female. No pain, no itch. Evoluting for 6 months with slow increase.

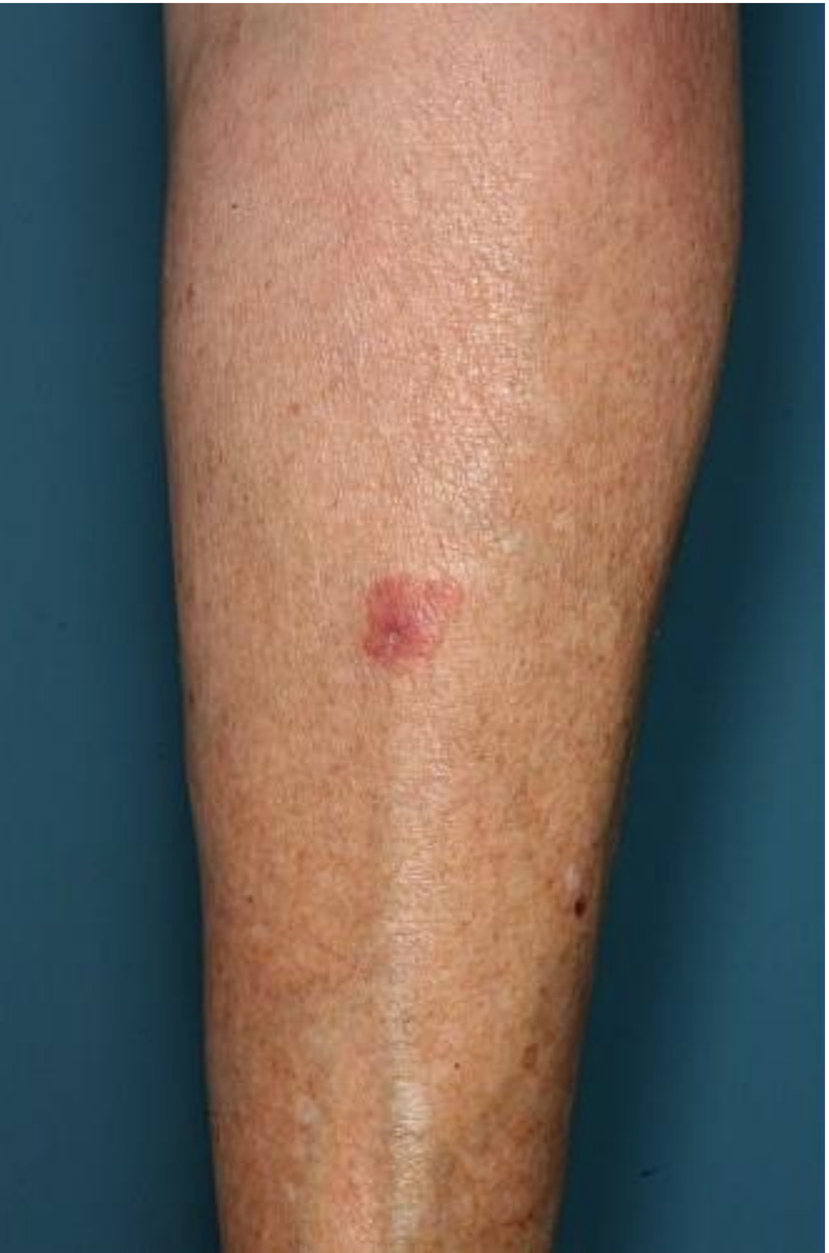

What is your diagnosis? :

A. Dermatophytosis
B. Psoriasis vulgaris
C. Nummular eczema
D. Bowen’s disease
E. Kaposi’s sarcoma

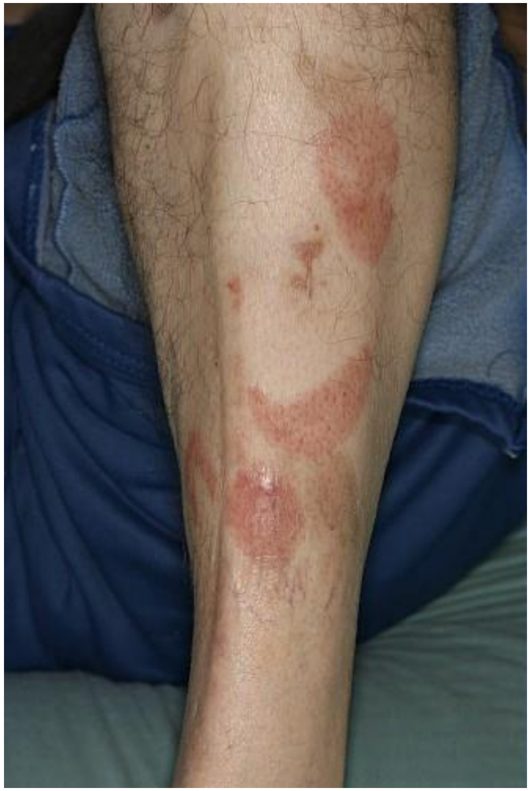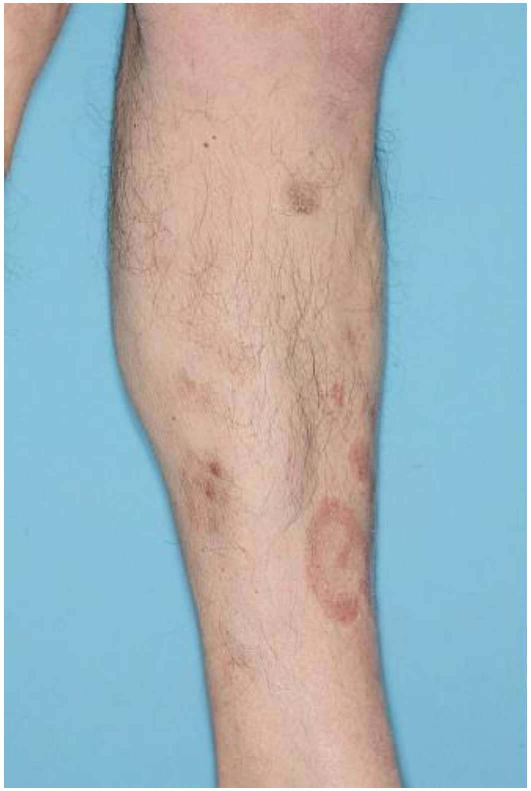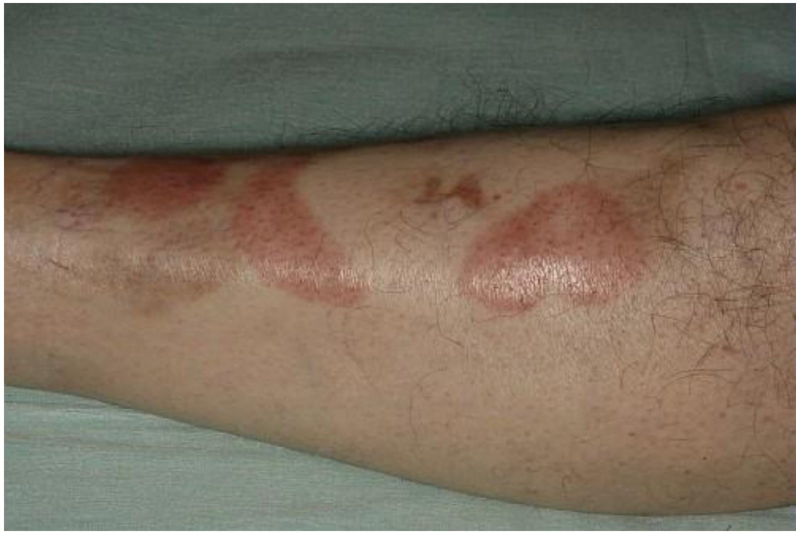

Male, 55-years old. RA in remission with adalimumab for 2 years + uncontrolled diabetes mellitus.
Travel during 15 days in Mexico, 8 months ago.
Legs skin lesions for 6 months, with progressive extension. Slightly pruriginous.

A. Benign
B. Pre-malignant or malignant

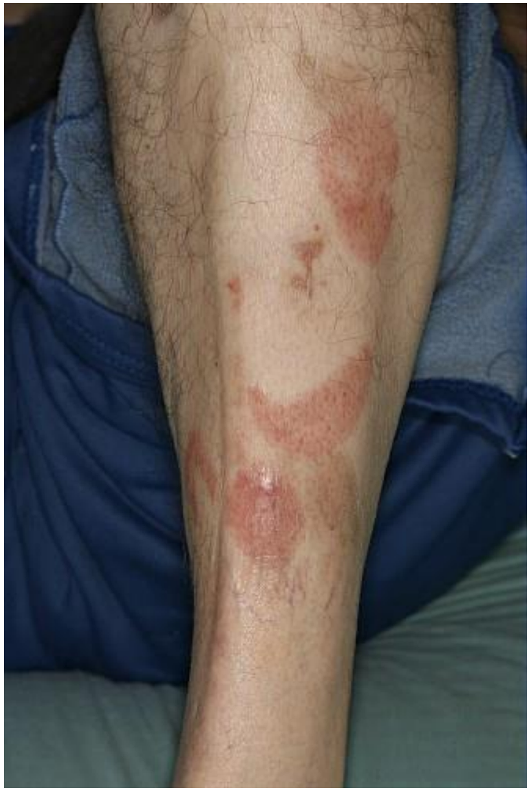

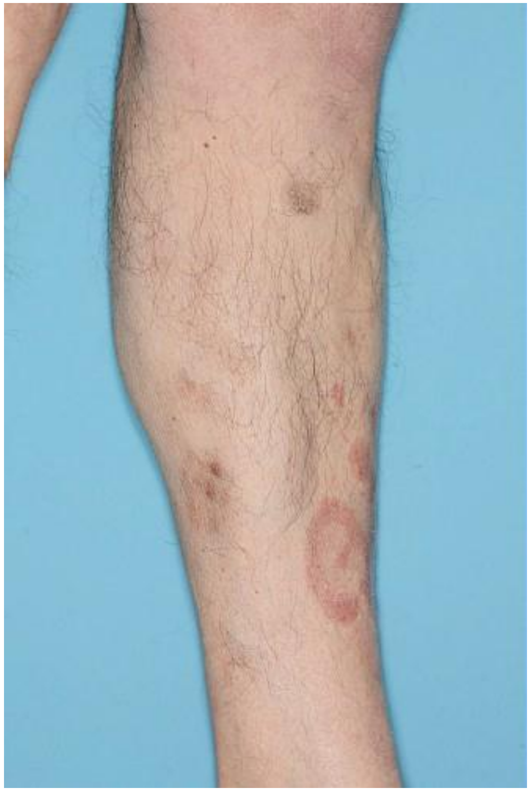

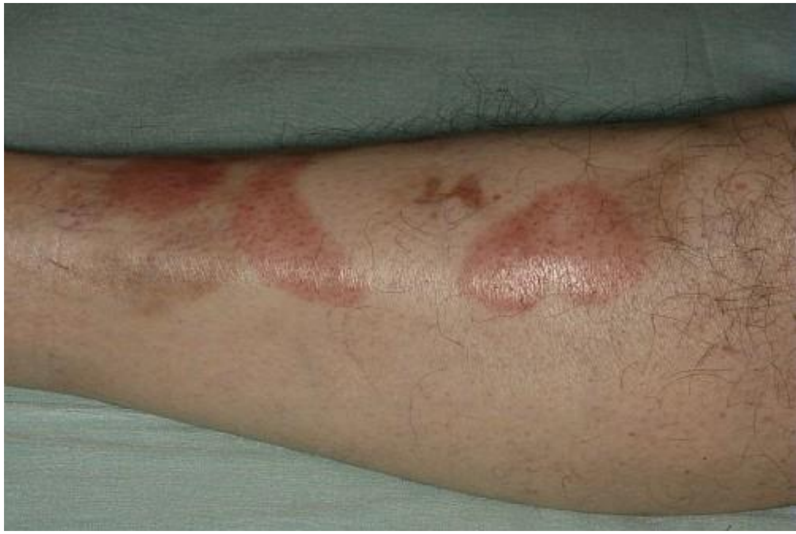

Male, 55-years old. RA in remission with adalimumab for 2 years + uncontrolled diabetes mellitus.
Travel during 15 days in Mexico, 8 months ago.
Legs skin lesions for 6 months, with progressive extension. Slightly pruriginous.

<u>What is your diagnosis?:</u>

A. Psoriasis
B. Dermatophytosis
C. Erythema nodosum
D. Kaposi's sarcoma
E. Cutaneous lymphoma

Male, 44-years old. Psoriasis treated with phototherapy for 12 years.
Nail lesion, slightly painful, for 15 days.
History of local trauma 2 weeks ago.

A. Benign
B. Pre-malignant or malignant

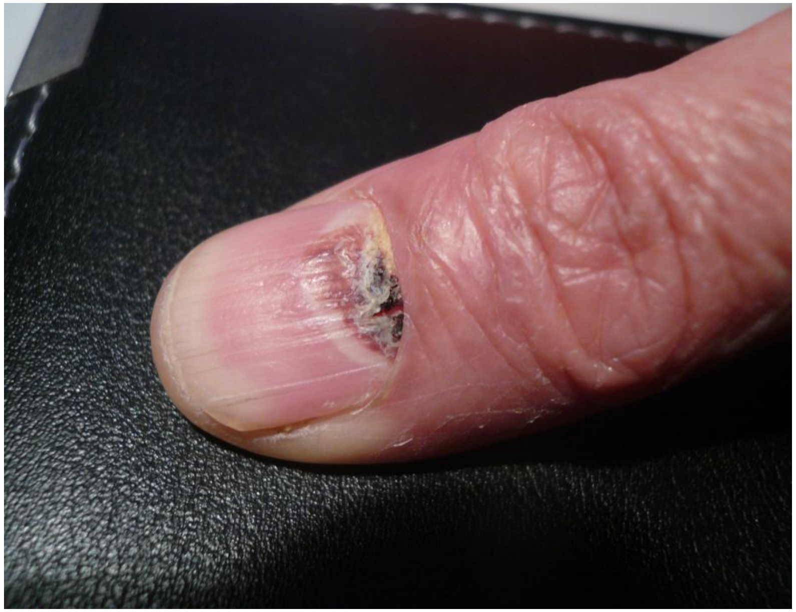

Male, 44-years old. Psoriasis treated with phototherapy for 12 years.
Nail lesion, slightly painful, for 15 days.
History of local trauma 2 weeks ago.

<u>What is your diagnosis?:</u>

A- Nail melanoma
B- Sub-ungual haematoma
C- Nail psoriasis
D- Nail Lichen planus
E- Sub-ungual exostosis

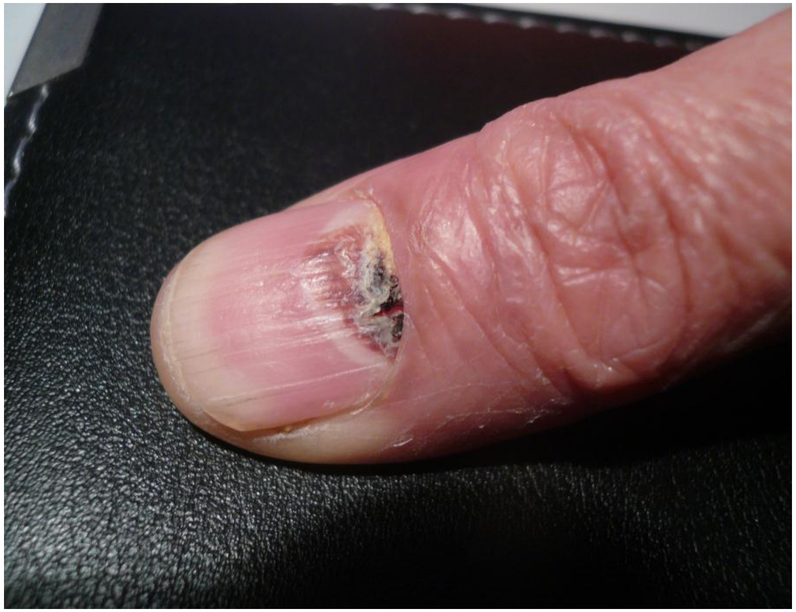

Supplement: S1 File — (PDF) [file pone.0127564.s001.pdf]
