# Supplementary material for: Online Training on Skin Cancer Diagnosis in Rheumatologists: Results from a Nationwide Randomized Web-Based Survey
Source: PLoS One. 2015 May 21;10(5):e0127564. doi: 10.1371/journal.pone.0127564 (PMC4440619; doi:10.1371/journal.pone.0127564)
Supplement: S1 Text — (PDF) [file pone.0127564.s002.pdf]

N° de rhumatologue :

Date de J0 : …./……../………
(jour / mois / année)

➢ DONNEES DEMOGRAPHIQUES DU RHUMATOLOGUE

Sexe ☐ M ☐ F

Ancienneté de la pratique : ……ans

Age ans

Type d'exercice
- Libéral ☐
- Hospitalier ☐
- Mixte ☐
- Universitaire ☐

Localisation de l'activité : département _/_

➢ ACTIVITE

Population à prédominance Urbaine ☐ Rurale ☐ Mixte ☐

Nombre de patients atteint d'un rhumatisme inflammatoire vus par mois : _/_/_
Nombre de patients traités par biothérapie vus par mois : _/_/_

➢ CONSULTATION : chez les patients atteints de rhumatisme inflammatoire

A quelle fréquence voyez-vous un patient atteint de rhumatisme inflammatoire sous biothérapie ? :

☐ 1 mois ☐ 3 mois ☐ 6 mois ☐ Autre
Précisez : ______

	Systématiquement	Très souvent	Parfois	Peu souvent	Jamais
**Chez les patients atteints de rhumatisme inflammatoire, donnez-vous des conseils :**	☐	☐	☐	☐	☐
Pour le sevrage tabagique chez les fumeurs ?	☐	☐	☐	☐	☐
Nutrionnels ?	☐	☐	☐	☐	☐
De photoprotection ?					
**Recherchez-vous les facteurs de risque de cancer de la peau ?**	☐	☐	☐	☐	☐
Chez tous vos malades					
Chez les patients atteints de rhumatisme inflammatoire	☐	☐	☐	☐	☐
En cas de traitement par biothérapie	☐	☐	☐	☐	☐

N° de rhumatologue :

N° de rhumatologue :

➢ **CONSULTATION : Patients traités par BIOTHERAPIE**

**Avant de prescrire une biothérapie**					
Interrogez vous le patient sur l'apparition ou la modification de lésion cutanée ?	Systématiquement ☐	Très souvent ☐	Parfois ☐	Peu souvent ☐	Jamais ☐
Faites vous un examen cutané complet ?					
Seulement en cas de lésions signalées par le patient ☐	Systématiquement ☐	Très souvent ☐	Parfois ☐	Peu souvent ☐	Jamais ☐
Adressez vous le patient au dermatologue ?					
Seulement en cas de lésions signalées par le patient ☐	Systématiquement ☐	Très souvent ☐	Parfois ☐	Peu souvent ☐	Jamais ☐

**Lors de la consultation de suivi des patients sous biothérapie**					
Interrogez vous le patient sur l'apparition ou la modification de lésion cutanée ?	Systématiquement ☐	Très souvent ☐	Parfois ☐	Peu souvent ☐	Jamais ☐
Faites vous un examen cutané complet à chacune des consultations ?					
Seulement en cas de lésions signalées par le patient ☐	Systématiquement ☐	Très souvent ☐	Parfois ☐	Peu souvent ☐	Jamais ☐
Adressez vous vos patients chez le dermatologue une fois par an ?	Oui ☐	Non ☐	Autre précisez		
Laissez-vous le médecin traitant faire la surveillance cutanée ?	Oui ☐	Non ☐			

N° de rhumatologue :

➢ **Consultation : Patients atteints de rhumatisme inflammatoire non traités par biothérapie**

**Lors de la consultation de suivi des patients atteints de rhumatisme inflammatoire non traités par biothérapie**					
Interrogez vous le patient sur l'apparition ou la modification de lésion cutanée ?	Systématiquement ☐	Très souvent ☐	Parfois ☐	Peu souvent ☐	Jamais ☐
Faites vous un examen cutané complet à chacune des consultations ?					
Seulement en cas de lésions signalées par le patient ☐	Systématiquement ☐	Très souvent ☐	Parfois ☐	Peu souvent ☐	Jamais ☐
Adressez vous vos patients chez le dermatologue une fois par an ?	Oui ☐	Non ☐	Autre précisez		
Laissez-vous le médecin traitant faire la surveillance cutanée ?	Oui ☐	Non ☐			

N° de rhumatologue :

DEMOGRAPHIC CHARACTERISTICS OF PARTICIPATING RHEUMATOLOGIST

Sex ☐ M ☐ F

Years of practice : ……years

Age years

Practice modalities: Private ☐ Hospital ☐ Mixed ☐ University ☐

Localization of practice : zip code _/_

- **GENERAL PRACTICE**

Patients coming from City ☐ Countryside ☐ Mixed ☐

Number of patients with inflammatory rheumatism seen per months : _/_/_
Number of patients treated with biotherapy seen per months : _/_/_

- **PRACTICE : in patients with inflammatory rheumatism**

How frequently do you see patients affected with inflammatory rheumatism ? :
☐ every month ☐ every 3 months ☐ every 6 months
☐ Other Precise : ______

	Systematically	Very often	Sometimes	Not often	Never
**Do you give advice for :**					
Smoking cessation ?	☐	☐	☐	☐	☐
Nutrition ?					
Photoprotection ?	☐	☐	☐	☐	☐
**Do you search for skin cancer risk ?**	☐	☐	☐	☐	☐
In all your patients	☐	☐	☐	☐	☐
In patients affected with inflammatory rheumatism	☐	☐	☐	☐	☐
In case of treatment with biotherapy	☐	☐	☐	☐	☐

N° de rhumatologue :

- **PRACTICE : in patients treated with biotherapy**

**Before prescribing a biotherapy**						
Do you ask the patient about the appearance or modification of a skin lesion ?		Systematically ☐	Very often ☐	Sometimes ☐	Not often ☐	Never ☐
Do you perform a complete skin examination ?	Only in case of a skin lesion mentionned by the patient ☐	Systematically ☐	Very often ☐	Sometimes ☐	Not often ☐	Never ☐
Do you refer the patient to a dermatologist ?	Only in case of a skin lesion mentioned by the patient ☐	Systematically ☐	Very often ☐	Sometimes ☐	Not often ☐	Never ☐

**During the follow-up consultation**						
Do you ask the patient about the appearance or modification of a skin lesion ?		Systematically ☐	Very often ☐	Sometimes ☐	Not often ☐	Never ☐
Do you perform a complete skin examination ?	Only in case of a skin lesion mentioned by the patient ☐	Systematically ☐	Very often ☐	Sometimes ☐	Not often ☐	Never ☐
Do you refer the patient to a dermatologist once a year?		Yes ☐	No ☐	Other<br>Precise :		
Do you refer the patient to the GP for skin examination ?		Yes ☐	No ☐			

N° de rhumatologue :

➢ **PRACTICE : in patients with inflammatory rheumatism not treated with biotherapy**

**During the follow-up consultation**					
Do you ask the patient about the appearance or modification of a skin lesion ?	Systematically ☐	Very often ☐	Sometimes ☐	Not often ☐	Never ☐
Do you perform a complete skin examination ?					
Only in case of a skin lesion mentioned by the patient ☐	Systematically ☐	Very often ☐	Sometimes ☐	Not often ☐	Never ☐
Do you refer the patient to a dermatologist once a year?	Yes ☐	No ☐	Other<br>Precise :		
Do you refer the patient to the GP for skin examination ?	Yes ☐	No ☐			
